# Formicamycins, antibacterial polyketides produced by *Streptomyces formicae* isolated from African *Tetraponera* plant-ants†
†Electronic supplementary information (ESI) available: General remarks; full experimental details; Fig. S1–S6; Tables S1 and S2. See DOI: 10.1039/c6sc04265a


**DOI:** 10.1039/c6sc04265a

**Published:** 2017-02-13

**Authors:** Zhiwei Qin, John T. Munnoch, Rebecca Devine, Neil A. Holmes, Ryan F. Seipke, Karl A. Wilkinson, Barrie Wilkinson, Matthew I. Hutchings

**Affiliations:** a Department of Molecular Microbiology , John Innes Centre , Norwich Research Park , Norwich , NR4 7UH , UK . Email: barrie.wilkinson@jic.ac.uk; b School of Biological Sciences , University of East Anglia , Norwich Research Park , Norwich , NR4 7TJ , UK . Email: m.hutchings@uea.ac.uk; c School of Molecular and Cellular Biology , Astbury Centre for Structural Molecular Biology , University of Leeds , Leeds , LS2 9JT , UK; d Scientific Research Computing Unit , Department of Chemistry , University of Cape Town , Rondebosch 7701 , Cape Town , South Africa

## Abstract

We report a new *Streptomyces* species named *S. formicae* that was isolated from the African fungus-growing plant-ant *Tetraponera penzigi* and show that it produces novel pentacyclic polyketides that are active against MRSA and VRE. The chemical scaffold of these compounds, which we have called the formicamycins, is similar to the fasamycins identified from the heterologous expression of clones isolated from environmental DNA, but has significant differences that allow the scaffold to be decorated with up to four halogen atoms. We report the structures and bioactivities of 16 new molecules and show, using CRISPR/Cas9 genome editing, that biosynthesis of these compounds is encoded by a single type 2 polyketide synthase biosynthetic gene cluster in the *S. formicae* genome. Our work has identified the first antibiotic from the *Tetraponera* system and highlights the benefits of exploring unusual ecological niches for new actinomycete strains and novel natural products.

## Abstract 2

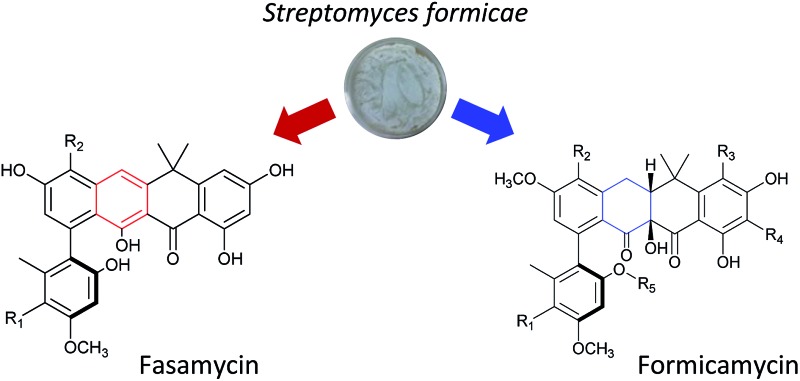
Ant pharming: antibacterial polyketides from plant-ant associated bacteria.

## Introduction

Over half of the antibiotics in clinical use are derived from the natural products (secondary metabolites) of *Streptomyces* bacteria and their close relatives, and most of these were introduced into the clinic during a ‘golden age’ of antibiotic discovery between 1940 and 1960.^1^ The misuse of antibiotics over the last 50 years has led to an alarming rise in antimicrobial resistance (AMR) which is arguably the greatest medical challenge humans will face this century. Recently, however, the advent of facile, large-scale genome sequencing and the discovery of new antibiotic-producing strains in under-explored environments has reinvigorated the field of natural products discovery. The wealth of genomic data now available has demonstrated that *Streptomyces* and other filamentous actinomycetes have the capacity to produce many more natural products than are identified after culturing in the laboratory: typically only 10–25% of their identifiable biosynthetic gene clusters (BGCs) are expressed under standard laboratory conditions and new classes of BGC remain to be discovered.^2,3^

We have been exploring the chemical ecology of protective mutualisms formed between actinomycete bacteria and fungus-growing insects in order to understand how these associations are formed and to explore this niche as a potential source of new antibiotics.^4^ In addition to the fungus-growing attine ants of South and Central America, which use actinomycete-derived antibiotics in their fungi-culture,^5,6^ it was recently discovered that many plant-ants also cultivate fungi.^7–9^ Plant-ants live in a mutualism with their host plant and provide protection from larger herbivores. In return, the host plants have evolved specialised hollow structures called domatia that house and protect the ants.^10^ South American *Allomerus* plant-ants and African *Tetraponera* plant-ants both grow fungi inside their domatia and they are associated with antibiotic-producing actinomycete bacteria.^11,12^

We previously reported the isolation of filamentous actinomycete bacteria, including *Streptomyces* and *Saccharopolyspora* strains, from the domatia and worker ants of *Tetraponera penzigi* plant-ants collected in Kenya.^12^ Genome sequencing of these strains allowed us to identify new species with genomes encoding novel and/or atypically large numbers of BGCs based on antiSMASH analysis.^13^ We consider strains containing significantly higher numbers of BGCs than typical strains (for *Streptomyces* sp. this is in the range 30–35) to be ‘talented’ with respect to their potential for yielding new natural products. One such organism, which we designate *Streptomyces formicae* KY5, also displayed a unique antagonistic activity against pathogenic drug resistant bacteria and fungi, including methicillin resistant *Staphylococcus aureus* (MRSA) and the multidrug resistant fungal pathogen *Lomentospora prolificans*.^14^ Subsequent bioassay guided fractionation using the sensitive test strain *Bacillus subtilis* led to the isolation and structural elucidation of thirteen new polyketide natural products that share a rare pentacyclic structure, some of which contain up to four chlorine atoms. These compounds fall into two groups. The first group (**1–3**) have an aromatic C-ring structure with sp^2^ carbon atoms at C10/C19, and lack any formal chiral centres. We have named these compounds fasamycin C–E respectively given their very close structural similarity to fasamycins A and B described previously from heterologous expression of a clone expressing a type 2 polyketide synthase (PKS) BGC isolated from an environmental DNA derived library.^15^ In contrast, compounds **4–13** are highly modified compared to the fasamycins with a non-aromatic C-ring and chiral centres at C10 and C19. We have named this group of compounds the formicamycins because they are the first natural products to be characterised from *S. formicae* and are structurally and biosynthetically distinct from the fasamycins (see below). Supplementation of the growth medium with sodium bromide resulted in the incorporation of bromine to yield three additional formicamycin congeners (**14–16**).

The formicamycins and fasamycins are active against clinical isolates of MRSA and vancomycin resistant *enterococci* (VRE), but do not display Gram-negative antibacterial or antifungal activity. The availability of sixteen congeners allowed their structure–activity relationship (SAR) to be examined. We then grew MRSA for 20 generations in the presence of sub-inhibitory concentrations of three formicamycins and re-determined the MICs for MRSA. These assays showed that MRSA does not easily acquire spontaneous resistance to formicamycins, at least under the conditions tested. Finally, we show, using CRISPR/Cas9 genome editing, that biosynthesis of these compounds is encoded by a type 2 PKS BGC in the *S. formicae* chromosome, and that re-introduction of this BGC restores biosynthesis of formicamycins in *S. formicae*. Identification of the formicamycin BGC allowed us to propose a plausible biosynthetic pathway. Deletion of *forV* encoding a putative flavin dependent halogenase abolished the production of any halogenated molecules and stalled the biosynthetic pathway at the fasamycin congener stage (**1–3**) indicating halogenation is a critical step required for further post-PKS modification to yield the formicamycin scaffold.

## Results and discussion

### Discovery of *Streptomyces formicae*: a talented new species

We previously isolated a number of filamentous actinomycete strains from the domatia and worker ants of the African *Tetraponera penzigi*-Acacia plant-ant mutualism.^12^ On the basis of 16S rDNA sequencing and morphological characteristics we chose six individual strains for genome sequencing using the Pacific Biosciences RSII platform with assembly using the HGAP2 pipeline. The resulting high-quality assemblies were analysed using the genome mining platform antiSMASH 3.0.^13^ One isolate in particular caught our attention as its genome harbours at least 39 BGCs and extracts derived from growth on agar plates showed promising bioactivities in anti-infective assays against *B. subtilis* and the fungal pathogens *Candida albicans* CA6 ^16^ and *Lomentospora prolificans* CBS116904 (see below). These results prompted us to examine the relative genetic relationship with sequenced streptomycetes, for which there are now more than 950 complete and draft genome sequences available (ESI Fig. S1†). On the basis of 16S RNA sequence analysis this strain possesses a unique lineage and is most closely related to *Streptomyces* sp. NRRL S-920, which was originally isolated from a soil sample of unknown origin. A more detailed comparison of *atpD*, *rpoB* and three other widely used phylogenetic markers, *gyrA* (DNA gyrase subunit A), *recA* (recombination protein) and *trpB* (tryptophan biosynthesis) revealed a 95% shared nucleotide identity between concatenated *atpD-gyrA-recA-rpoB-trpB* and *Streptomyces* sp. NRRL S-920, suggesting this strain represents a new species. Given that it was isolated from Kenyan *T. penzigi* worker ants, we suggest the name *Streptomyces formicae* KY5.

### *S. formicae* produces antibacterial and antifungal natural products

**Fig. 1:**
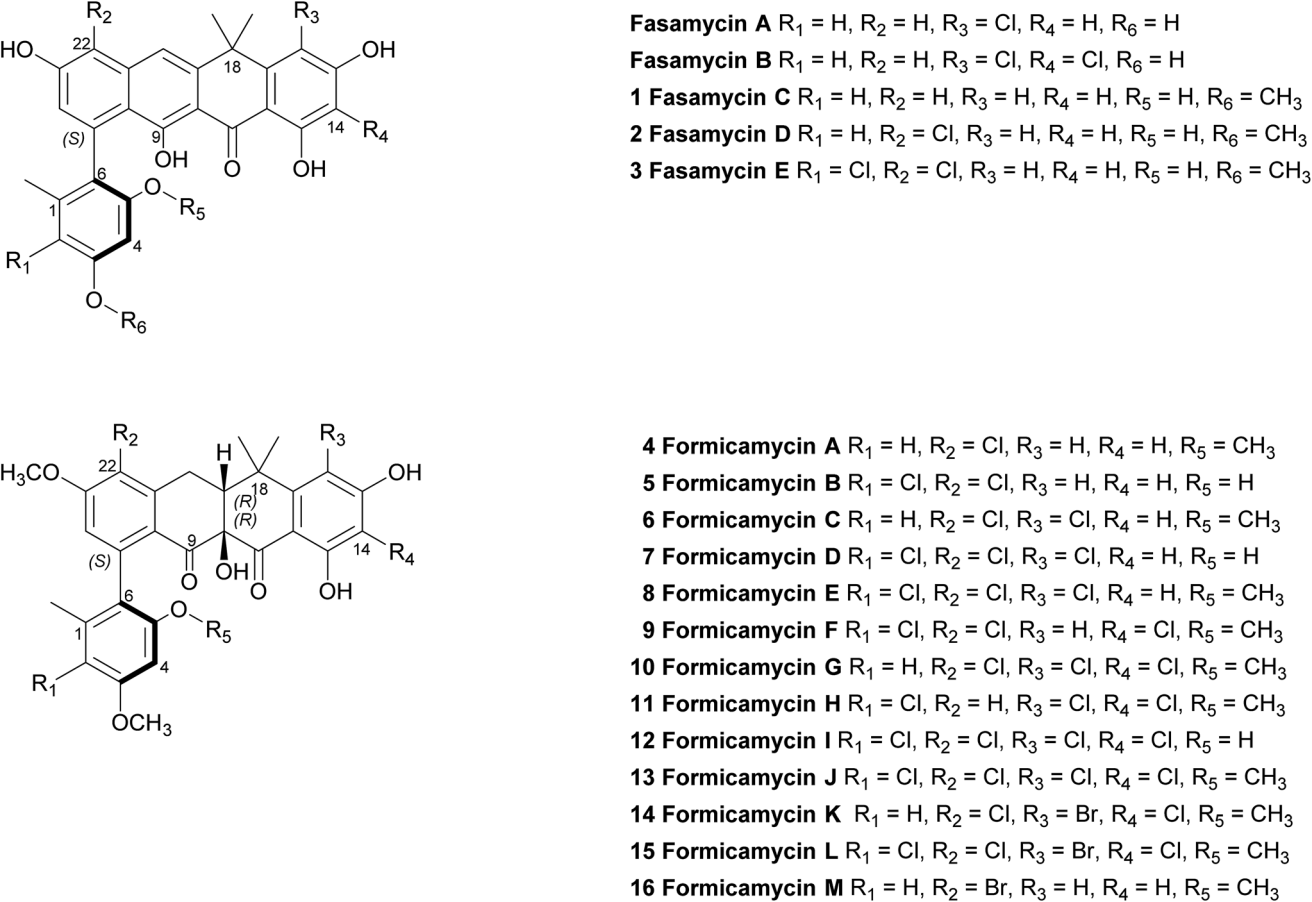
Structures of the previously reported fasamycins A & B, the new fasamycin congeners C–E (**1–3**) and the formicamycins A–M (**4–16**).

**Fig. 2:**
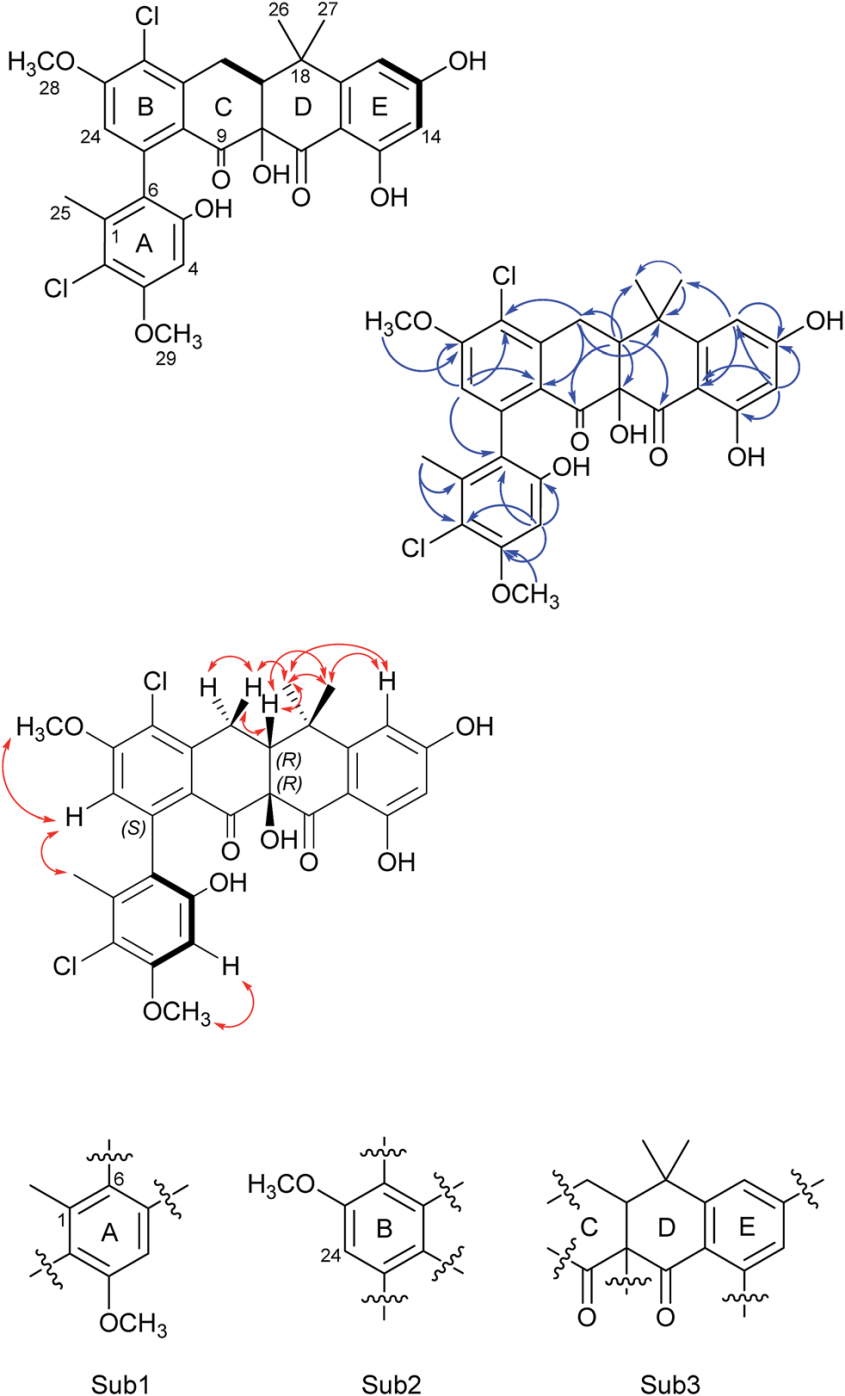
The COSY (bold), selected HMBC (blue arrows), and NOESY (red double-head arrow) correlations for formicamycin **5**. The resulting three substructures (Sub1–Sub3) are shown below, and each ring of the molecule is indicated (rings A–E).

Primary bioassays using *B. subtilis*, *C. albicans* and *L. prolificans* indicated that *S. formicae* produces compounds with antibacterial and antifungal activity when grown on solid medium. Fractionation over silica gel showed that these activities could be separated and high-resolution LCMS analysis suggested the presence of novel metabolites in the fractions exhibiting distinct antibacterial and antifungal activities. Very few agents have been described that are active against the emerging multidrug resistant fungal pathogen *L. prolificans*, and the isolation and characterization of the antifungal metabolites will be reported elsewhere. Further metabolomics analysis of the antibacterial fraction suggested a family of structurally related molecules (congeners) which correlated with the bioactivity against *B. subtilis*. In order to isolate sufficient material for detailed structural and biological analysis their production on MS agar was scaled up (as detailed in ESI†) to yield methanol extracts containing the target molecules. This included one experiment where the chemical elicitor sodium butyrate was added to the MS agar and led to the significantly enhanced production of the otherwise trace congener **1** (ESI Fig. S2†).^17^ Purification of the resulting extracts was achieved using a combination of normal phase, reversed-phase and size exclusion chromatography and led to the isolation of 13 individual molecules (**1–13**) in amounts of between 0.3 and 18 mg (see ESI† for full details). As there are several reports demonstrating that bromine can substitute for chlorine in microbial natural products, when provided to growing cultures at appropriate levels,^18,19^ we repeated the production experiment but grew *S. formicae* on MS agar containing sodium bromide (2 mM) and showed by LCMS that three new brominated congeners were produced (**14–16**). This experiment was scaled up and small amounts (<1 mg) of metabolites **14** and **15** were isolated while **16** was only detected by MS due to very low levels of production and the structure is inferred. The molecular formulae of all compounds **1–16** were measured using high-resolution MS and their chemical structures determined using 1D and 2D NMR spectroscopy as described below (see Fig. 1 and 2).

### Structural elucidation of the formicamycins and new fasamycins

Formicamycin B (**5**) was isolated first and its structure determined. The UV spectrum showed absorption maxima at 235 and 286 nm which is characteristic of all formicamycin congeners. High-resolution ESI-MS indicated a molecular formula of C_29_H_26_Cl_2_O_8_ (calculated [M + H]^+^ = 573.1077; observed [M + H]^+^ = 573.1072; *Δ* = –0.96 ppm), suggesting sixteen degrees of unsaturation. The ^13^C NMR data was consistent with this and showed two carbonyl carbons at *δ*_C_ 195.3 and 198.6 ppm, three methyl carbons at *δ*_C_ 18.3, 29.2 and 34.2 ppm, two methoxy signals at *δ*_C_ 56.3 and 57.3 ppm, one methylene at *δ*_C_ 30.2 ppm, and two signals consistent with sp^3^ quaternary carbons at *δ*_C_ 39.6 and 80.3 ppm. In addition, analysis of HSQC spectra indicated a sp^3^ tertiary carbon at *δ*_C_ 49.0 ppm which was hidden due to the solvent peak of deuterated methanol. It also showed 18 sp^2^ carbons at *δ*_C_ 98.7–168.0 ppm. The ^1^H NMR revealed the presence of five methyl singlets (*δ*_H_ 1.95, 1.40, 1.64, 3.63 and 3.90 ppm), two methylene proton double doublets (*δ*_H_ 2.77 ppm (dd, 18.94 Hz and 9.18 Hz) and 3.52 ppm (dd, 18.94 Hz and 6.66 Hz)), one aliphatic proton double doublet (*δ*_H_ 2.56 ppm (dd, 9.18 Hz and 6.66 Hz)), two aromatic proton singlets (*δ*_H_ 6.51 and 6.73 ppm), as well as two aromatic proton doublets (*δ*_H_ 6.14 ppm (d, 2.30 Hz) and 6.45 ppm (d, 2.29 Hz)). Analysis of the COSY spectrum gave limited data, meaning the majority of connections were made on the basis of HMBC correlations (Fig. 2). This led to three aromatic substructures consisting of all 29 carbon atoms, leaving the positions of two chlorine atoms and four hydroxyl groups unassigned. The signal at *δ*_C_ 80.3 ppm for C10 is consistent with a sp^3^ carbon and was assigned as a tertiary hydroxyl group. The signals for C5, C13, and C15 exhibit canonical phenol chemical shifts (*δ*_C_ 150–170 ppm). Substructures containing rings A and B were connected by a key HMBC correlation between H24 and C6. Similarly, the resulting ring-AB substructure is connected to ring-C (see substructure rings C–E, Fig. 2) by HMBC correlations between H20 and C8, C21 and C22, as well as the HMBC correlation between H19 and C21. The two chlorine atoms were therefore assigned to positions C2 and C22 (*δ*_C_ 113.9 and 121.8 ppm). The assignments are supported by the vicinal ^1^H–^1^H COSY correlations and NOESY correlations.

With the structure of **5** in hand we were able to readily assign the remaining structures as described in the ESI.† NOESY correlations allowed us to link the methoxy at C5 with H4 (*e.g.* **4**, **6**, **8–11** and **13**). We could also use NOESY correlations to distinguish H14 and H16 once one was chlorinated, depending on their relationship to the *gem*-dimethyl group (*e.g.* **7**, **8** and **9**).

In addition to the formicamycins **4–16**, we identified three related compounds (**1–3**) which lacked the two chiral centres at C10 (tertiary hydroxyl group) and C19 (bridgehead proton), and have an aromatic C-ring structure. These compounds were significantly more yellow than **4–16** with distinct UV spectra (with maxima at 246, 286, 353 and 418 nm) and exhibited significantly different optical rotations to the formicamycins. On the basis of these observations we assigned these compounds as new fasamycin congeners C–E (**1–3**) respectively. The fasamycins were first reported by Brady and co-workers in 2011 ^15,20^ and **1–3** represent new members of this family. We hypothesise that **1–3** represent biosynthetic precursors of the formicamycin biosynthetic pathway as discussed below.

To unambiguously assign the pentacyclic skeleton of these metabolites and confirm their polyketide origin, we performed a stable isotope labelling experiment. *S. formicae* was cultivated on MS agar (2 L) in the presence of [1,2-^13^C_2_] sodium acetate. After 7 days incubation the agar was extracted and the most abundant congener was isolated (compound **4**; 5 mg). The resulting ^13^C NMR spectra clearly indicated the intact incorporation of 12 acetate derived units, plus an enriched single carbon at C24, in a pattern consistent with a polyketide biosynthetic pathway (see ESI Fig. S3†).

### Stereochemistry of the fasamycins and formicamycins

Our NMR data alone did not allow configurational analysis of the two families of compounds to be completed. Although **1–3** lack any chiral centres they exhibit optical activity with [*α*]20D values in the range +18° to +27°; this optical activity is due to preferred structures generated by rotation about the chiral axis of the C6–C7 bond. Additionally, the formicamycins have chiral centres at C10 and C19 which leads to a shift in aromaticity of ring-C consistent with the distinct UV spectra of these compounds, and they exhibit much larger magnitude optical rotations.

To aid in determining their stereochemistry the electronic circular dichroism (ECD) spectra of fasamycin **3** and formicamycin **5** were calculated using time-dependent density functional theory (TDDFT). First, a systematic conformational analysis of each isomer was carried out using the MMFFs molecular mechanics force field *via* the Maestro software package.^21^ The conformers obtained within an energetic range of 3 kcal mol^–1^ of the lowest energy conformer were further optimized using the PBE1PBE^22^ exchange-correlation functional at the def2tzvp^23^ basis set level and with the SMD solvent model^24^ for methanol using the Gaussian09 program package.^25^ Frequency calculations were then carried out using these same settings to calculate the relevant percentage of the population of the conformers. The 30 lowest electronic transitions were then calculated using TDDFT and the rotational strengths of each electronic excitation were converted to ECD spectra using a Gaussian function with a half-bandwidth of 0.248 eV. The overall ECD spectra were then generated according to the Boltzmann weighting of each conformer.

**Fig. 3:**
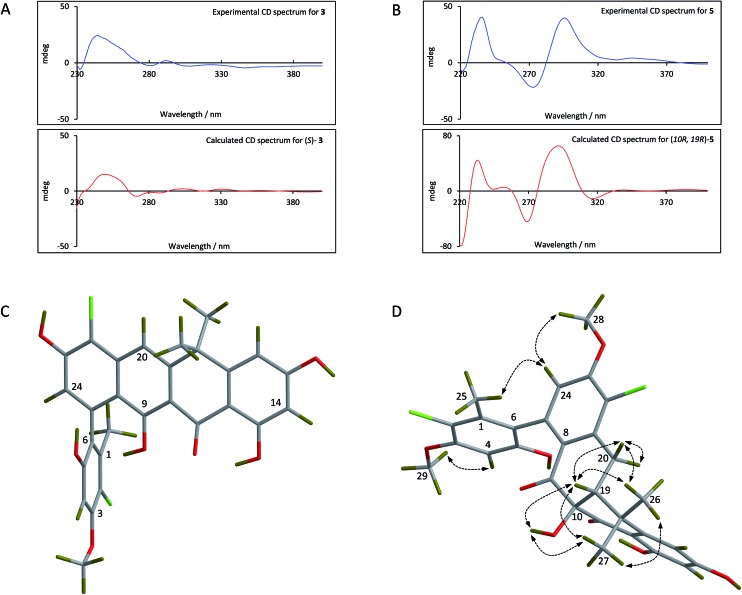
Comparison of the experimental and calculated CD spectra of **3** (A) and **5** (B), and the lowest energy conformers of (*S*)-**3** (C) and (10*R*,19*R*)-**5** (D). The key NOESY correlations for **5** are shown.

For the fasamycins, rotation about the C6–C7 axis means ring-A can be drawn with either the ortho hydroxyl or methyl group pointing forwards which correspond to the *S*- or *R*-configurations respectively. Comparison of the experimentally obtained ECD spectra for **3** to those calculated gives excellent agreement with that calculated for the *S*-configuration (Fig. 3A and ESI Fig. S3†) strongly suggesting this represents the preferred conformation.

For **5** we first compared the predicted structures for the lowest energy conformations of both the (10*RS*,19*RS*) and (10*SR*,19*RS*) diastereoisomeric pairs to data from NOSEY experiments. As observed in ESI Fig. S6† the (10*SR*,19*RS*) isomers with a *trans* relationship of the C10 and C19 substituents adopt an extended conformation of the four fused rings B–E. In contrast the *cis* (10*RS*,19*RS*) isomers are predicted to adopt a twisted L-shaped conformation (Fig. 3D). From this comparison the methine proton at C19 becomes diagnostic as the (10*RS*,19*RS*) isomers should show strong correlations to both methyl groups attached to C18 (methyl-26/27), whereas for the (10*SR*,19*RS*) isomers it should only give a correlation to methyl-27. Analysis of the NOESY data shows strong correlations for both methyl groups (26/27), and the remaining correlation data are also consistent with that expected for the (10*RS*,19*RS*) isomers (see Fig. 2 and 3D). We then acquired additional NMR datasets for **5** in non-protic solvent (*d*_6_-DMSO/*d*_3_-acetonitrile) and were able to locate the signal for the exchangeable hydroxyl proton at C10. Analysis of the NOESY spectrum showed clear correlations for this proton to the methine proton at C19 and methyl-27 which is compatible with the *cis* (10*RS*,19*RS*) isomers, but not the *trans* (10*SR*,19*RS*) isomers. NOESY data for the remaining formicamycin congeners was also consistent with the *cis* (10*RS*,19*RS*) configuration in each case. On this basis we were able to rule out the *trans* (10*SR*,19*RS*) isomers and proceeded to analyse the calculated and experimentally determined ECD spectra for the *cis* (10*R*,19*R*) and (10*S*,19*S*) enantiomers of **5** (Fig. 3B and ESI Fig. S4 and S5†). These data strongly suggested that the (10*R*,19*R*) stereochemistry was correct. Therefore, using combined NOESY NMR and ECD data we assign the (10*R*,19*R*) stereochemistry to the formicamycins. However, we are unable to make a definitive statement regarding the chiral C6–C7 axis for the formicamycins.

### Formicamycins exhibit potent activity against Gram-positive bacteria including drug resistant clinical isolates

**Fig. 4:**
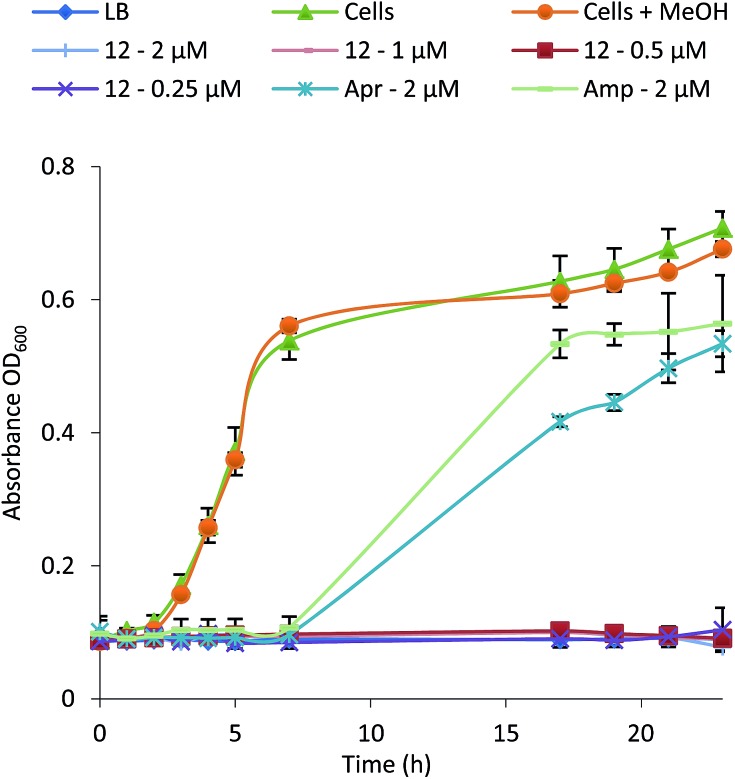
Growth inhibition curve and MIC determination for *Bacillus subtilis* in the presence of formicamycin **12** (Apr, apramycin; Amp, ampicillin).

To examine their structure activity relationship (SAR) we examined the growth of *B. subtilis* in liquid media supplemented with 0.01–100 μM of **1–15**. The MIC for each compound against *B. subtilis* is shown in Table 1 and the growth curve for one of the most potent (**12**) is shown in Fig. 4. All compounds effectively inhibit the growth of *B. subtilis* with an increase in potency observed for compounds containing an increasing number of chlorine atoms. Interestingly, brominated compounds appear to be slightly more potent than the equivalent chlorinated formicamycins. A shift from the fasamycin to formicamycin congeners also correlates with an increase in activity although it is unclear whether the ability to poly-halogenate this scaffold is the overriding factor.

**Table 1:** MIC data for **1–15** against *B. subtilis*, and MRSA and VRE clinical isolates. Values indicating “Not tested” or with “<” or “>” indicate issues with compound availability and a decision not to test further concentrations, *i.e.* they represent the lowest/highest concentrations tested

Compound	Minimum Inhibitory Concentration (μM)		
	*B. subtilis*	MRSA	VRE
**1**	<20	40	40
**2**	10	10	10
**3**	5	80	80
**4**	5	>80	>80
**5**	10	10	10
**6**	5	1.25	80
**7**	10	20	10
**8**	10	20	10
**9**	5	20	2.5
**10**	5	Not tested	Not tested
**11**	10	Not tested	Not tested
**12**	<2.5	<2.5	1.25
**13**	<20	0.625	1.25
**14**	<2.5	2.5	5
**15**	<2.5	1.25	2.5

To test whether **1–15** can inhibit drug-resistant Gram-positive bacteria we tested them against clinical isolates of MRSA and vancomycin-resistant *Enterococcus faecium* (VRE) (see ESI†) and found that the formicamycins are effective inhibitors of these organisms (Table 1). During the course of these experiments we observed that our test strains did not acquire spontaneous resistance when cultured on agar containing formicamycins. To test this further, we grew MRSA for four generations in the presence of no compound (control) and half MICs of compounds **6**, **13** and **15**. We then repeated the MIC tests and found no difference between the MRSA strains suggesting no resistance had arisen to formicamycins. We repeated the experiment but this time grew the strains for 20 generations and again found no increase in the MICs for these compounds, suggesting they exhibit a high barrier for the selection of resistant mutants, at least under the conditions tested here.

### Identification of the formicamycin BGC

**Fig. 5:**
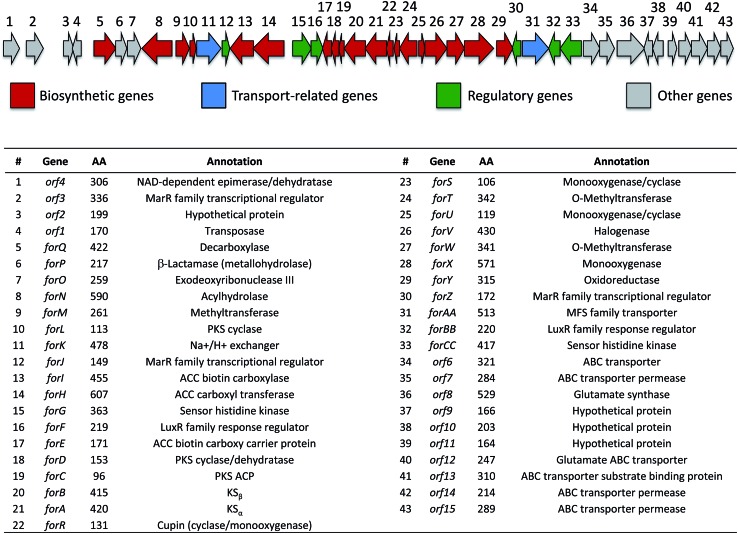
Organization of the formicamycin (*for*) BGC and annotation of putative gene products. ACC = acetyl-CoA carboxylase; PKS = polyketide synthase; MFS = major facilitator superfamily.

**Fig. 6:**
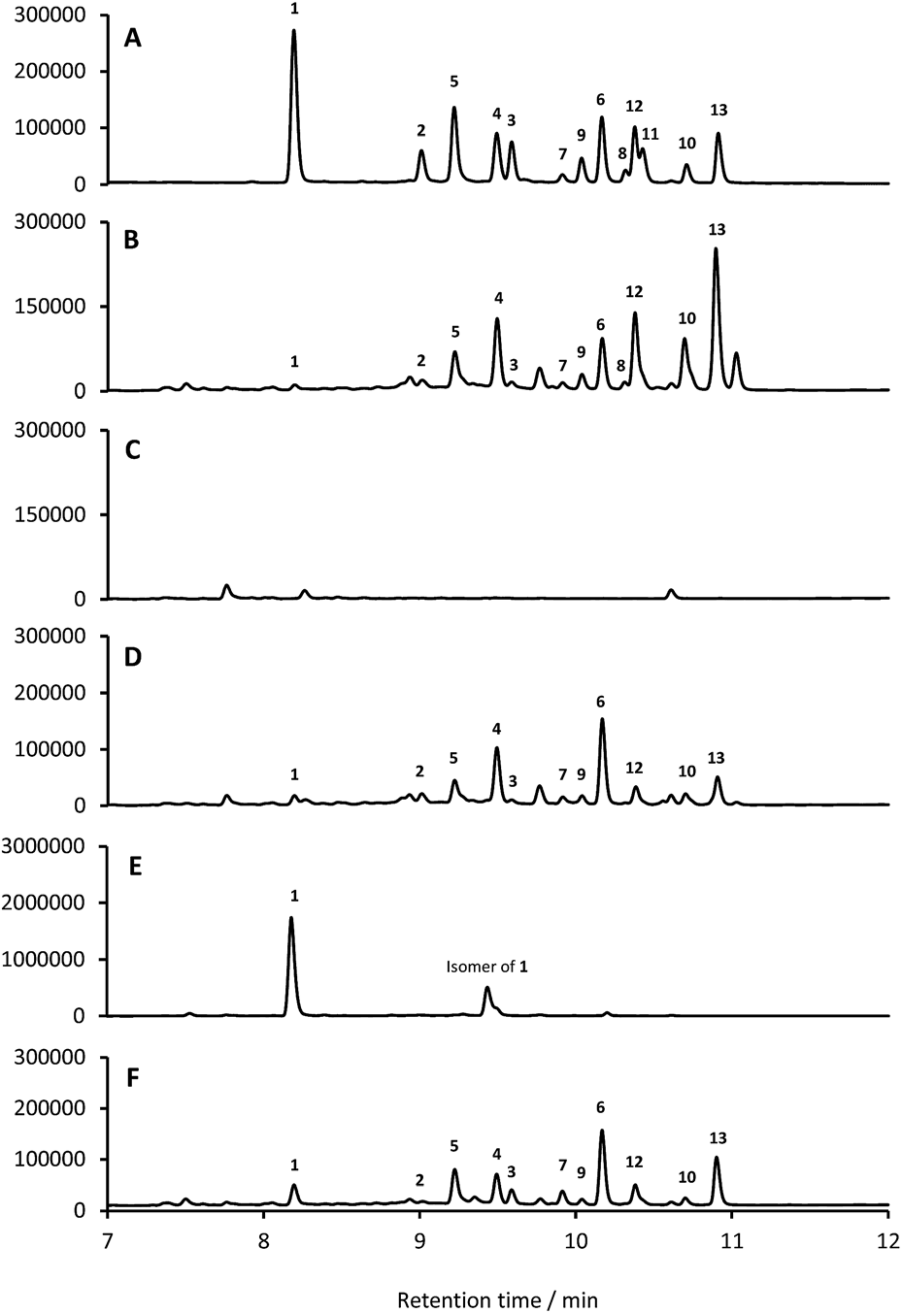
Deletion of BGC30 abolishes formicamycin biosynthesis, and *forV* encodes a halogenase gene. HPLC traces (250 nm) showing: (A) isolated **1–13** (mixed); (B) *S. formicae* wild-type; (C) *S. formicae Δfor*; (D) *S. formicae Δfor*/pESAC13-215-G; (E) *S. formicae ΔforV*; (F) *S. formicae ΔforV*/*forV*.

Based on their structures we predicted that biosynthesis of the formicamycins would be encoded by a BGC containing type 2 polyketide synthase (PKS) genes. Analysis of the *S. formicae* genome using antiSMASH 3.0 ^13^ identified only one type 2 PKS gene cluster (BGC30) which we designate *for* (Fig. 5; Table S2;† accession number: KX859301). We used the CRISPR/Cas9 vector pCRISPomyces-2 ^26^ to delete the entire BGC30 and surrounding genes in order to generate the unmarked deletion strain *S. formicae Δfor*; deletion of the BGC was confirmed by PCR amplification and sequencing (see ESI†). The wild-type strain and four independently generated *S. formicae Δfor* mutants were then grown in parallel under formicamycin producing conditions and subsequent LCMS(UV) analysis of extracts confirmed that fasamycin/formicamycins were not produced by the mutant strains (Fig. 6B and C). To ensure that loss of fasamycin/formicamycin biosynthesis was due to genome editing, and not other mutational events, we utilized a PAC (P1-derived artificial chromosome) library of the *S. formicae* genomic DNA which was custom made in pESAC13 by BioS&T Co. (Montreal, Canada). This was screened with three primer pairs (Table S1†), amplifying fragments either side and in the centre of BGC30. A single clone carrying the entire BGC30 (pESAC13-215-G) was introduced into one of the fasamycin/formicamycin-deficient mutants using tri-parental mating.^27^ LCMS(UV) analysis of the complemented strain alongside wild-type and mutant strains confirmed that fasamycin/formicamycin biosynthesis had been restored (Fig. 6D), and we conclude that BGC30 encodes the biosynthesis of compounds **1–13** in *S. formicae*.

### ForV is a halogenase required for formicamycin biosynthesis

Despite the identification of formicamycin congeners containing up to four halogen atoms we could identify only a single gene (*forV*) in BGC30 likely to encode a halogenase. Furthermore, analysis of the *S. formicae* genome identified only two further genes encoding potential halogenase enzymes that were associated with other BGCs (data not shown). ForV is a putative Flavin dependent halogenase, a family of enzymes which have been widely studied as catalysts involved in natural products biosynthesis,^28^ and a homologue of *forV* is present in the fasamycin BGC.^15,20^

To investigate its biosynthetic role we deleted the *forV* coding sequence using CRISPR-Cas9 methodology. Four independently isolated mutants were verified by PCR and sequencing, and extracts of the mutants grown on MS agar were analysed by LCMS(UV) (Fig. 6E). This showed accumulation of the non-halogenated fasamycin C (**1**) plus a new molecule with the same molecular formulae and UV spectrum indicating that it is a structural isomer of **1** (presumably bearing an *O*-methyl group at either C5 or C23 rather than at C3). The production levels of **1** by this mutant is approx. 188-fold that observed for the wild-type strain. Notably, no formicamycins could be observed in this extract. These data strongly suggest that ForV is responsible for the introduction of up to four halogen atoms. Genetic (*in trans*) complementation with the *forV* gene under the control of the native promoter re-established production of the halogenated compounds **2** and **3** and the formicamycins (Fig. 6F) indicating there was no polar effect or unanticipated genetic mutation introduced by the gene editing.

### Biosynthesis of the formicamycins

**Fig. 7:**
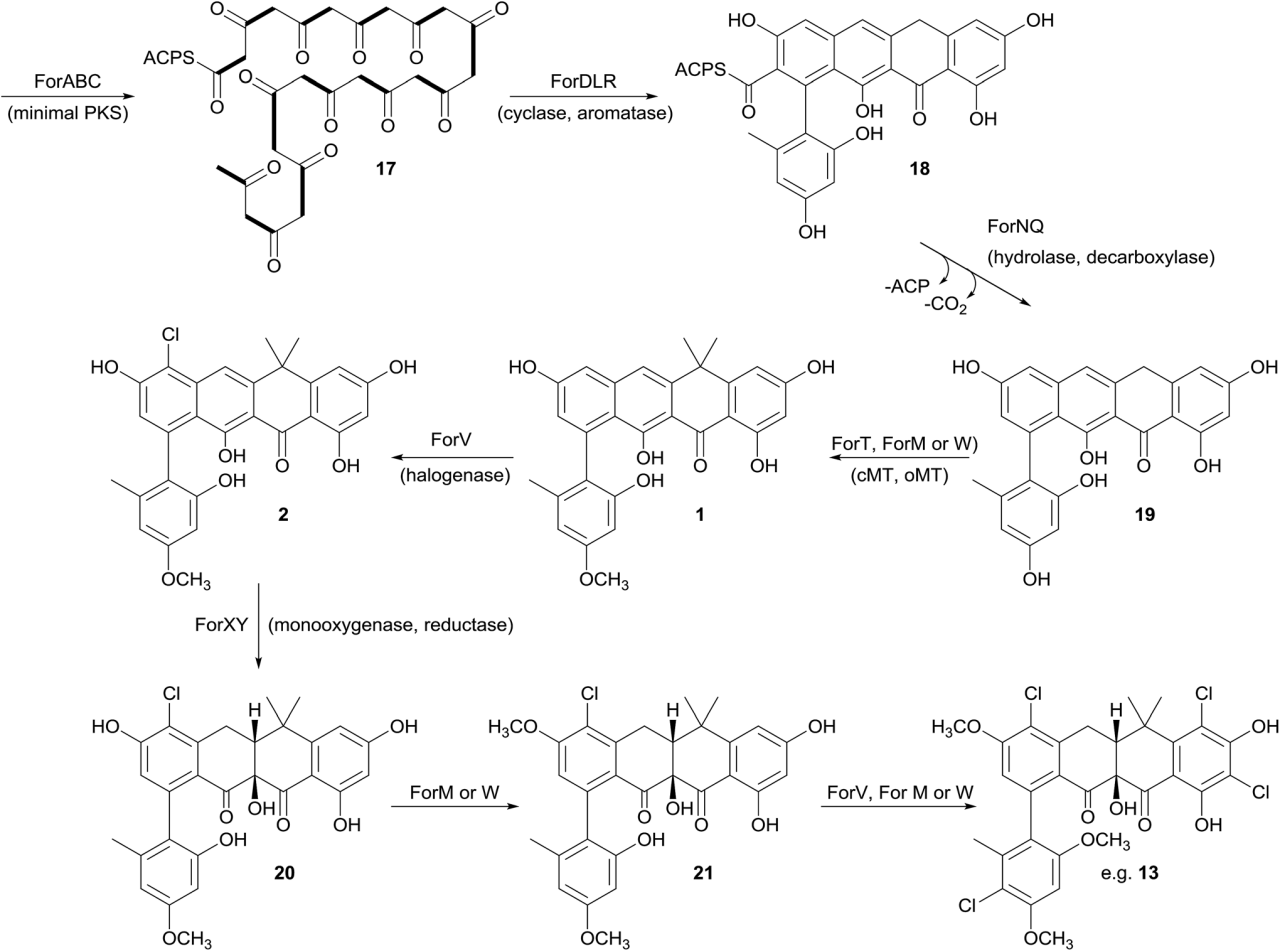
Proposed biosynthetic pathway for the fasamycins/formicamycins. Bold bonds in **17** indicate the positions of [1,2-^13^C_2_] sodium acetate units incorporated into the polyketide backbone. cMT, C-methyltransferase; oMT, O-methyltransferase.

Prior to this investigation no experiments regarding the biosynthesis of the fasamycins or formicamycins had been reported, although a pathway was proposed for the former based on sequencing of the fasamycin BGC and bioinformatics analysis.^15^ Based on the isotope feeding experiments, comparative bioinformatics and mutational analysis described above we are able to propose a biosynthetic pathway and assign putative functions to the BGC30 gene products (Fig. 5 and 7). Bacterial type 2 PKSs are characterized by a minimal set of gene products composed of the heterodimeric β-ketosynthase (KS) pair KS_α_/KS_β_ and an acyl carrier protein which are critical in determining polyketide chain length and the overall topology of the ring system to be made. We propose that ForABC comprise the minimal PKS and produce a tridecaketide intermediate **17** which, through the action of the putative additional tailoring enzymes including PKS cyclase/dehydratases (ForD, ForL, ForR), a hydrolase (ForN) and a decarboxylase (ForQ), is converted into **18** and then **19**.

All of **1–16** contain two methyl groups at C18 which, in conjunction with biosynthetic studies on the related pentangular polyketide benastatin,^29^ suggests that the first post-PKS step will involve installation of the *gem*-dimethyl group at C18. Three putative methyltransferases are encoded in BGC30 (ForM, ForT, and ForW), and ForT has the highest sequence shared identity with BenF (66%/49%; CAM58795.1) which catalyses the *gem*-dimethylation step during benastatin biosynthesis and is likely to catalyse the equivalent reaction during fasamycin/formicamycin biosynthesis; this gene is also present in the fasamycin BGC.^15^ Our inability to identify and isolate the putative intermediate **19**, or indeed any congeners lacking the *gem*-dimethyl moiety, leaves open the possibility that this molecule may not exist as an enzyme free intermediate and that ForT might actually act upon an ACP-bound intermediate which is then released and decarboxylated. Additionally, we did not isolate any congeners lacking a methoxy-group at C3 which suggests that *O*-methylation at this position occurs next and will be catalysed by one of the remaining methyltransferases ForM or ForW to yield **1**.

The accumulation of only **1** and a new isomer in the *forV* deletion mutant suggests that chlorination is the next step of the biosynthetic pathway and that it is essential to enable further post-PKS steps to occur in order to produce the formicamycins. This is consistent with the low levels of **1–3** observed from the wild-type organism, and analysis of the chlorination patterns for **2–13** suggests that chlorination at C2 or C22 is essential, with C22 likely being preferred to yield **2**.

Introduction of the tertiary hydroxyl group at C10 and modification of ring-C probably occurs next in the biosynthetic sequence. Moreover, as we only identified formicamycins containing both of these changes we propose that the transformations are linked, and may be catalyzed by the combined actions of the flavin dependent monooxygenase ForX and flavin dependent oxidoreductase ForY to yield **20**. A second *O*-methylation at C23 most likely occurs next (to give **21**) as all formicamycins contain this change. It is currently unclear when the final *O*-methylation at C5 occurs.

Finally, the most abundant formicamycin congeners contain either three or four chlorine atoms located on three different rings, and the minor congeners contain mostly two or three chlorine atoms distributed around the various locations; no fasamycins have a chlorine atom on ring E. These observations are consistent with the idea that ForV is a promiscuous enzyme capable of catalysing up to four halogenation reactions on a single molecule, but that there is a preferred, but not absolute, ordering to these modifications.

Comparison to the fasamycin BGC^15^ fails to identify homologues of certain genes present in BGC30 that we propose may be involved in formicamycin biosynthesis. In contrast others are present in both BGCs that we suggest may be responsible for some of the structural differences observed. Plausible reasons for these differences include differential expression, or a lack of expression in one species, and the involvement of genes that were not captured on the expression cosmid used for production of the fasamycins.^15^ To address these questions a detailed study of formicamycin biosynthesis is underway in our labs.

## Conclusions

Most of the antibiotics in clinical use are derived from the natural products of soil microbes, most notably species of *Streptomyces* bacteria that were discovered more than 50 years ago. Here we highlight how searching under-explored environments combined with new advances in genome sequencing and editing enables the discovery of new species making natural products with potent anti-infective activity that could bypass resistance and form the basis of new anti-infective therapies. Specifically, we identified a new species, *Streptomyces formicae*, from the African plant-ant *Tetraponera penzigi*, and show that it makes a family of rare pentangular polyketide antibiotics. These new molecules, which we call the formicamycins, inhibit the growth of the clinically relevant pathogens MRSA and VRE. The formicamycins are more potent than the previously reported and structurally related fasamycins.^15,20^ Spontaneous resistance to fasamycins was used to identify their molecular target but our data suggest that the formicamycins have a higher barrier for the selection of resistant mutants, at least for MRSA, under the conditions examined here. The reason for increased potency of the poly-halogenated congeners may simply be due to increased lipophilicity and an enhanced ability to cross the bacterial cell membrane. Moreover, docking studies reported during the previous work on fasamycins mode of action suggest that the chloro-*gem*-dimethyl-anthracenone substructure represents the key pharmacophore.^20^ This region comprises the key structural differences between the two chemotypes as exemplified by the three dimensional structure presented in Fig. 3 and it is currently unclear whether their molecular target and mode of action may differ. This will be addressed in future studies.

Intriguingly, bioinformatics analysis shows that the formicamycin BGC is closely related to an unassigned BGC present in the genome of *Streptomyces kanamyceticus* (Genbank ID LIQU00000000.1). Further, an approx. 188 kbp region of the *S. formicae* genome, which encompasses BGC30, is syntenic with the *S. kanamyceticus* genome (extending approx. 64 kbp upstream and at least 95 kbp downstream, which is as far as the contig LIQU01000034 extends) and we suggest there has been a horizontal gene transfer event. Further bioinformatics analysis and consideration of the biosynthetic pathway leads us to propose that *forQ* and *forCC* represent the boundaries of BGC30 (Fig. 4). Additionally, the region of sequence encoding *forX* to *forAA*, which is not present on the *S. kanamyceticus* genome, comprises gene sequences with closest homologues in *Actinomadura* species, and appears to have been inserted into the *S. kanamyceticus* syntenic sequence. This suggests the formicamycin BGC may have its origin in multiple horizontal transfer events. Further work, both to understand the origins of the formicamycin BGC, and to delineate their biosynthesis, are underway in our laboratories. We anticipate this data will aid in the application of biosynthetic medicinal chemistry methods to produce further improved molecules with potential application as antibacterial agents.

## Materials and methods

For details regarding experimental procedures, spectroscopic and chromatographic data, microbiology and molecular biology procedures, genome sequencing and the proposed function of gene products, see the ESI.†
