# Supplementary material for: Formicamycins, antibacterial polyketides produced by Streptomyces formicae isolated from African Tetraponera plant-ants
Source: Chem Sci. 2017 Feb 13;8(4):3218–27. doi: 10.1039/c6sc04265a (PMC5414599; doi:10.1039/c6sc04265a)
Supplement: Supplementary file 1 [file SC-008-C6SC04265A-s001.pdf]

Electronic Supplementary Material (ESI) for Chemical Science.
This journal is © The Royal Society of Chemistry 2017

ELECTRONIC SUPPLEMENTARY INFORMATION

Formicamycins, antibacterial polyketides produced by *Streptomyces formicae* isolated from African *Tetraponera* plant-ants

Zhiwei Qin[1], John T. Munnoch[2], Rebecca Devine[2], Neil A. Holmes[2], Ryan F. Seipke[3], Karl A. Wilkinson[4], Barrie Wilkinson[1]* and Matthew I. Hutchings[2]*

[1]Department of Molecular Microbiology, John Innes Centre, Norwich Research Park, Norwich, NR4 7UH, United Kingdom. E-mail: barrie.wilkinson@jic.ac.uk

[2]School of Biological Sciences, University of East Anglia, Norwich Research Park, Norwich, NR4 7TJ, United Kingdom. E-mail: m.hutchings@uea.ac.uk or @matthutchings10

[3]School of Molecular and Cellular Biology, Astbury Centre for Structural Molecular Biology, University of Leeds, Leeds, LS2 9JT, United Kingdom.

[4]Scientific Research Computing Unit and Department of Chemistry, University of Cape Town, Rondebosch 7701, Cape Town, South Africa.

*Corresponding authors

Table of contents

1. Experimental:
    a. Standard chemistry methods and materials
    b. Standard microbiology and molecular biology methods
    c. Genome sequencing, BGC annotation and bioinformatics analysis
    d. Phylogenetic analysis of Streptomyces formicae
    e. Production, purification and structure elucidation of formicamycins
    f. Stable isotope labelling experiment
    g. MIC determination
    h. CRISPR/Cas9 genome editing
    i. Ectopic expression of BGC30
    j. Genetic complementation of ForV
2. ESI Figures
3. ESI Tables
4. ESI References

1. Experimental

1a. Standard chemistry methods and materials. Unless stated otherwise all chemicals were supplied by Sigma-Aldrich or Fisher Scientific. [1,2-$^{13}C_2$] sodium acetate was purchased from Cambridge Isotope Laboratories, Inc. All solvents were of HPLC grade or equivalent. NMR spectra were recorded on a Bruker Avance III 400 MHz NMR spectrometer equipped with 5 mm BBFO Plus probe. Optical rotations were measured on a PerkinElmer Polarimeter (Model 341) using the sodium D line (589 nm) at 20°C and the specific rotation was then calculated accordingly.

Unless otherwise stated samples were analysed by LCMS/MS on a Nexera/Prominence UHPLC system attached to a Shimadzu ion-trap time-of-flight (IT-ToF) mass spectrometer. The spray chamber conditions were: heat-block, 300°C; 250° curved desorbation line; 1.5 L/min nebulizer gas; drying gas on. The instrument was calibrated using sodium trifluoroacetate cluster ions according to the manufacturer's instructions. The following analytical LCMS method was used throughout this study: Phenomenex Kinetex C_{18} column (100 × 2.1 mm, 100 Å); mobile phase A: water + 0.1% formic acid; mobile phase B: methanol. Elution gradient: 0–1 min, 20% B; 1–12 min, 20%–100% B; 12–14 min, 100% B; 14–14.1 min, 100%–20% B; 14.1–17 min, 20% B; flow rate 0.6 mL/min; injection volume 10 µL; positive-negative mode switching; nubulizing gas flow rate: 1.5 L/min; interface (probe) voltage: 4.5 KV.

1b. Standard microbiology and molecular biology methods. *Escherichia coli* strains were cultivated at 37°C using Lennox Broth (LB) or LB agar [1]. *S. formicae* was cultivated at 30°C on soya flour-mannitol (MS) agar supplemented with the relevant antibiotics. Chromosomal DNA was isolated using the salting out method [2]. The pESAC13 (ePAC) clone library was prepared by Bio S&T Inc. (Montreal, Canada) and clones containing the *for* BGC were identified using primers shown in ESI Table 1, designed to amplify fragments from both ends and the centre of the biosynthetic gene cluster (BGC).

1c. Genome sequencing, BGC annotation and bioinformatics analysis. DNA was sequenced with Pacific Biosciences (PacBio) RSII SMRT technology at The Earlham Institute, Norwich Research Park, Norwich UK and assembled using the HGAP2 pipeline. The assembly obtained 4 contigs, one larger fragment of 9.3 Mb and three fragments of 179 kbp, 111 kbp and 27 kbp. The sequence of the formicamycin BGC has been deposited at Genbank under the accession number KX859301. Protein coding sequences (PCSs) in the *for* gene cluster were identified using Prodigal through antiSMASH and genes were manually annotated according to BLAST searches [3]. The BGCs identified in the *S. formicae* genome are shown in ESI Table 2.

1d. Phylogenetic analysis of *Streptomyces formicae*. A *Streptomyces* phylogeny was inferred from concatenated partial DNA sequences for *atpD and rpoB*, as previously described [4]. DNA sequences were retrieved from Genbank and aligned using eight iterations of the Geneious 8.1.4 implementation of Muscle 3.8.31 [5]. Sequences were subsequently trimmed to the same length (including gaps) and concatenated in the order: *atpD-rpoB*. Phylogenetic relationships were inferred from the concatenated sequences by approximate maximum likelihood analysis using FastTree 2.1.7 [6]. *Mycobacterium tuberculosis* H37Rv was used as an outgroup and FigTree 1.4.1 was used to visualize the tree. Concatenated *aptD-rpoB* sequences were grouped into operational taxonomic units (OTUs) using the MacQiime v1.80 implementation of UCLUST with shared identity thresholds of 93, 95 and 97%.

The 16S rDNA gene only provides an accurate and reliable classification to the genus level, likely due to extension recombination in the evolutionary past [4]. Thus, we selected two protein-encoding loci, *atpD* (ATP synthase) and *rpoB* (RNA polymerase subunit B), both of which have been used extensively to infer phylogenies of streptomycetes [7]. Partial DNA sequences for *atpD* and *rpoB* corresponding to nucleotides 475-874 of *S. coelicolor atpD* (*sco5373*) and 1471-1746 of *rpoB* (*sco4654*) were retrieved from Genbank for as many streptomycetes as possible. An approximate maximum-likelihood phylogenetic tree based on concatenated *atpD-rpoB* gene fragments (746 nucleotides in total) was constructed (ESI Fig. 1). There was good separation and statistical support for most branches in the resulting tree and the overall topology is in agreement with recently reported streptomycete phylogenies [4].

1e. Production, purification and structure elucidation of formicamycins. To isolate compound **5**, *Streptomyces formicae* was cultivated on MS agar (20 L; approx. 600 plates) at 30°C for seven days. The agar was sliced into small pieces and extracted once with methanol (10 L) using ultrasonication to improve the extraction. The crude methanolic extract was filtered and concentrated by evaporation then diluted with deionized water to approximately 500 mL and extracted with ethyl acetate (3 x 500 mL). The extracts were combined and the solvent removed under reduced pressure to yield a brown oil which was dissolved in acetone (50 mL) and mixed with flash silica gel (20 g; 60 Å, 60–100 mesh) and the solvent removed under reduced pressure. The impregnated silica gel was then loaded onto an open column (6 cm × 20 cm) preloaded with flash silica in chloroform. Elution was achieved using first 100% chloroform (600 mL) and then a stepped gradient of chloroform:methanol using the following volumetric ratios: 19:1, 9:1, 8:2, 7:3, 6:4 and then 100% methanol (600 mL each). The solvent was removed from each fraction (400 mL of each sample), the residue dissolved in methanol (10 mL) and this was tested for antibacterial activity using a disk diffusion assay against *B. subtilis*. The antibacterial activity was located exclusively in fraction 3 which was further separated by chromatography over a Phenomenex Gemini-NX reversed-phase column

(C_{18}, 110 Å, 150 × 21.2 mm) using a Thermo Scientific Dionex Ultimate 3000 HPLC system and eluting with the following gradient method: (mobile phase A: water + 0.1% formic acid; mobile phase B: methanol) 0–5 min 40% B; 5–35 min 40%-100% B; 35–40 min 100% B; 40–40.1 min 100%-40% B; and 40.1–45 min 40% B; flowrate 20 mL/min; injection volume 500 µL. Absorbance was monitored at 280 nm. Fractions (20 mL) were collected and analysed by LCMS. Fraction 24 containing **5** was further purified by chromatography over a Phenomenex Gemini-NX semi-prep reversed-phase column (C_{18}, 110 Å, 150 × 10 mm) using a Agilent 1100 series HPLC system and eluting with the following gradient method: (mobile phase A: water + 0.1% formic acid; mobile phase B: methanol) 0–2 min 50% B; 2–10 min 50%–80% B; 10–11 min 80% B; 11–12 min 80%–50% B; 12–14 min 50% B; flowrate 3 mL/min; injection volume 100 µL). Absorbance was monitored at 280 nm. The resulting yellow solid (**5**; 18 mg) was subjected to analysis by HRMS and NMR as described in the main text and the structure determined as shown in Fig. 1 and Fig. 2 of the main paper.

Compounds **2-4**, **6-13** were isolated from approximate 8.6 L of MS agar using the same methods as above except that **10-13** were additionally purified by Sephadex LH20 size exclusion chromatography with 100% methanol as the mobile phase. The isolated yields were: **2** (3 mg), **3** (2 mg), **4** (6 mg), **6** (12 mg), **7** (2 mg), **8** (18 mg), **9** (3 mg), **10** (1 mg), **11** 0.3 mg), **12** (2 mg) and **13** (3 mg). The structure of these compounds was readily assigned based on comparisons to the NMR data for **5** (see main text). The trichlorinated compounds **8-11** are isomers with the same molecular formula but different retention times when isolated by HPLC. Further NMR analysis indicates that chlorination can occur at C4 and C16 in addition to C2 and C22: proton-proton coupling constants provide the key information to distinguish these compounds. For example, formicamycin **8** and **9** do not possess adjacent protons and therefore only three singlet peaks appear in the ^{1}H NMR spectrum. For formicamycins **10** and **11** each have a pair of adjacent protons resulting in two doublet peaks in the ^{1}H NMR spectrum. Compound **2** and **3** were also assigned on the basis of the 2D NMR and comparison of ^{13}C NMR data with that of **5**. For example the disappearance of the signals for the carbonyl at C9, the SP^3 tertiary carbons at C10 and C19, and as well as the tertiary methylene at C20. For compounds **2** and **3** these signals all indicate SP^2 carbons suggesting an aromatic ring C. The proposed structures are further supported by UV spectral and optical rotation data. **1** was isolated from *S. formicae* grown on MS agar containing sodium butyrate (1.35 L at 10 mM; 1.35 L at 50 mM). The effect of sodium butyrate on the production levels of **1** can be seen in ESI Fig. 2. For this experiment the following HPLC(UV) method was used: Agilent 1100 series HPLC system equipped with a Phenomenex Gemini-NX C_{18} column (110 Å, 150 × 4.6 mm); mobile phase A: water + 0.1% formic acid; mobile phase B: methanol. Elution gradient: 0–3 min, 70% B; 3–20 min, 70%–100% B; 20–23 min, 100% B;

23–24 min, 100%–70% B; 24–28 min, 70% B; flowrate 1 mL/min; injection volume 20 µL. Growth, extraction and purification conditions were as described above to yield **1** (2 mg). The structure was readily assigned on the basis of 2D NMR and comparison of the ^{1}H NMR data with that for **2** and **3**.

14 and **15** were isolated from growth on MS agar (3 L) containing NaBr (2 mM) after 20 days. Extraction and purification was performed as described above to yield **14** (0.5 mg) and **15** (0.6 mg) To confirm the site of bromination, comparison of the **10** and **14** ^{1}H NMR chemical shifts showed that H26 and H27 are shifted slightly downfield for **14**. No other significant differences could be identified and we concluded that the bromine atom of **14** is located at C16. Similar comparisons for **15** with **13** again allowed us to assign the location of the bromine atom.

Fasamycin C (1)

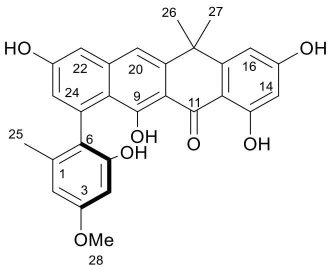

Molecular formula: $C_{28}H_{24}O_7$

Isolated yield: 2 mg

UV (PDA): λ_{max} = 247, 287, 353 and 418 nm

Optical activity: $[\alpha]_D^{20}$ = +18.08

HRMS (ESI) *m/z*: calculated $[M + H]^+$ = 473.1595, observed $[M + H]^+$ = 473.1602, Δ = 1.48 ppm

NMR (CD_3OD; 400 MHz & 100 MHz): ^{1}H, ^{13}C, HSQC, HMBC, NOESY

Position	δ_C ppm	δ_H ppm (no. of protons, multiplicity, J in Hz)	HMBC	NOESY
1	138.1		25	
2	109.4	6.3 (1H, s)	4, 25	25, 28
3	159.0		28	
4	97.6	6.3 (1H, s)	2	28
5	158.1		4	
6	126.1		2, 4, 24, 25	
7	141.2			

8	118.2		20, 22, 24	
9	166.9			
10	107.7		20	
11	191.8			
12	108.8		16	
13	166.6			
14	102.2	6.2 (1H, d, 2.28)	16	
15	167.0		16	
16	107.2	6.7 (1H, t, 2.60)		26, 27
17	156.0		26, 27	
18	39.9		16, 26, 27	
19	146.6		26, 27	
20	116.3	7.4 (1H, s)	22	22, 26, 27
21	143.0		20	
22	110.1	7.0 (1H, d, 2.48)	20	20
23	160.2		22	
24	122.4	6.7 (1H, t, 2.60)	22	25
25	20.8	1.9 (3H, s)	2	2, 24
26	34.8	1.7 (3H, s)	27	16, 20
27	34.9	1.7 (3H, s)	26	16, 20
28	56.2	3.6 (3H, s)		2, 4

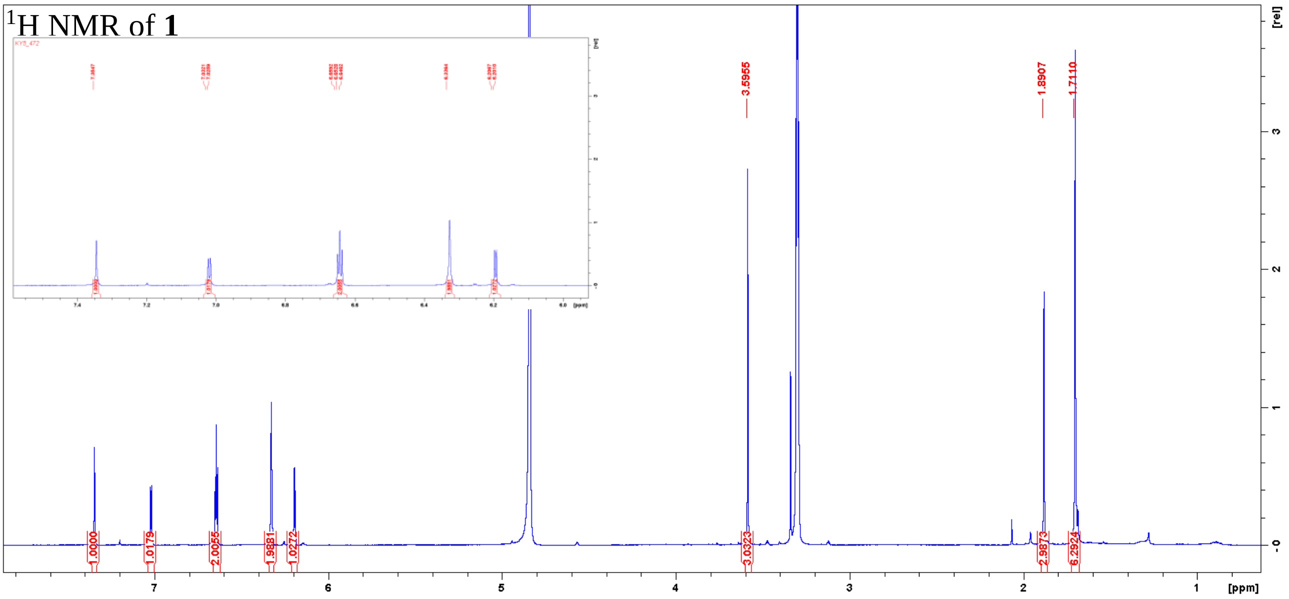

^{1}H NMR of **1**

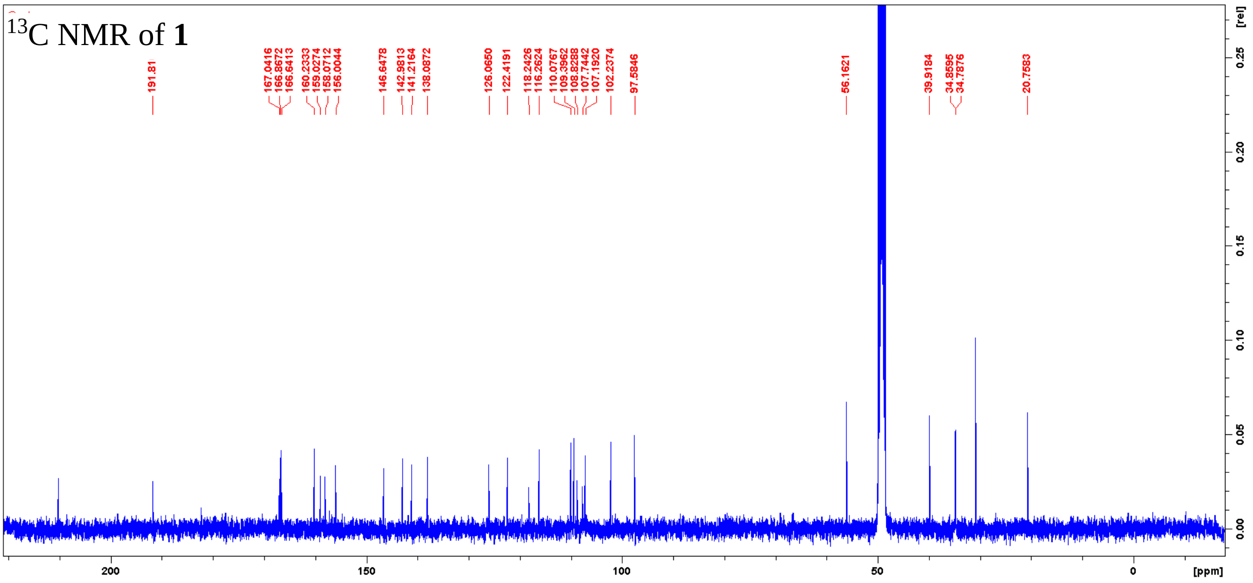
13C NMR of 1
191.81
167.0416
166.8672
166.6413
160.2333
159.0274
158.0712
156.0044
146.6478
142.9813
141.2164
138.0872
126.0650
122.4191
118.2426
116.2624
110.0767
109.3962
108.8288
107.7442
107.1920
102.2374
97.5846
56.1621
39.9184
34.8595
34.7876
20.7583
[rel]
[ppm]

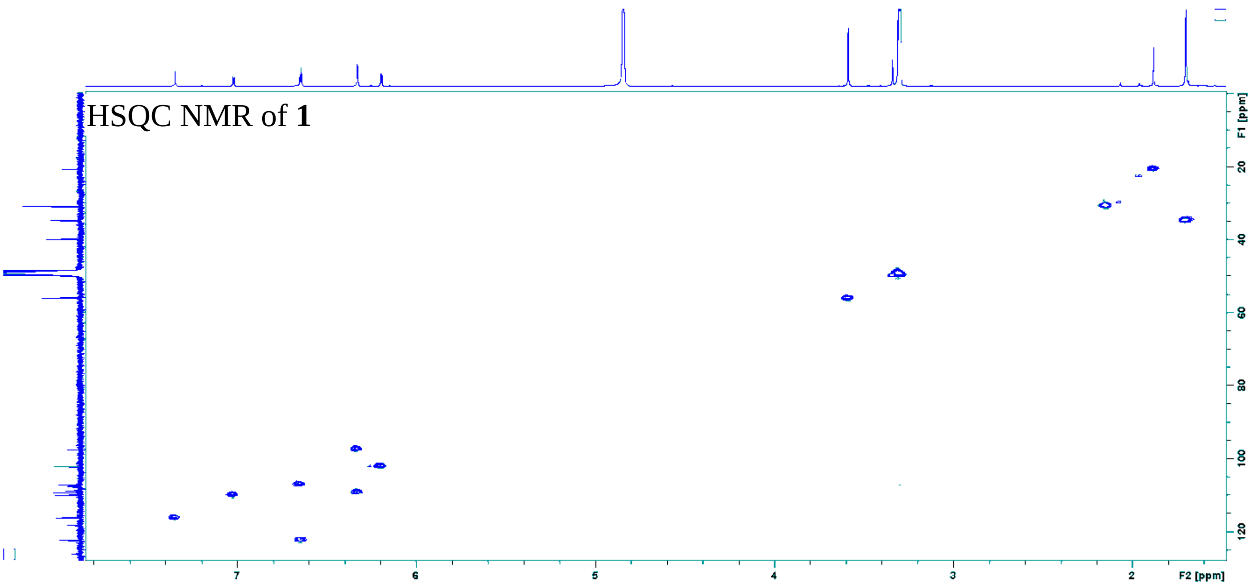
HSQC NMR of 1
F1 [ppm]
F2 [ppm]

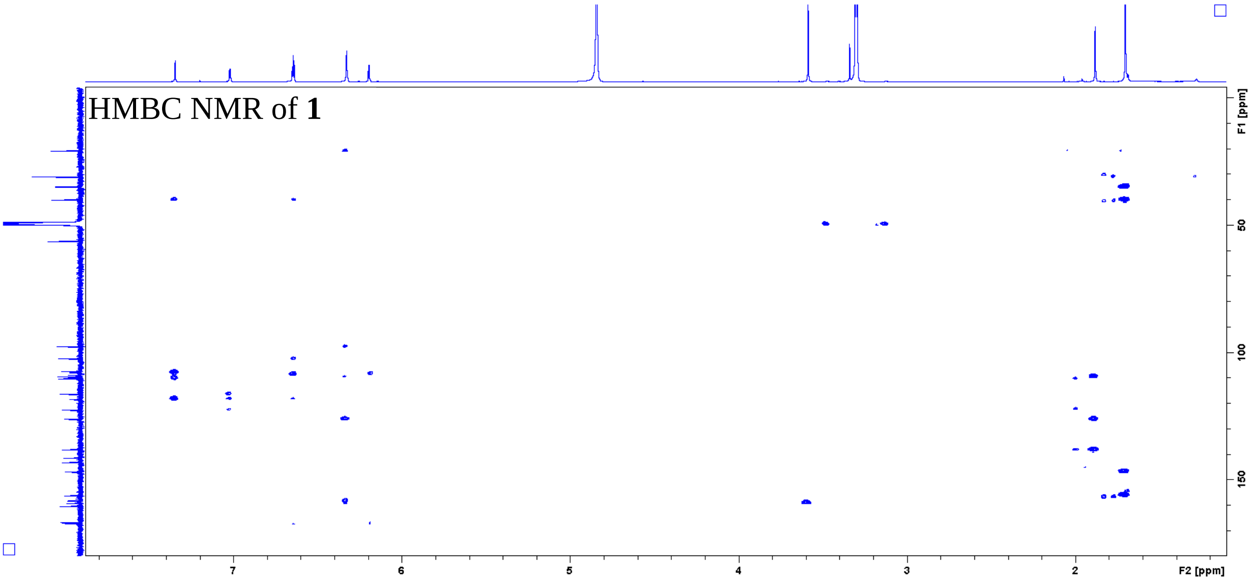

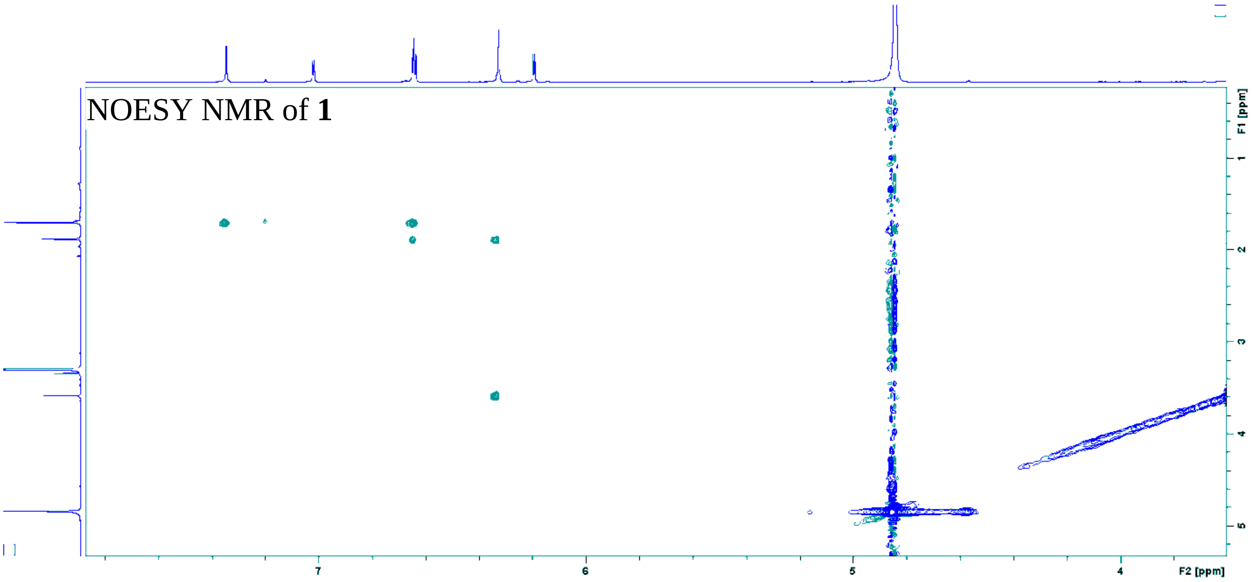

Fasamycin D (2)

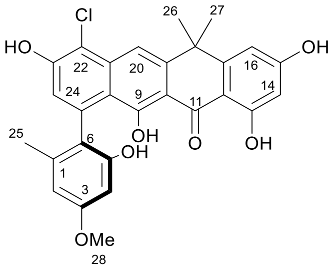

Molecular formula: $C_{28}H_{23}ClO_7$

Isolated yield: 3 mg

UV (PDA): λ_{max} = 249, 288, 351 and 416 nm

Optical activity: $[\alpha]_D^{20}$ = +18.88

HRMS (ESI) *m/z*: calculated $[M + H]^+$ = 507.1205, observed $[M + H]^+$ = 507.1214, Δ = 1.77 ppm

NMR (CD_3OD; 400 MHz & 100 MHz): ^1H, ^{13}C, HSQC, HMBC, NOESY

Position	δ_C ppm	δ_H ppm (no. of protons, multiplicity, J in Hz)	HMBC	NOESY
1	138.0		25	
2	109.5	6.3 (1H, s)	4, 24	25
3	158.3		2, 4	
4	97.6	6.3 (1H, s)	2	28
5	156.0			
6	125.5		2, 4, 24, 25	
7	138.7			
8	119.1		20,24	
9	167.0			
10	108.1		20	
11	191.9			
12	108.8		14, 16	
13	166.7			
14	102.4	6.2 (1H, dd, 2.33)	16	
15	167.1		14	
16	107.4	6.7 (1H, dd, 2.33)	14	26, 27
17	155.7		26, 27	
18	40.3		16, 20, 26, 27	
19	148.3		26, 27	
20	112.3	7.9 (1H, s)		26, 27
21	139.6			
22	114.0		20, 24	
23	159.0			
24	122.0	6.8 (1H, s)		25
25	20.7	1.9 (3H, s)	2	2, 24
26	35.1	1.7 (3H, s)	27	16, 20
27	35.1	1.7 (3H, s)	26	16, 20
28	56.2	3.6 (3H, s)		4

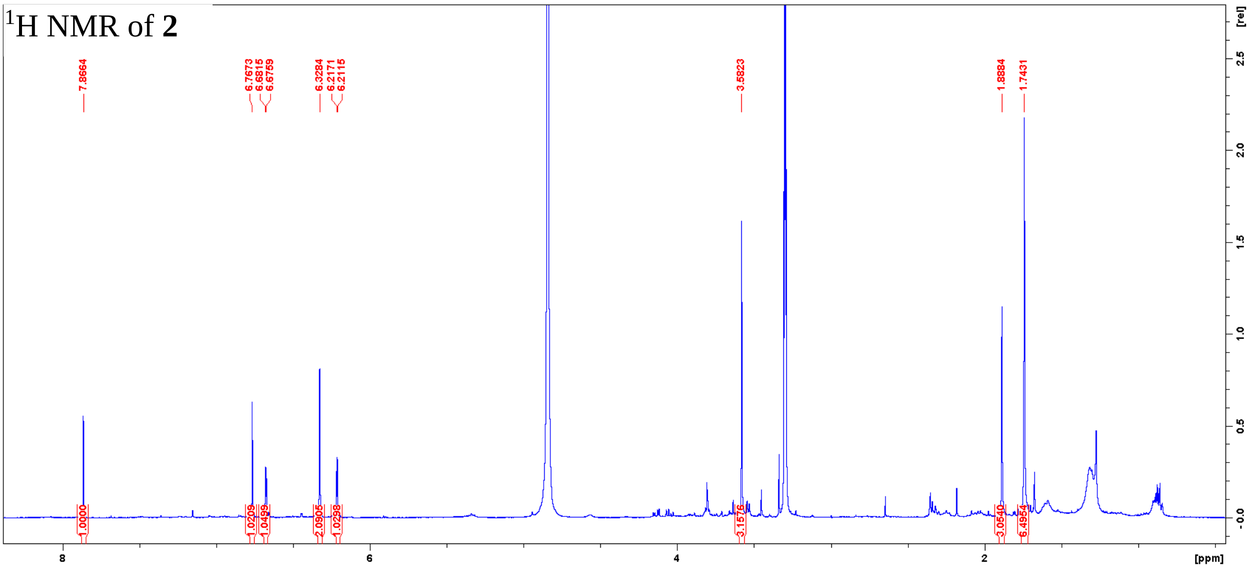

^{1}H NMR of **2**

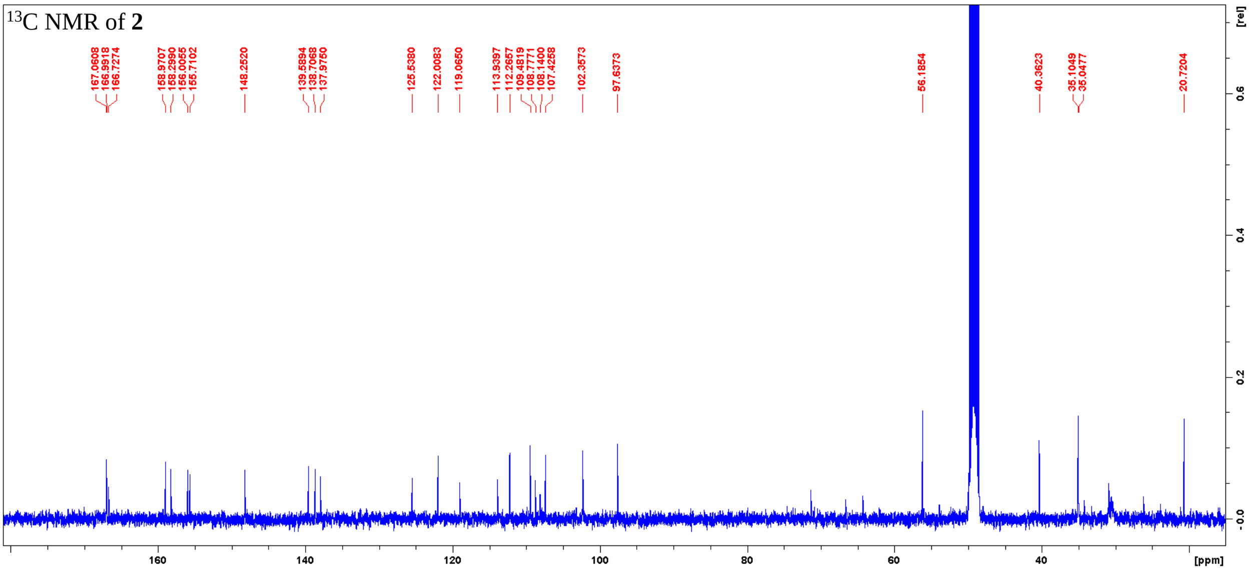

^{13}C NMR of **2**

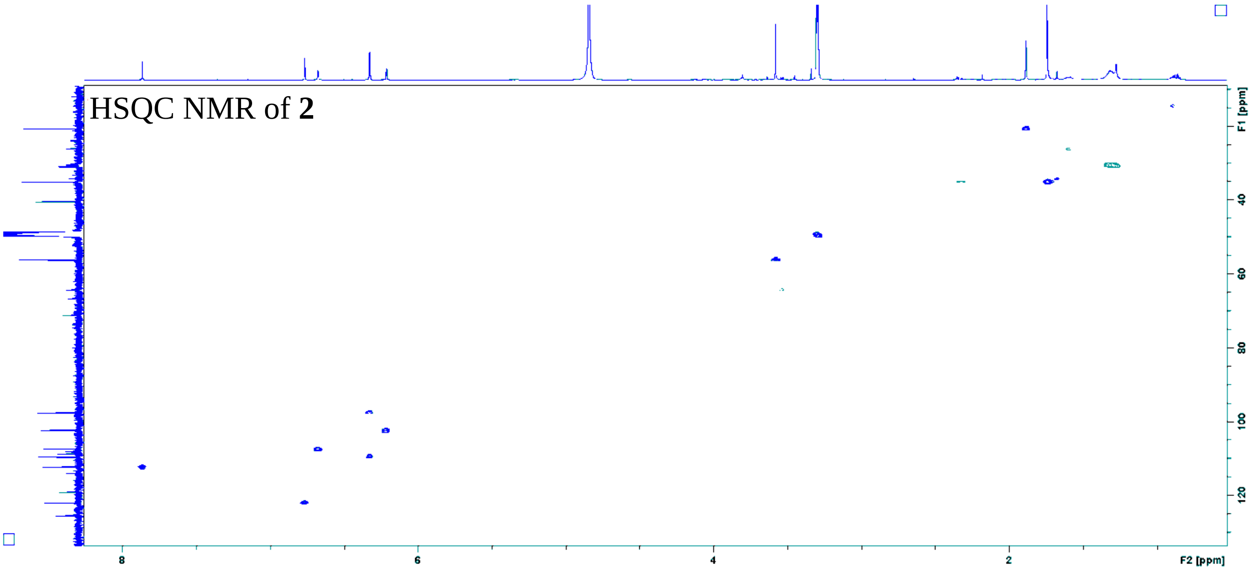
HSQC NMR of **2**
F1 [ppm]
40
60
80
100
120
8
6
4
2
F2 [ppm]

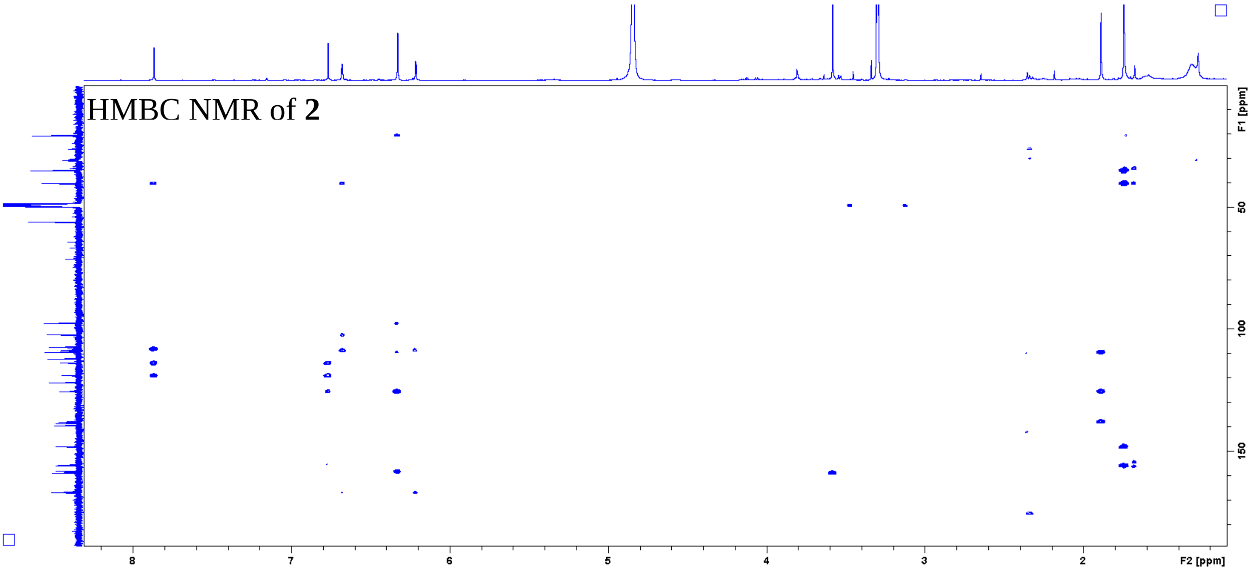
HMBC NMR of **2**
F1 [ppm]
50
100
150
8
7
6
5
4
3
2
F2 [ppm]

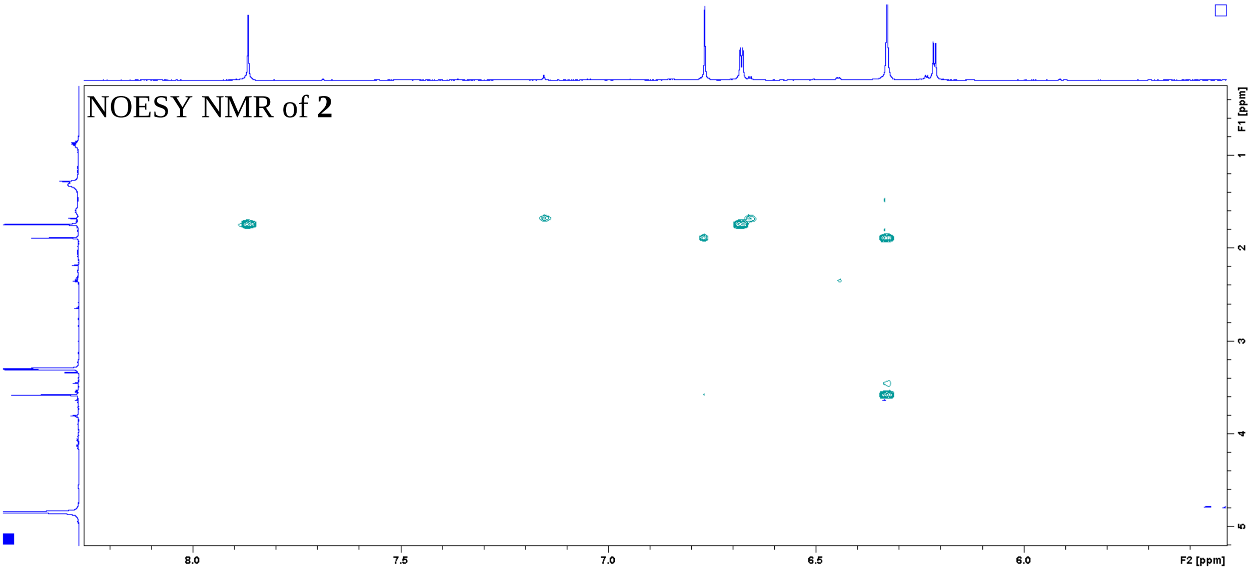
NOESY NMR of **2**
F1 [ppm]
1
2
3
4
5
8.0
7.5
7.0
6.5
6.0
F2 [ppm]

Fasamycin E (3)

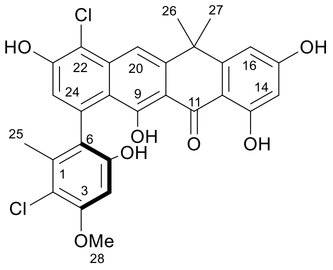

Molecular formula: $C_{28}H_{21}Cl_2O_7$

Isolated yield: 2 mg

UV (PDA): λ_{max} = 249, 289, 355 and 415 nm

Optical activity: $[\alpha]_D^{20}$ = +27.02

HRMS (ESI) *m/z*: calculated $[M + H]^+$ = 541.0815, observed $[M + H]^+$ = 541.0826, Δ = 2.03 ppm

NMR (CD_3OD; 400 MHz & 100 MHz): ^1H, ^{13}C, HSQC, HMBC, NOESY

Position	δ_C ppm	δ_H ppm (no. of protons, multiplicity, J in Hz)	HMBC	NOESY
1	135.6		25	
2	113.7		4, 25	
3	157.1		4, 28	
4	98.6	6.5 (1H, s)		28
5	153.9		4	
6	126.7		4, 24, 25	
7	not detected			
8	118.9		20, 24	
9	not detected			
10	108.23		20	
11	191.9			
12	108.7		14	
13	167.1		14	
14	102.4	6.2 (1H, d, 2.25)		
15	167.1			
16	107.5	6.7 (1H, d, 2.25)		26, 27
17	156.0		26, 27	

18	40.4		16, 20, 26, 27	
19	148.5		26, 27	
20	112.3	7.9 (1H, s)		26, 27
21	not detected			
22	114.3		20, 24	
23	not detected			
24	122.0	6.8 (1H, s)		25
25	18.4	2.0 (3H, s)		24
26	35.1	1.7 (3H, s)	27	16, 20
27	35.1	1.7 (3H, s)	26	16, 20
28	56.3	3.6 (3H, s)		4

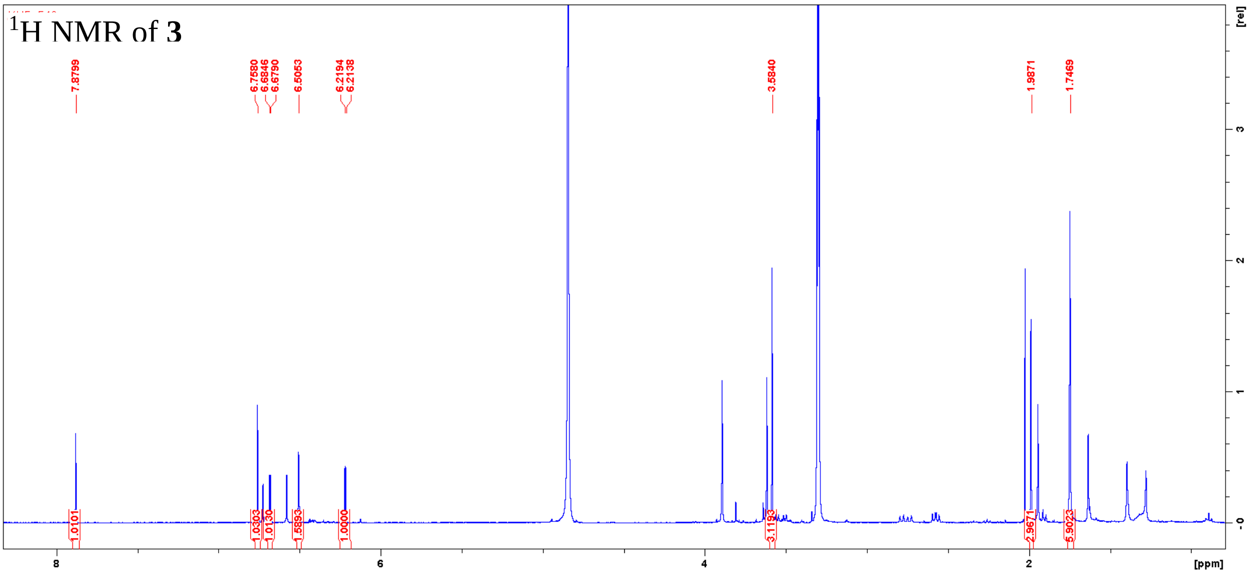

^{1}H NMR of **3**

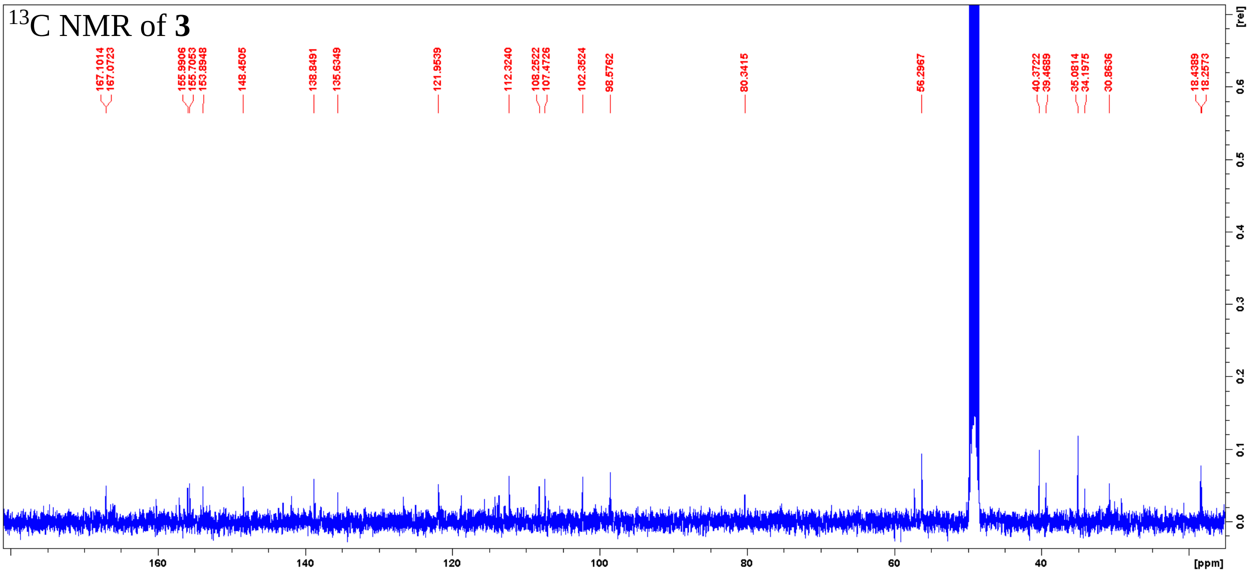
13C NMR of 3
167.1014
167.0723
155.9906
155.7063
153.8948
148.4505
138.8491
135.6349
121.9539
112.3240
108.2522
107.4726
102.3524
98.5762
80.3415
56.2967
40.3722
39.4689
35.0814
34.1975
30.8636
18.4389
18.2573
[ppm]
[rel]

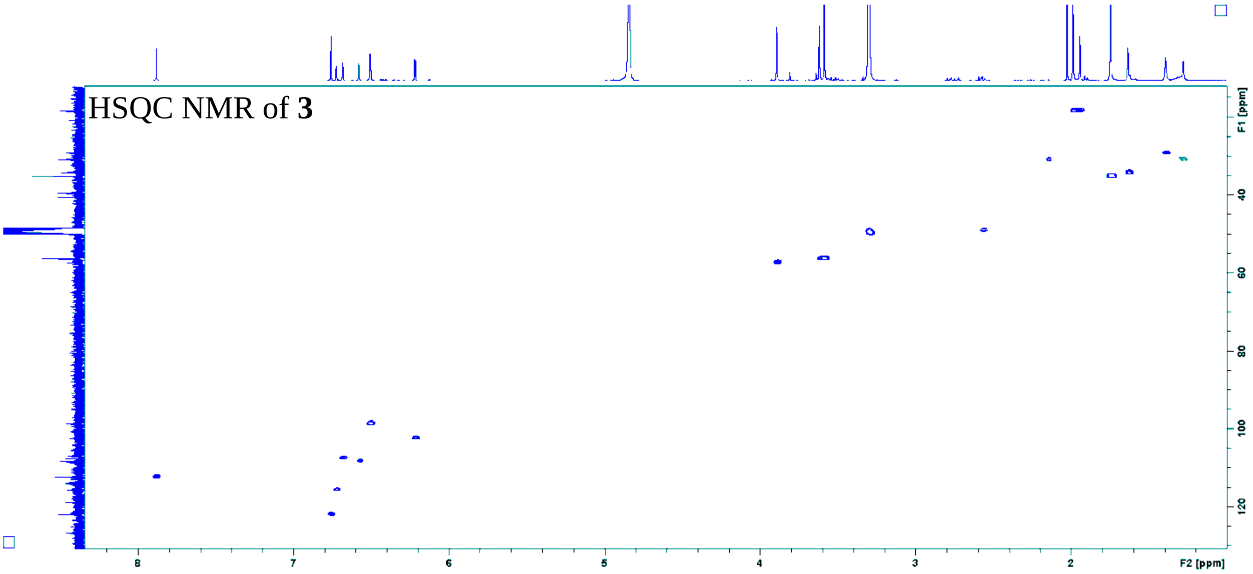
HSQC NMR of 3
F2 [ppm]
F1 [ppm]

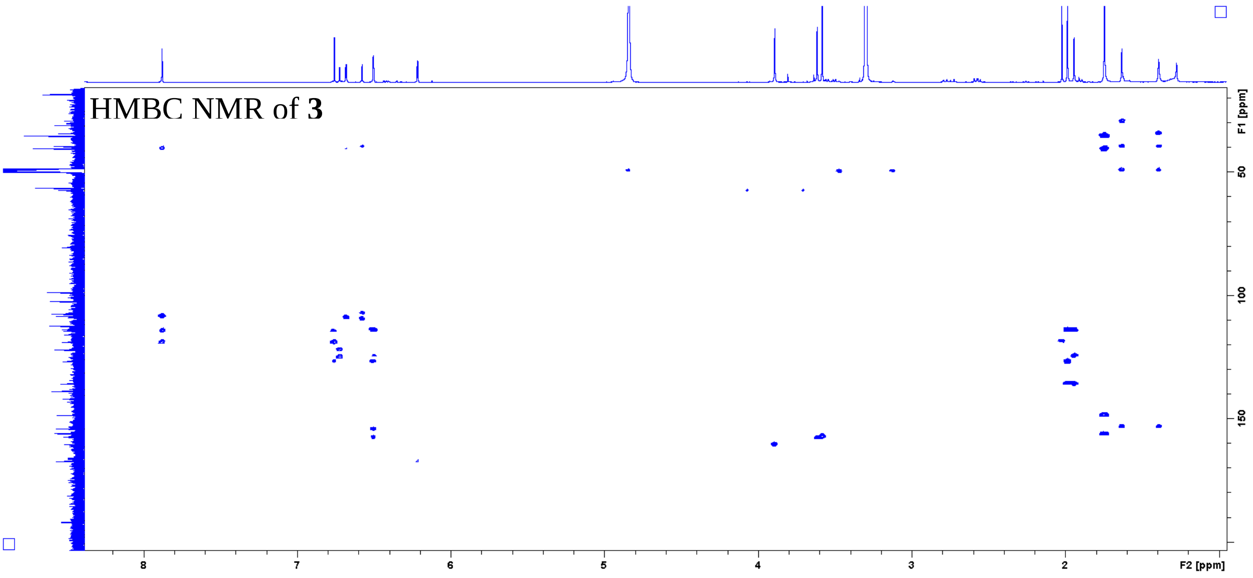
HMBC NMR of 3
F2 [ppm]
F1 [ppm]

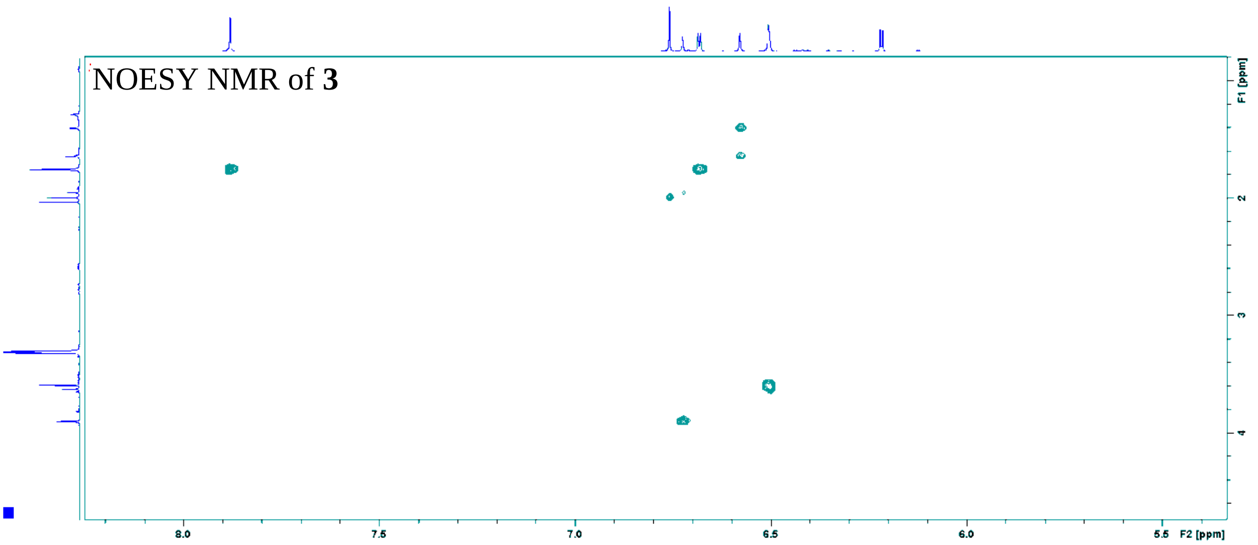

Formicamycin A (4)

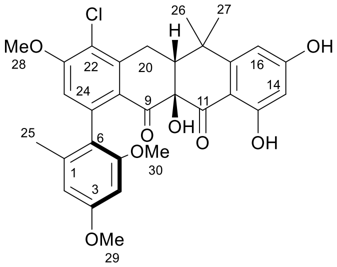

Molecular formula: $C_{30}H_{29}O_8Cl$

Isolated yield: 6 mg

UV (PDA): λ_{max} = 238 and 287 nm

Optical activity: $[\alpha]_D^{20}$ = +176.79

HRMS (ESI) *m/z*: calculated $[M + H]^+$ = 553.1624, observed $[M + H]^+$ = 553.1637, Δ = 2.35 ppm

NMR (CD_3OD; 400 MHz & 100 MHz): ^1H, ^{13}C, HSQC, HMBC, NOESY

Position	δ_C ppm	δ_H ppm (no. of protons, multiplicity, J in Hz)	HMBC	NOESY
1	138.1		25	
2	107.7	6.4 (1H, d, 2.36)	4, 25	25, 29
3	161.5		4, 29	
4	97.1	6.4 (1H, d, 2.36)	2	30
5	159.4		4, 30	

6	124.4		2, 24, 25	
7	142.4		24	
8	125.1		20, 24	
9	195.3			
10	80.2		19, 20	
11	198.7		19	
12	109.2		13, 16	
13	167.6		14	
14	101.9	6.1 (1H, d, 2.26)	16	
15	167.9		14, 16	
16	108.2	6.4 (1H, d, 2.26)	26, 27	
17	155.8		19, 26, 27	
18	39.6		16, 19, 26, 27	
19	49.0,	2.6 (1H, dd, 9.25, 6.60)	20, 26, 27	20, 26,27
20	30.2	3.5 (1H, dd, 19.33, 9.25);<br>2.8 (1H, dd, 19.33, 6.60)	19	
21	143.1		20	
22	121.6		20, 24	
23	160.2		24, 28	
24	115.6	6.7 (1H, s)		25, 28
25	20.7	1.9 (3H, s)	2	2, 24
26	34.2	1.6 (3H, s)	27	16, 19, 27
27	29.2	1.4 (3H, s)	26	16, 19, 20, 26
28	57.2	3.9 (3H, s)		24
29	55.9	3.8 (3H, s)		2
30	56.3	3.7 (3H, s)		4

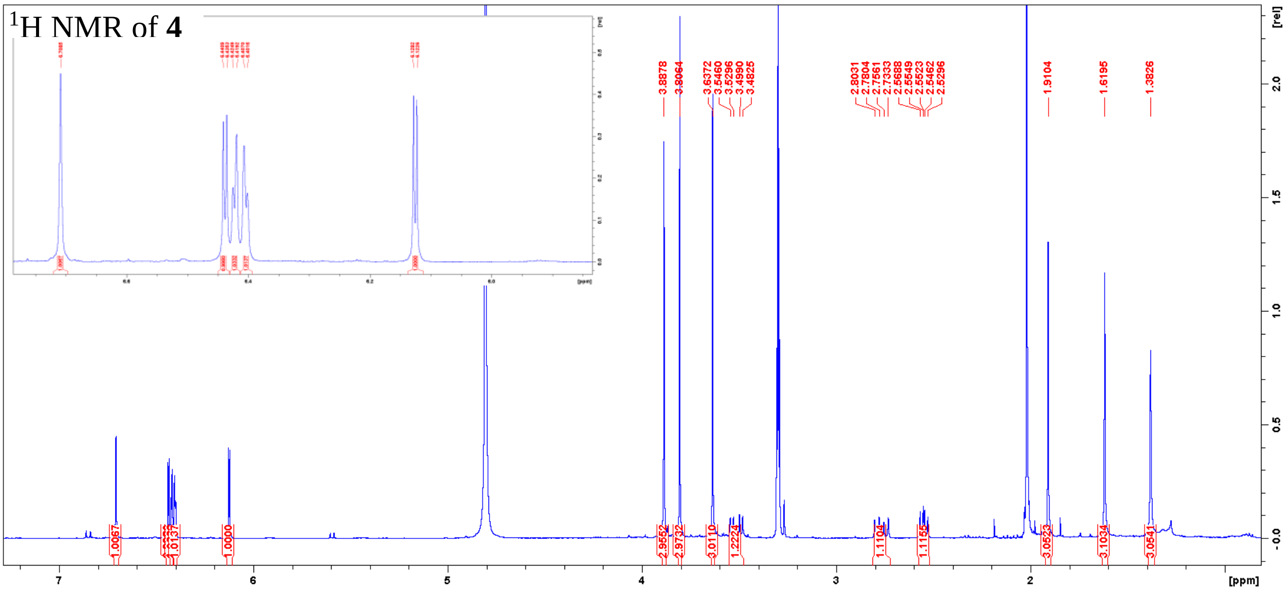
1H NMR of 4
3.8878
3.8064
3.6372
3.5460
3.5296
3.4990
3.4825
2.8031
2.7804
2.7561
2.7333
2.5688
2.5549
2.5523
2.5462
2.5296
1.9104
1.6195
1.3826
[ppm]
[rel]

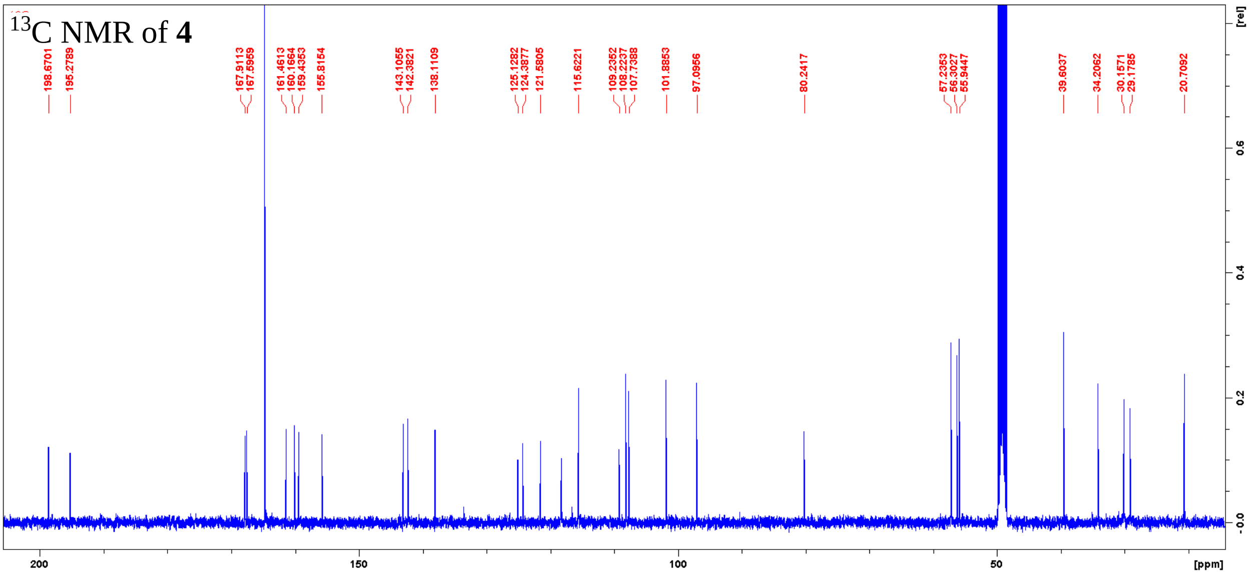
13C NMR of 4
198.6701
195.2789
167.9113
167.5959
161.4613
160.1664
159.4353
155.8154
143.1055
142.3821
138.1109
125.1292
124.3877
121.5805
115.6221
109.2352
108.2237
107.7388
101.8853
97.0956
80.2417
57.2353
56.3027
55.9447
39.6037
34.2062
30.1571
29.1785
20.7092
[ppm]
[rel]

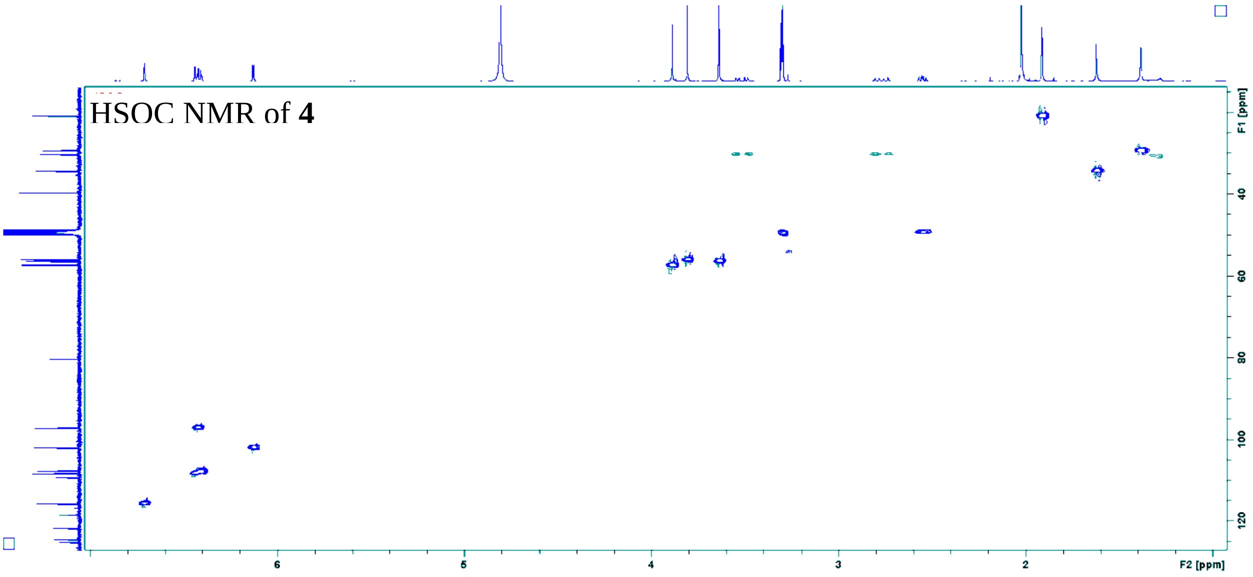
HSQC NMR of 4
F2 [ppm]
F1 [ppm]

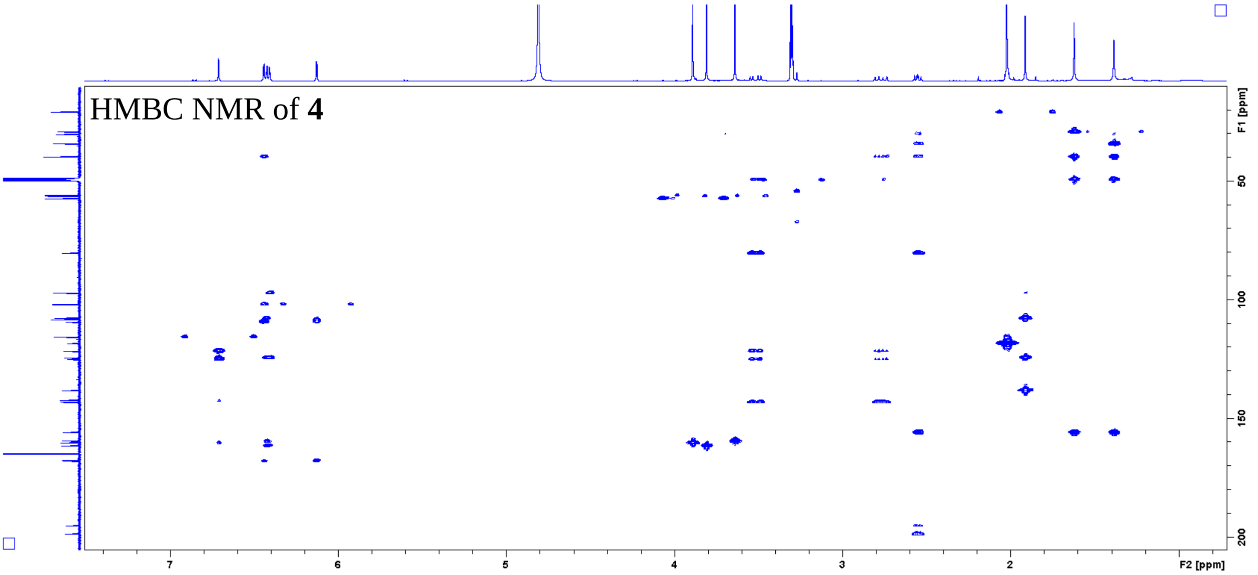

HMBC NMR of **4**

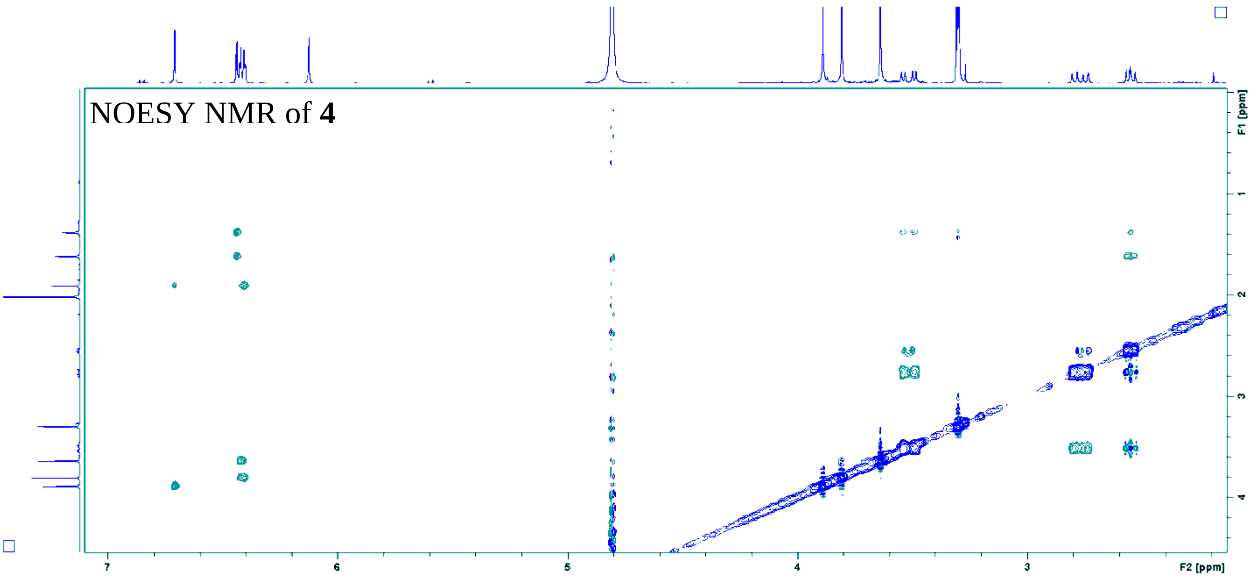

NOESY NMR of **4**

Formicamycin B (5)

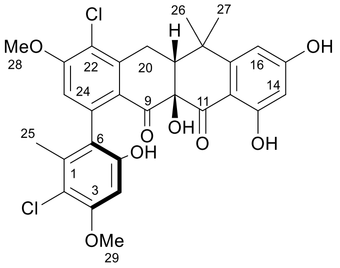

Molecular formula: $C_{29}H_{26}O_8Cl_2$

Isolated yield: 18 mg

UV (PDA): λ_{max} = 233 and 289 nm

Optical activity: $[\alpha]_D^{20}$ = +218.32

HRMS (ESI) *m/z*: calculated $[M + H]^+$ = 573.1077, observed $[M + H]^+$ = 573.1072, Δ = -0.96 ppm

NMR (CD_3OD; 400 MHz & 100 MHz): ^1H, ^{13}C, COSY, HSQC, HMBC, NOESY

position	δ_C ppm	δ_H ppm (no. of protons, multiplicity, J in Hz)	^1H - ^1H COSY	HMBC	NOESY
1	135.7			25	
2	113.9			4, 25	
3	157.6			4, 29	
4	98.7	6.5 (1H, s)			29
5	154.2			4	
6	124.3			4, 24, 25	
7	141.9			24	
8	125.1			24	
9	195.3			19	
10	80.3			19, 20	
11	198.6			19	
12	109.2			14, 16	
13	167.6			14	
14	101.9	6.1 (1H, d, 2.30)	16	16	
15	168.0		14	14, 16	
16	108.2	6.5 (1H, d, 2.30)		14	26, 27
17	155.7			19, 26, 27	
18	39.6			16, 19, 20, 26, 27	
19	49.1	2.6 (1H, dd, 9.18, 6.66)	20	20, 26, 27	20, 26, 27
20	30.2	2.8 (1H, dd, 18.94 , 9.18); 3.5 (1H, dd, 18.94, 6.66)	19	19	26, 27
21	143.1			19, 20	
22	121.8			20, 24	
23	160.2			24, 28	
24	115.6	6.7 (1H, s)			25, 28
25	18.3	1.9 (3H, s)			24
26	29.2	1.4 (3H, s)		27	16, 19, 20,

				27
27	34.2	1.6 (3H, s)	19, 26	16, 19, 20, 26
28	57.3	3.9 (3H, s)		24
29	56.3	3.6 (3H, s)		4

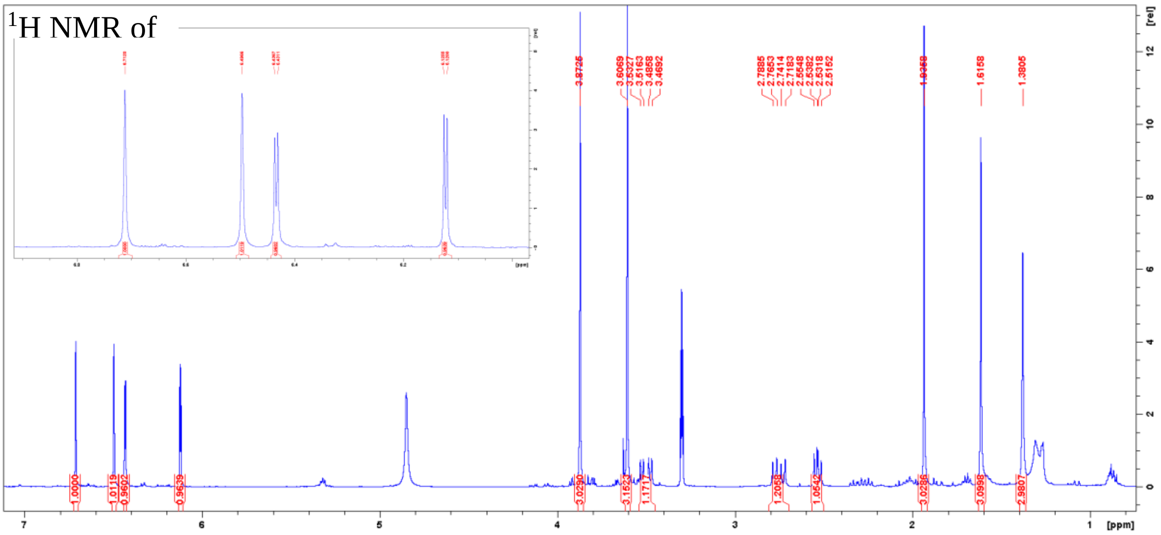

^{1}H NMR of

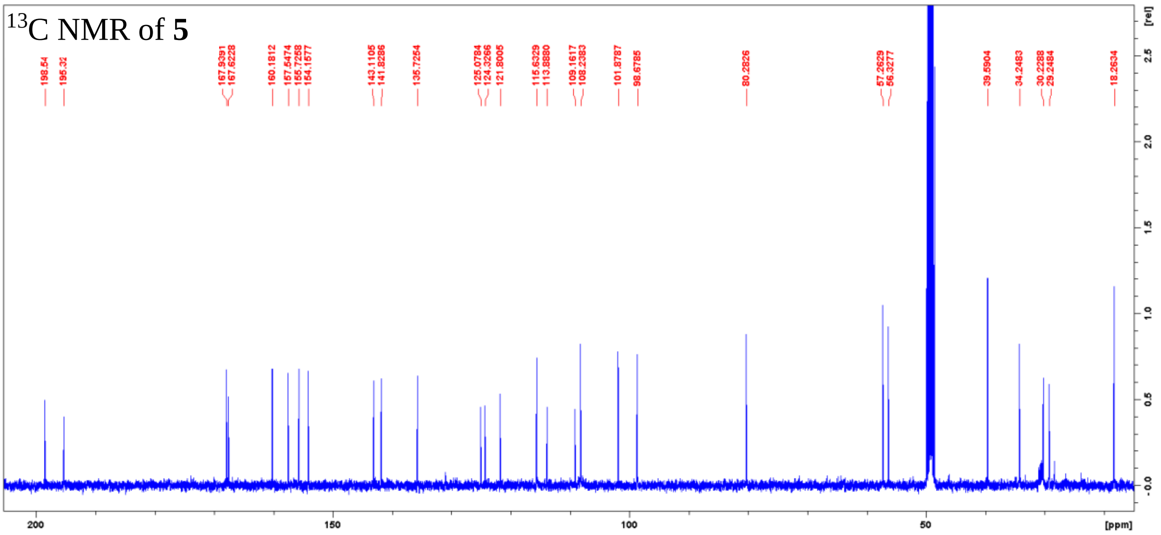

^{13}C NMR of **5**

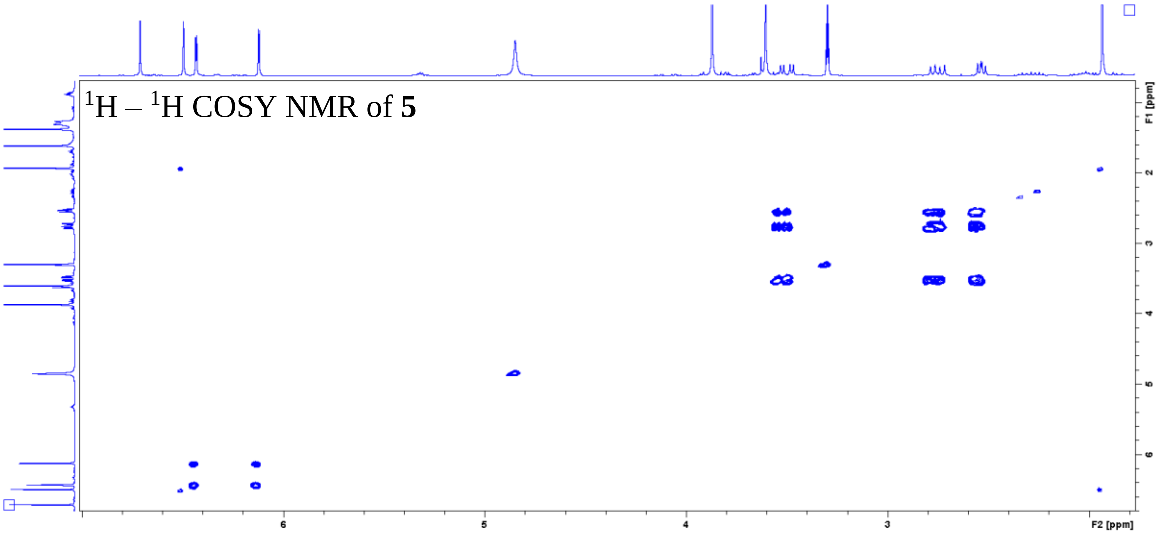
1H – 1H COSY NMR of 5
F1 [ppm]
F2 [ppm]
6
5
4
3
2

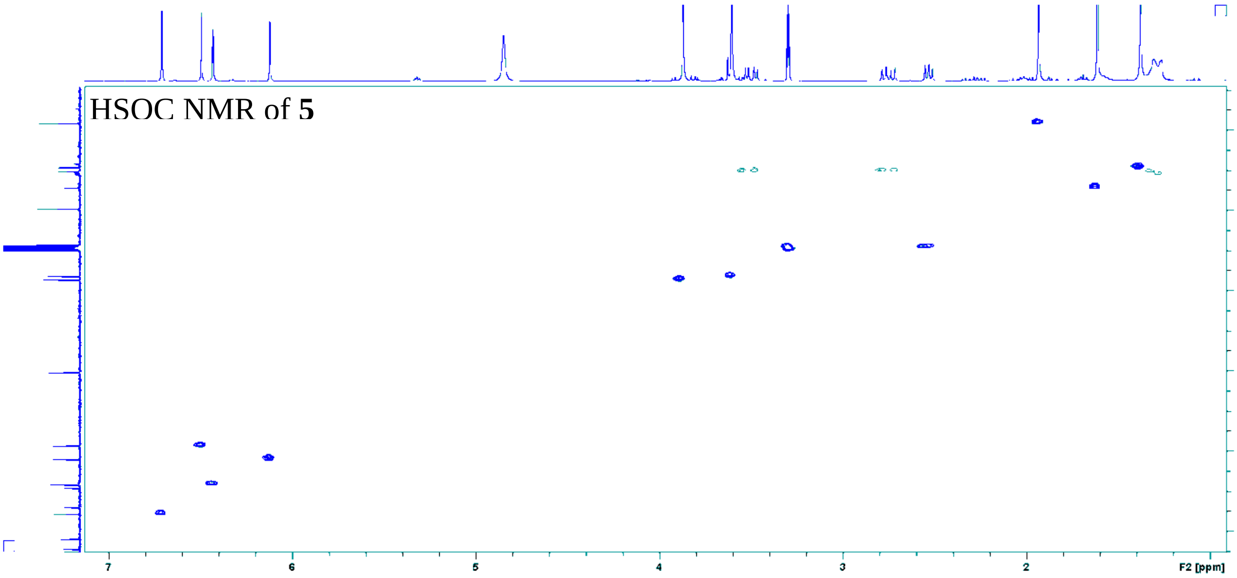
HSQC NMR of 5
F1 [ppm]
F2 [ppm]
7
6
5
4
3
2
40
60
80
100
120

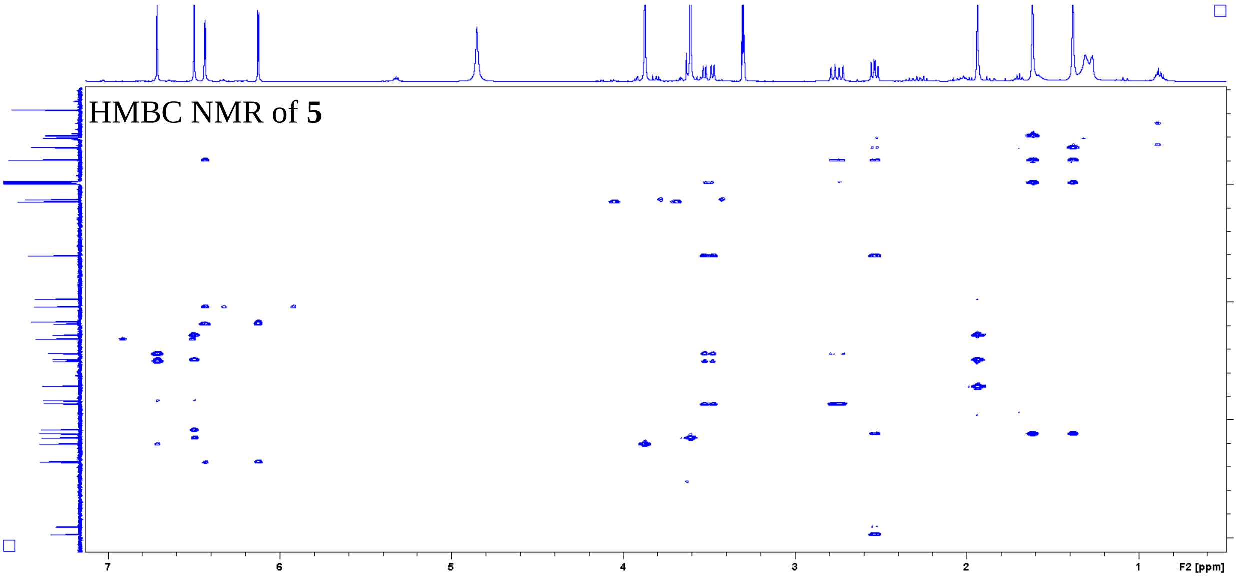
HMBC NMR of 5
F1 [ppm]
F2 [ppm]
7
6
5
4
3
2
1
50
100
150
200

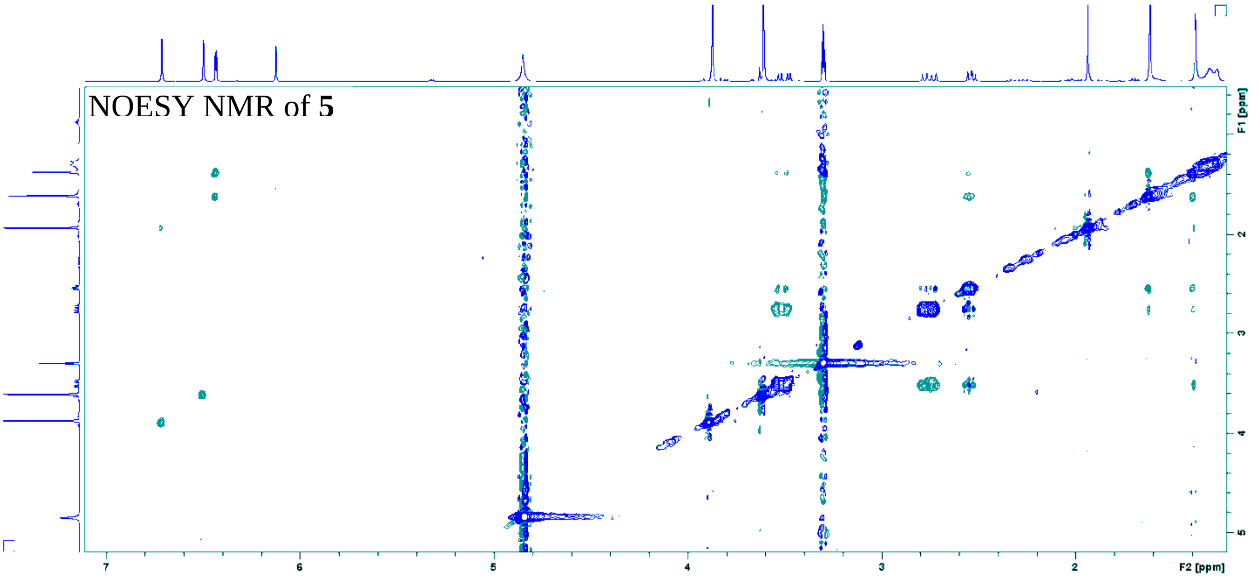

NMR analysis of (5) in non-protic solvents

The stereochemistry of **5** was further investigated using NMR analysis in non-protic solvents. **5** was first dissolved in d_6-DMSO and submitted for 1D (^{1}H and ^{13}C) and 2D NMR (HSQC, HMBC and NOESY) analysis. The resulting spectra could be readily assigned and, critically, a previously unobserved proton singlet at δ_H 6.26 ppm was observed. HMBC correlations between this proton and C9, C10, and C19 identified it as the 10-hydroxyl proton. However, the NOSEY spectrum obtained was of poor quality, possibly due to the high viscosity of DMSO. To overcome this d_3-acetonitrile (~30%) was added to the d_6-DMSO sample which resulted is much improved ^{1}H and NOESY data in which correlations were observed between the 10-hydroxyl proton and H19 and H27, in addition to the previously observed correlations between H19 and H26/27. Additional key NOESY correlations between H19 to H20b, H20b to H26, and H20a to H20b were also observed. These data are again consistent with the (*10R,19R*) isomer, but not (*10S,19R*) isomer. Analysis of the NOESY data for all compounds **4-15** was consistent with that for **5** indicating a common absolute stereochemistry for the formicamycins.

NOTE: weak NOESY correlations between H20a and H26/27 were also observed in the various NOESY data for **5**. Given the very strong correlations observed between H19 and H26/27, and the close chemical shifts for H19 (2.6 ppm) and H20a (2.8 ppm), we reasoned that the resolution of our experiment was such that irradiation of each of these signals occurs with some overlap leading to 'falsely' observed

correlations. Analysis of the NOESY spectra for all compounds **4-15** showed this phenomena other than **11**, for which the chemical shifts of H19 (2.4 ppm) and H20a (3.0 ppm) were much better resolved – no false positive signals were observed in this spectra consistent with our hypothesis.

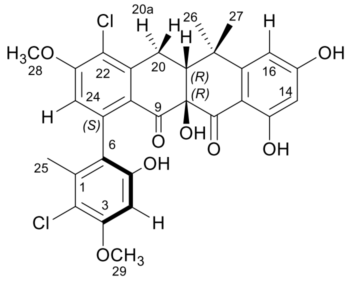

NMR (in d_6-DMSO; 400 MHz & 100 MHz) of **5**

position	δ_C ppm	δ_H ppm (no. of protons, multiplicity, J in Hz)	HMBC
1	133.7		25
2	111.6		4, 25
3	155.6		4, 29
4	97.5	6.5 (1H, s)	
5	152.7		4
6	122.0		4, 24, 25
7	139.6		24
8	123.6		24
9	193.2		10, 19
10	78.7	6.3 (1H, s)	10, 19, 20
11	197.5		19
12	107.2		14, 16
13	165.2		14
14	100.5	6.1 (1H, d, 2.16)	16
15	166.2		14, 16
16	107.1	6.4 (1H, d, 2.16)	14
17	154.1		19, 26, 27
18	37.9		16, 19, 20, 26, 27
19	46.5	2.6 (1H, m)	10, 20, 26, 27
20	28.5	20a, 2.7 (m);<br>20b, (NA)	19
21	141.1		19, 20
22	119.4		20, 24
23	157.8		24, 28
24	114.4	6.8 (1H, s)	
25	17.6	1.9 (3H, s)	

26	28.2	1.3 (3H, s)	27
27	33.2	1.5 (3H, s)	19, 26
28	56.8	3.9 (3H, s)	
29	55.5	3.6 (3H, s)	

NMR (d_6-DMSO/d_3-acetonitrile (70:30); 400 MHz & 100 MHz) of **5**

position	δ_H ppm (no. of protons, multiplicity, J in Hz)	NOESY
4	6.6 (1H, s)	29
10	6.1 (1H, s)	19, 27
14	6.2 (1H, d, 2.21)	
16	6.5 (1H, d, 2.21)	26, 27
19	2.6 (1H, dd, 9.38, 6.56)	10, 20b, 26, 27
20a	2.7 (1H, dd, 18.94, 9.38)	20b
20b	3.4 (1H, dd, 18.94, 6.56)	20a, 19, 26
24	6.8 (1H, s)	25, 28
25	1.9 (3H, s)	24
26	1.3 (3H, s)	16, 19, 20b, 27
27	1.6 (3H, s)	10, 16, 19, 26
28	3.9 (3H, s)	24
29	3.6 (3H, s)	4

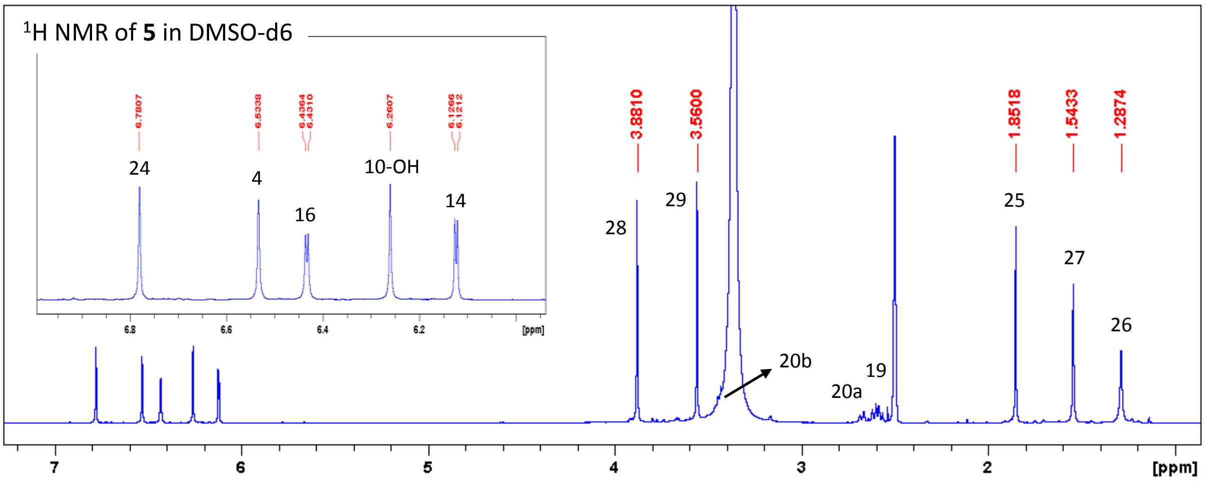

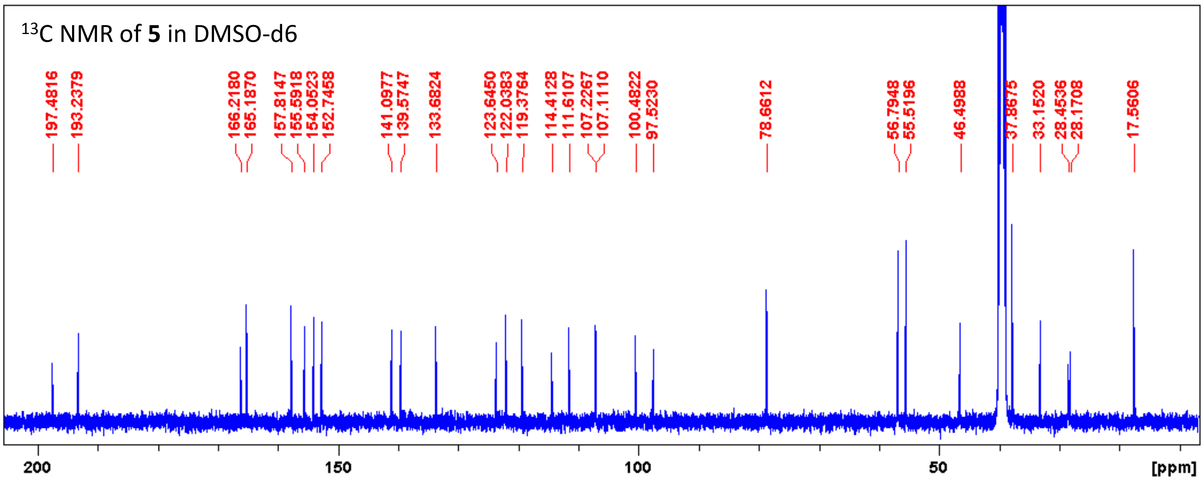
13C NMR of 5 in DMSO-d6
197.4816
193.2379
166.2180
165.1870
157.8147
155.5918
154.0523
152.7458
141.0977
139.5747
133.6824
123.6450
122.0383
119.3764
114.4128
111.6107
107.2267
107.1110
100.4822
97.5230
78.6612
56.7948
55.5196
46.4988
37.8675
33.1520
28.4536
28.1708
17.5606
[ppm]

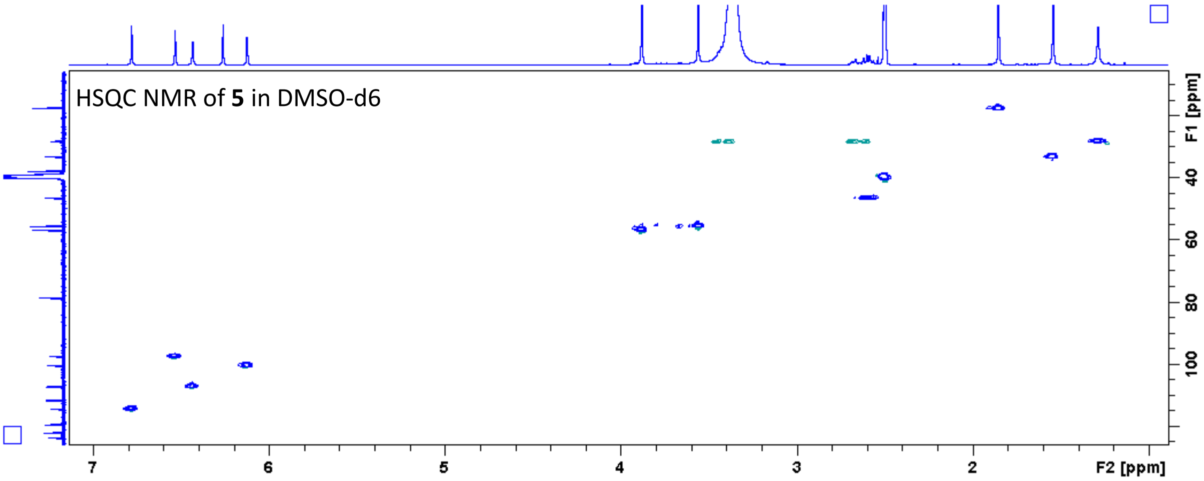
HSQC NMR of 5 in DMSO-d6
F1 [ppm]
F2 [ppm]

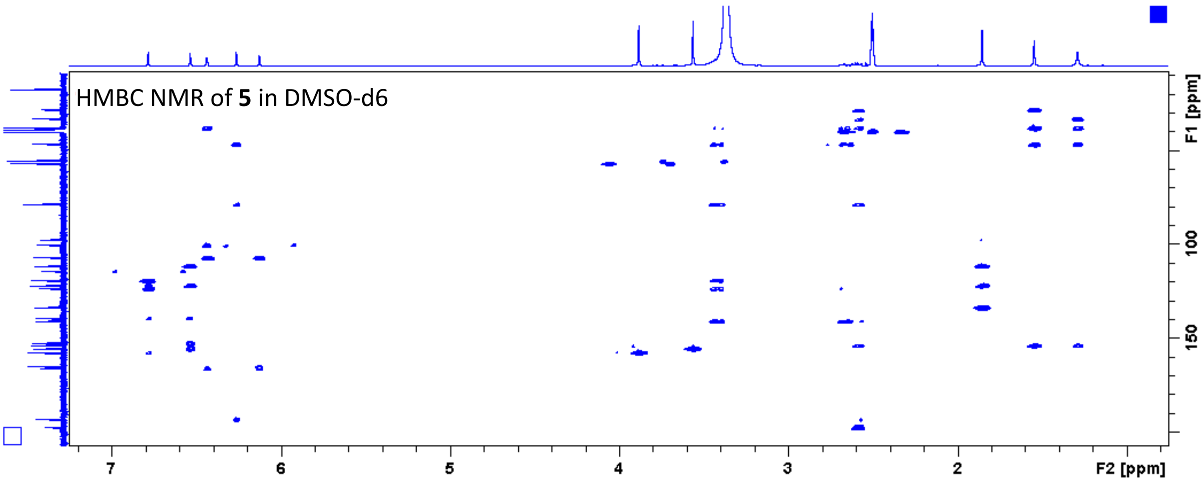
HMBC NMR of 5 in DMSO-d6
F1 [ppm]
F2 [ppm]

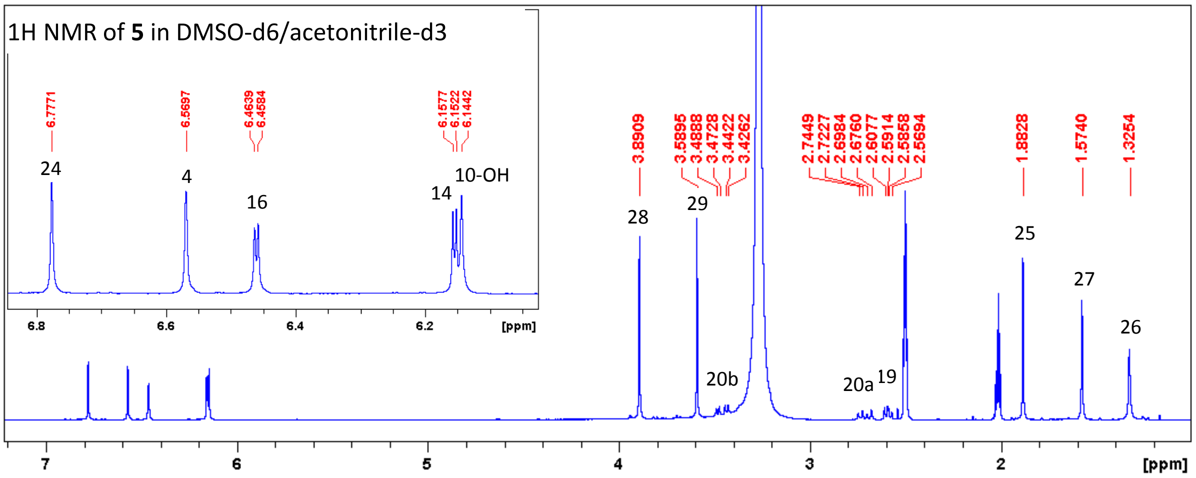

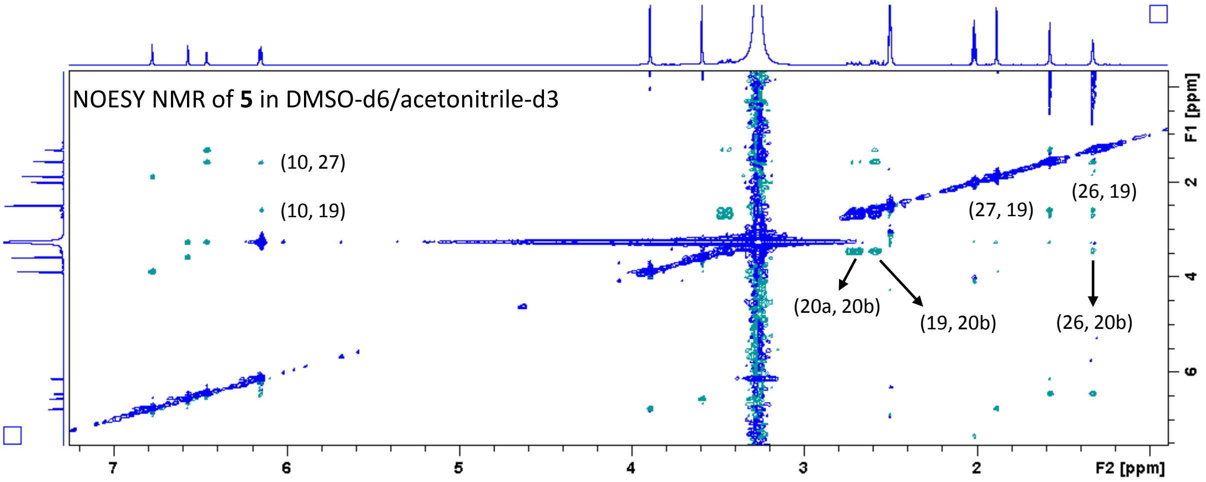

Formicamycin C (6)

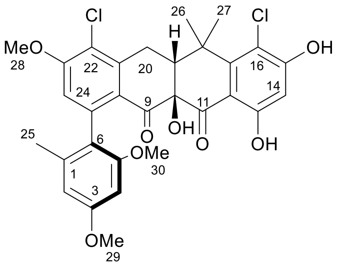

Molecular formula: $C_{30}H_{28}O_8Cl_2$

Isolated yield: 12 mg

UV (PDA): λ_{max} = 227 and 286 nm

Optical activity: $[\alpha]_D^{20}$ = +242.52

HRMS (ESI) *m/z*: calculated $[M + H]^+$ = 587.1234, observed $[M + H]^+$ = 587.1252, Δ = 3.07 ppm

NMR (CD_3OD; 400 MHz & 100 MHz): ^1H, ^{13}C, HSQC, HMBC, NOESY

position	δ_C ppm	δ_H ppm (no. of protons, multiplicity, J in Hz)	HMBC	NOESY
1	138.1		25	
2	107.7	6.4 (1H, d, 2.25)	4, 25	25, 29
3	161.5		4, 29	
4	97.1	6.4 (1H, d, 2.25)		30
5	159.4		4, 30	
6	124.4		2, 4, 24, 25	
7	142.4			
8	124.9		20, 24	
9	195.0		19	
10	80.3		19, 20	
11	199.9		19	
12	110.6		14	
13	164.0		14	
14	103.2	6.4 (1H, s)		
15	166.1		14	
16	114.9		14	
17	149.6		19, 26, 27	
18	41.4		19, 20, 26, 27	
19	51.5	2.5 (1H, dd, 6.20, 10.16)	20, 26, 27	20, 26, 27
20	30.3	2.8 (1H, dd, 10.16, 18.08); 3.6 (1H, m)	19	19
21	143.0		20	
22	121.8		20, 24	
23	160.2		24, 28	
24	115.7	6.7 (3H, s)		25, 28
25	20.7	1.9 (3H, s)	2	2, 24
26	27.4	1.7 (3H, s)	27	19, 20, 27
27	29.3	1.9 (3H, s)	19, 26	19, 26
28	57.3	3.9 (3H, s)		24
29	55.9	3.8 (3H, s)		2
30	56.3	3.6 (3H, s)		4

^{1}H NMR of **6**

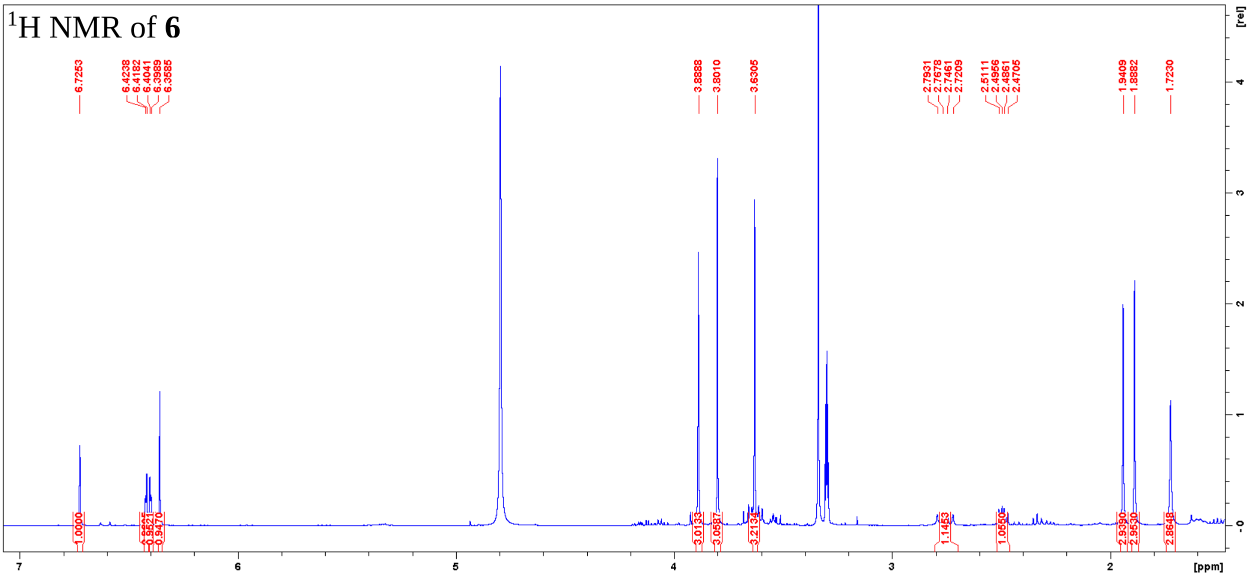

^{13}C NMR of **6**

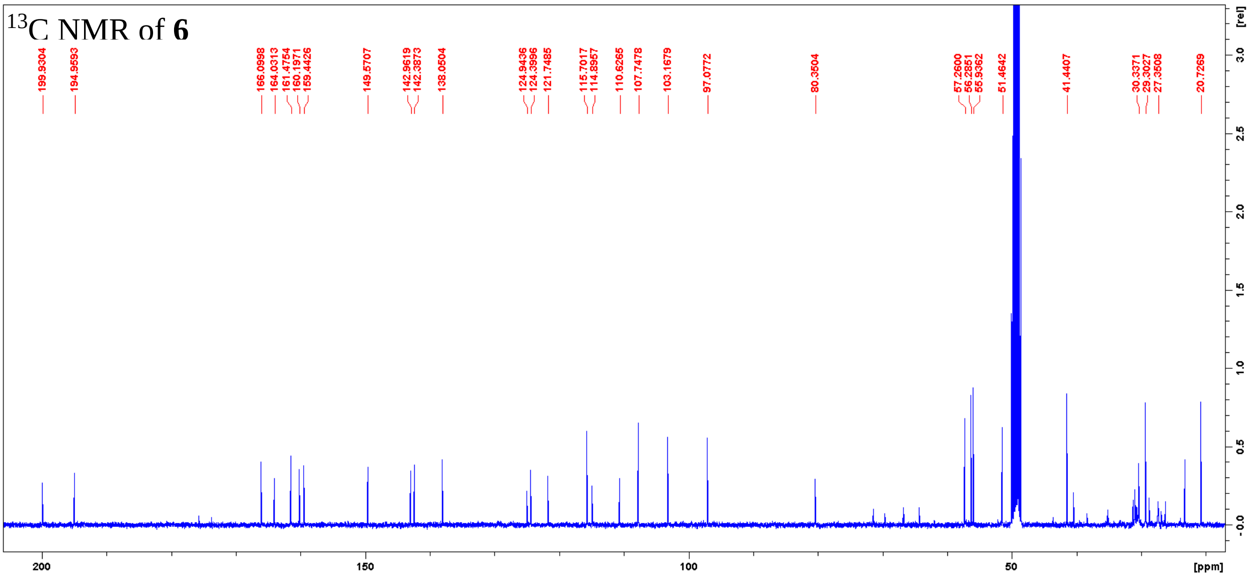

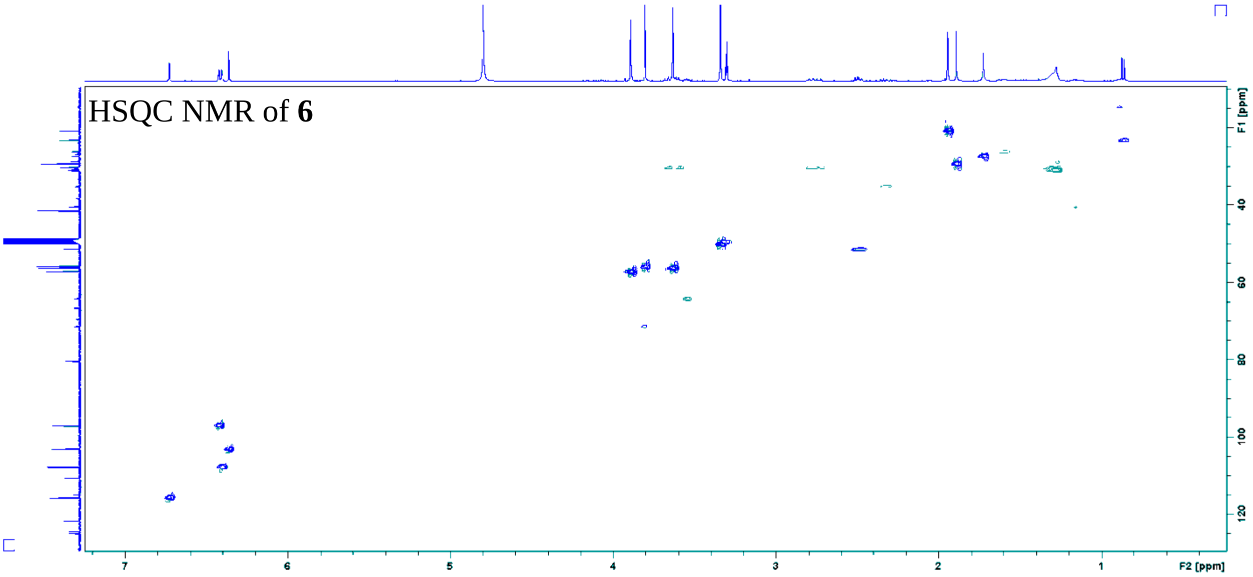
HSQC NMR of **6**
F1 [ppm]
F2 [ppm]

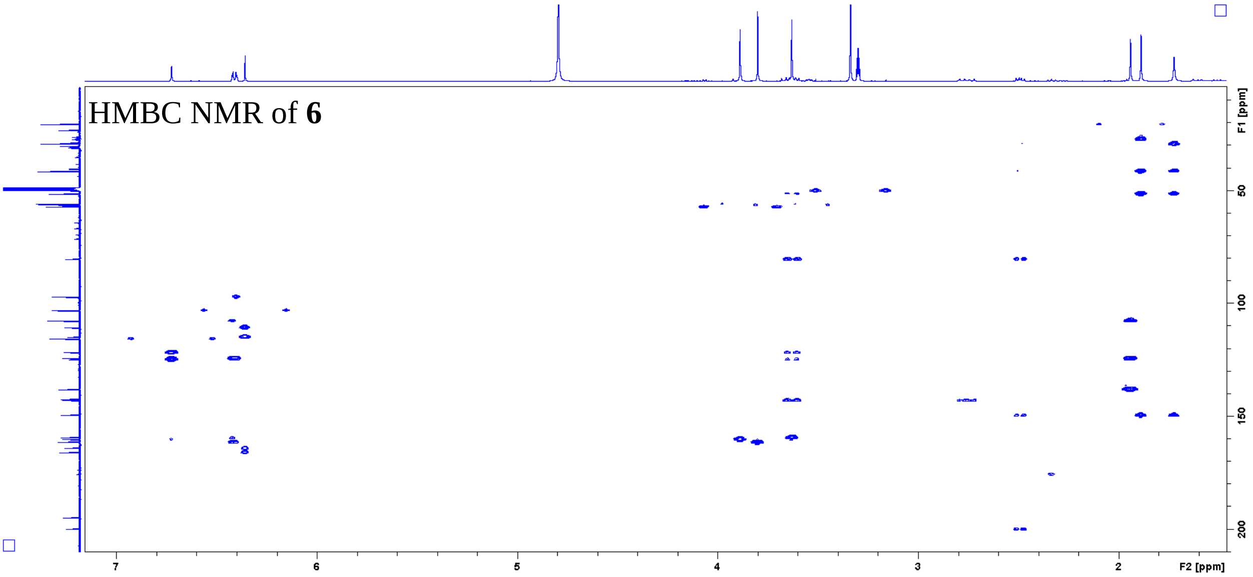
HMBC NMR of **6**
F1 [ppm]
F2 [ppm]

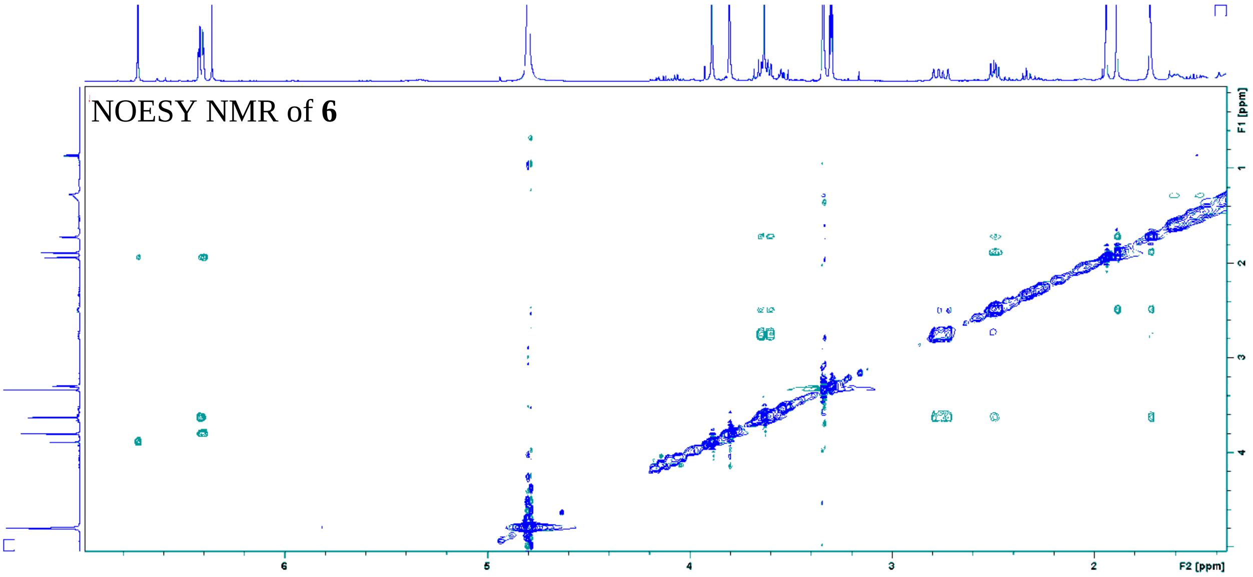
NOESY NMR of **6**
F1 [ppm]
F2 [ppm]

Formicamycin D (7)

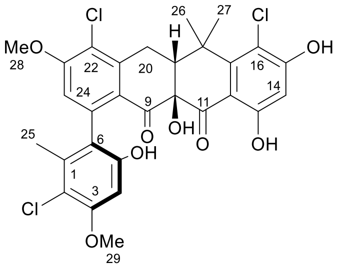

Molecular formula: $C_{29}H_{25}O_8Cl_3$

Isolated yield: 2 mg

UV (PDA): λ_{max} = 232 and 288 nm

Optical activity: $[\alpha]_D^{20}$ = +162.21

HRMS (ESI) *m/z*: calculated $[M + H]^+$ = 607.0688, observed $[M + H]^+$ = 607.0703, Δ = 2.47 ppm

NMR (CD_3OD; 400 MHz & 100 MHz): ^1H, ^{13}C, HSQC, HMBC, NOESY

position	δ_C ppm	δ_H ppm (no. of protons, multiplicity, J in Hz)	HMBC	NOESY
1	135.7		25	
2	114.0		4, 25	
3	157.6		4, 29	
4	98.8	6.5 (1H, s)		29
5	154.2		4	
6	124.4		4, 25	
7	141.8			
8	124.9		20, 24	
9	195.1			
10	80.4		20	
11	199.7			
12	115.1		14	
13	164.5		14	
14	103.2	6.4 (1H, s)		
15	166.2		14	
16	110.5		14	
17	149.5		26, 27	
18	41.5		26, 27	

19	51.5	2.5 (1H, dd, 9.96, 6.14)	20, 26, 27	20, 26, 27
20	30.4	2.8 (1H, dd, 18.92, 9.96); 3.6 (1H, m)		19, 27
21	143.0		20	
22	122.0		20, 24	
23	160.3		28	
24	115.7	6.7 (1H, s)		25, 28
25	18.2	2.0 (3H, s)		24
26	27.5	1.7 (3H, s)	26	19, 20
27	29.3	1.9 (3H, s)	27	19
28	57.3	3.9 (3H, s)		24
29	56.4	3.6 (3H, s)		4

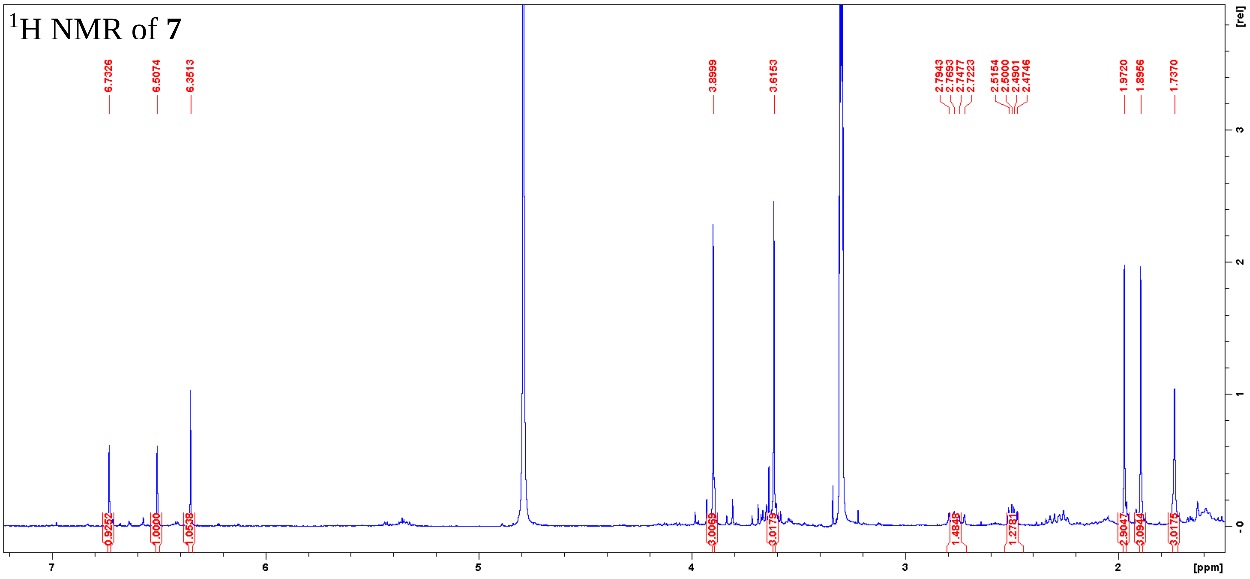
^{1}H NMR of **7**

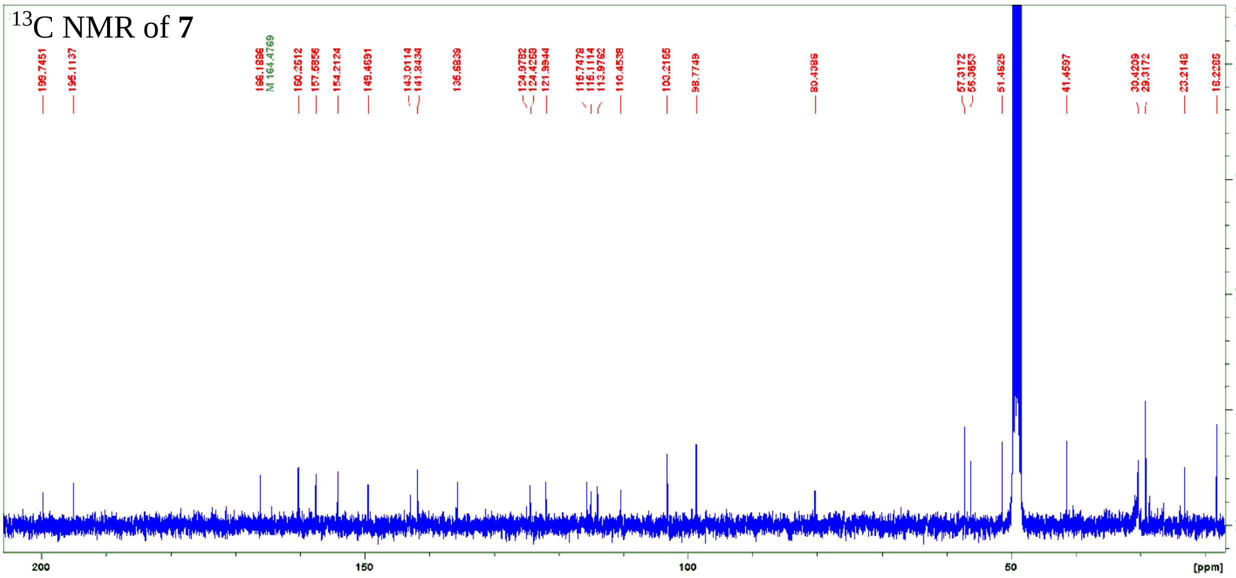
13C NMR of 7
199.7451
195.1137
166.1886
M 164.4769
160.2612
157.5856
154.2124
149.4691
143.0114
141.3434
135.6839
124.9782
124.4258
121.9944
115.7479
115.1114
113.9762
110.4538
103.2165
98.7749
80.4386
57.3172
56.3653
51.4625
41.4597
30.4209
29.3172
23.2148
18.2285
[ppm]
[rel]

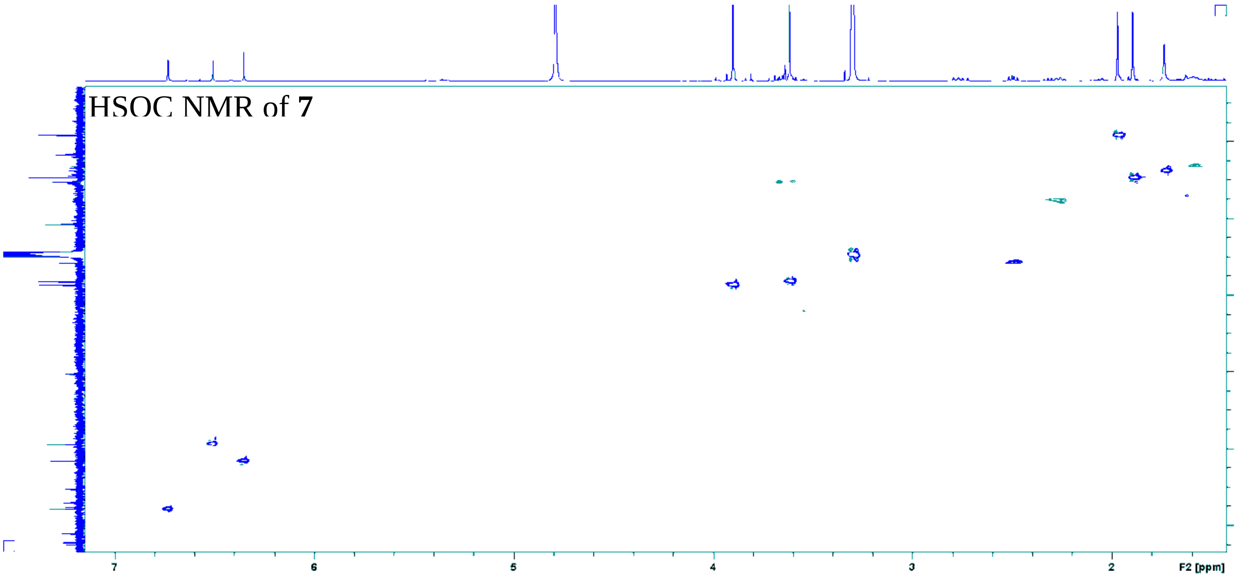
HSQC NMR of 7
F2 [ppm]
F1 [ppm]

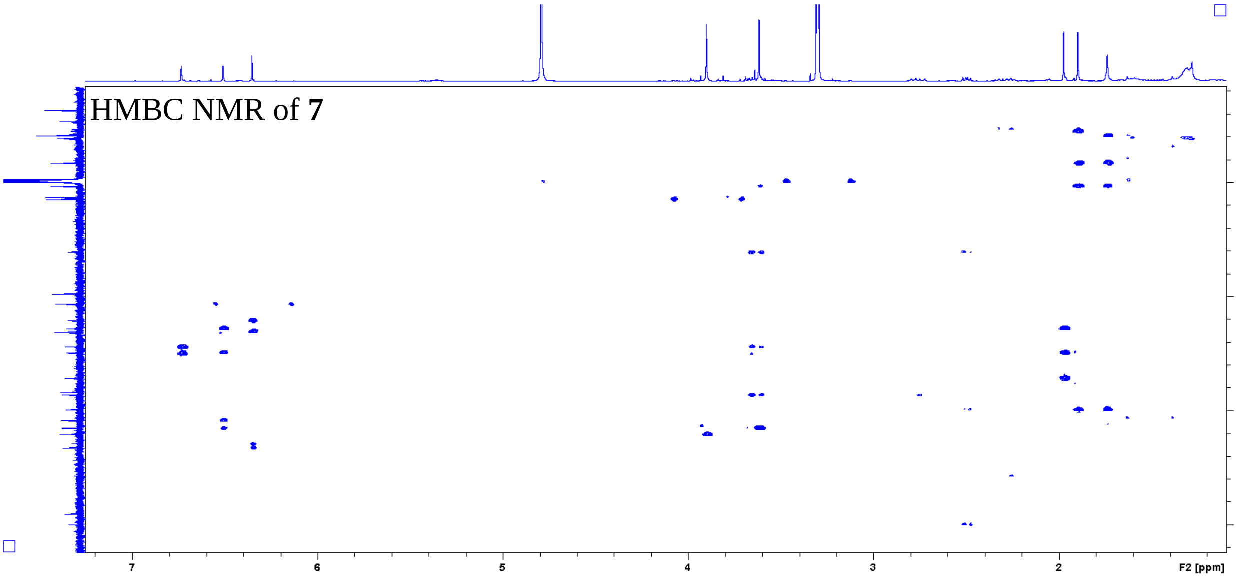
HMBC NMR of 7
F2 [ppm]
F1 [ppm]

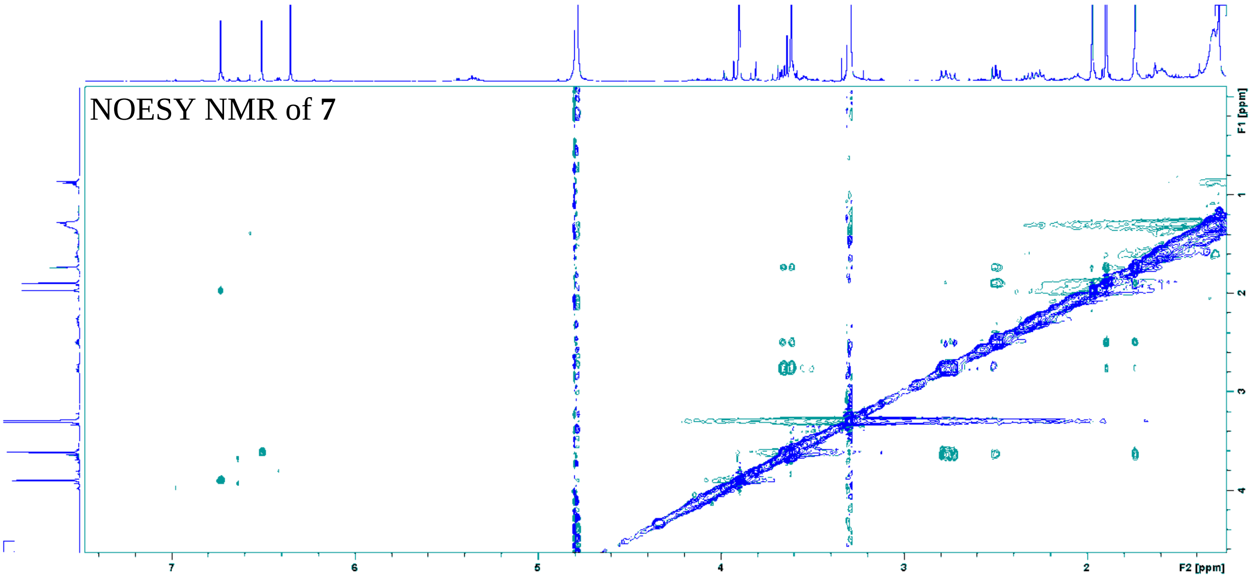

Formicamycin E (8)

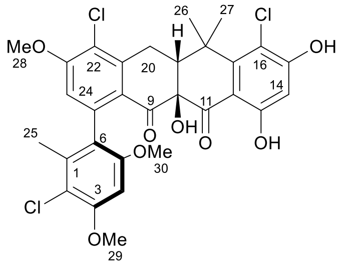

Molecular formula: $C_{30}H_{27}O_8Cl_3$

Isolated yield: 18 mg

UV (PDA): λ_{max} = 233 and 288 nm

Optical activity: $[\alpha]_D^{20}$ = +255.38

HRMS (ESI) *m/z*: calculated $[M + H]^+$ = 621.0844, observed $[M + H]^+$ = 621.0830, Δ = -2.25 ppm

NMR (CD_3OD; 400 MHz & 100 MHz): ^1H, ^{13}C, HSQC, HMBC, NOESY

Position	δ_C ppm	δ_H ppm (no. of protons, multiplicity, J in Hz)	HMBC	NOESY
1	136.1		25	
2	115.6		4, 25	
3	157.6		4, 29	
4	95.9	6.6 (1H, s)	25	29, 30
5	156.6		4, 30	
6	125.1		4, 24, 25	

7	141.6		4, 24	
8	124.8		20, 24	
9	195.1		19	
10	80.4		19, 20	
11	199.9		19	
12	110.5		14	
13	164.2		14	
14	103.2	6.4 (1H, s)		
15	166.1		14	
16	115.0		14	
17	149.5		19, 26, 27	
18	41.4		19, 20, 26, 27	
19	51.4	2.5 (1H, dd, 6.18, 10.26,) 3.6 (1H, m);	20, 26, 27	20, 26, 27
20	30.4	2.7 (1H, dd, 10.26, 18.25,)	19	19, 26, 27
21	143.1		20	
22	122.1		20, 24	
23	160.3		24, 28	
24	115.6	6.7 (1H, s)		25, 28
25	18.2	2.0 (3H, s)		24
26	27.4	1.7 (3H, s)	26	19, 20
27	29.3	1.9 (3H, s)	19, 27	19, 20
28	57.3	3.9 (3H, s)		24
29	56.6	3.7 (3H, s)		4
30	57.0	3.9 (3H, s)		4

^{1}H NMR of **8**

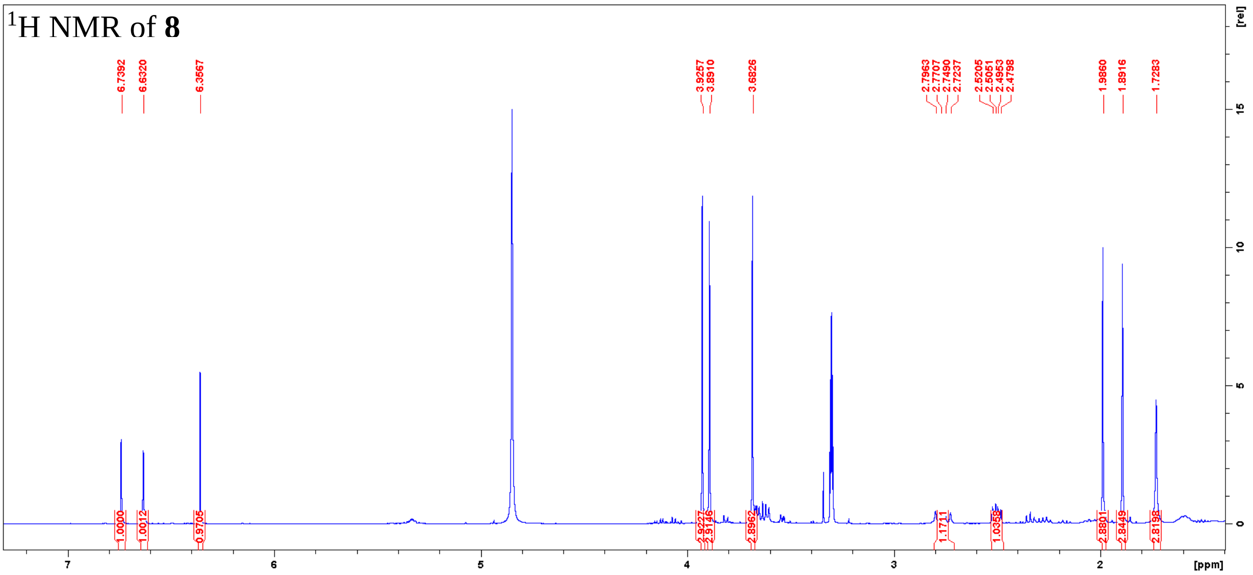

^{13}C NMR of **8**

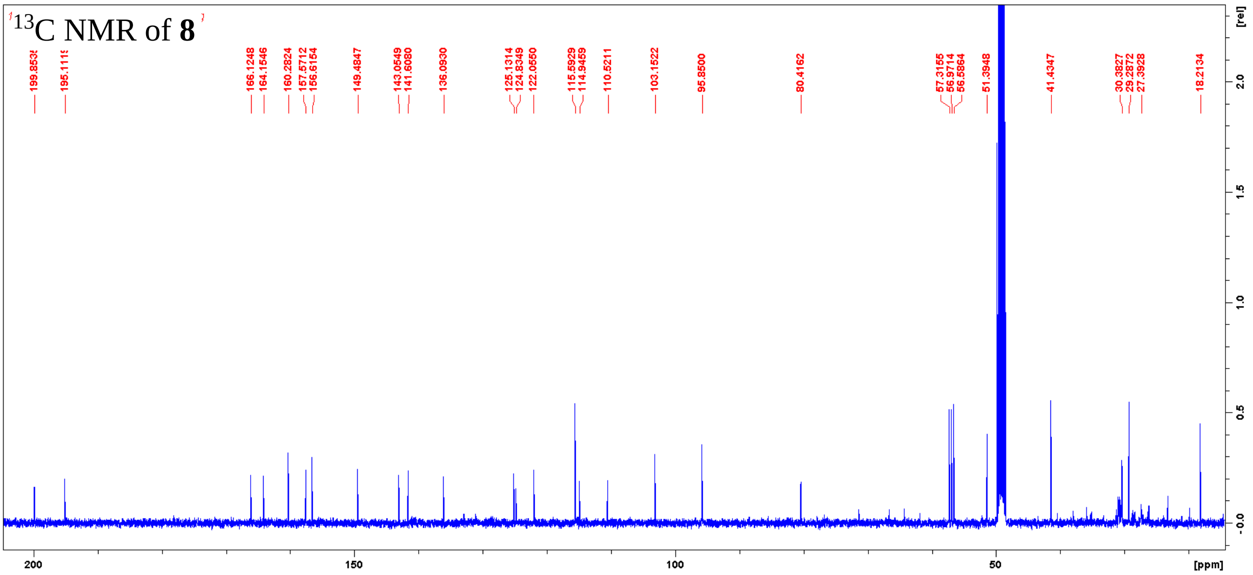

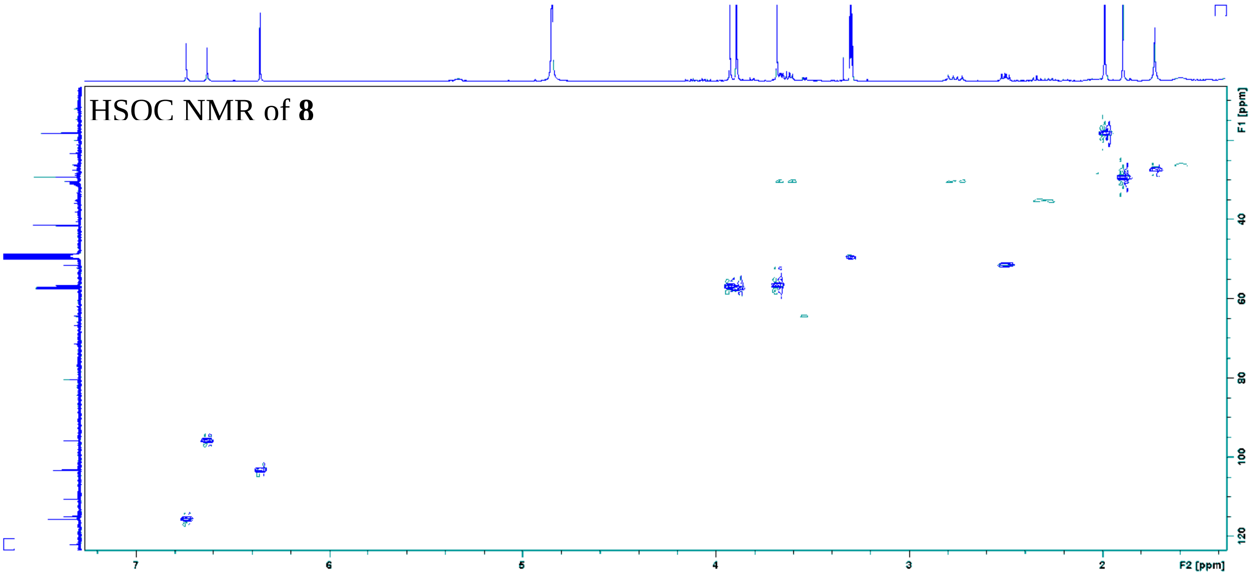
HSQC NMR of **8**
F1 [ppm]
F2 [ppm]
7
6
5
4
3
2
20
40
60
80
100
120

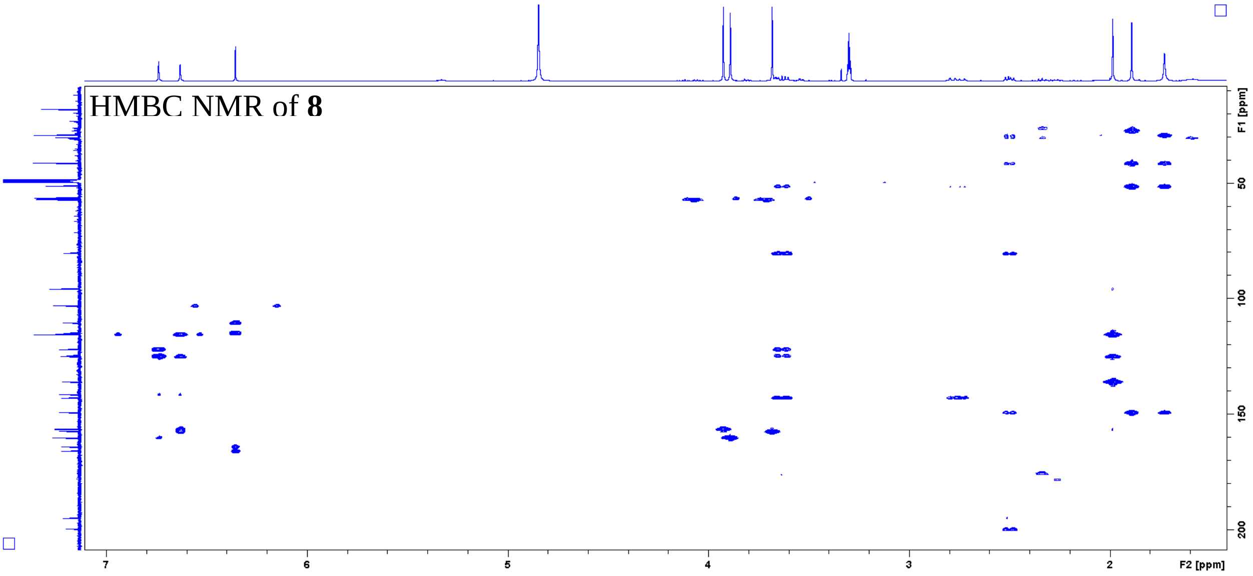
HMBC NMR of **8**
F1 [ppm]
F2 [ppm]
7
6
5
4
3
2
50
100
150
200

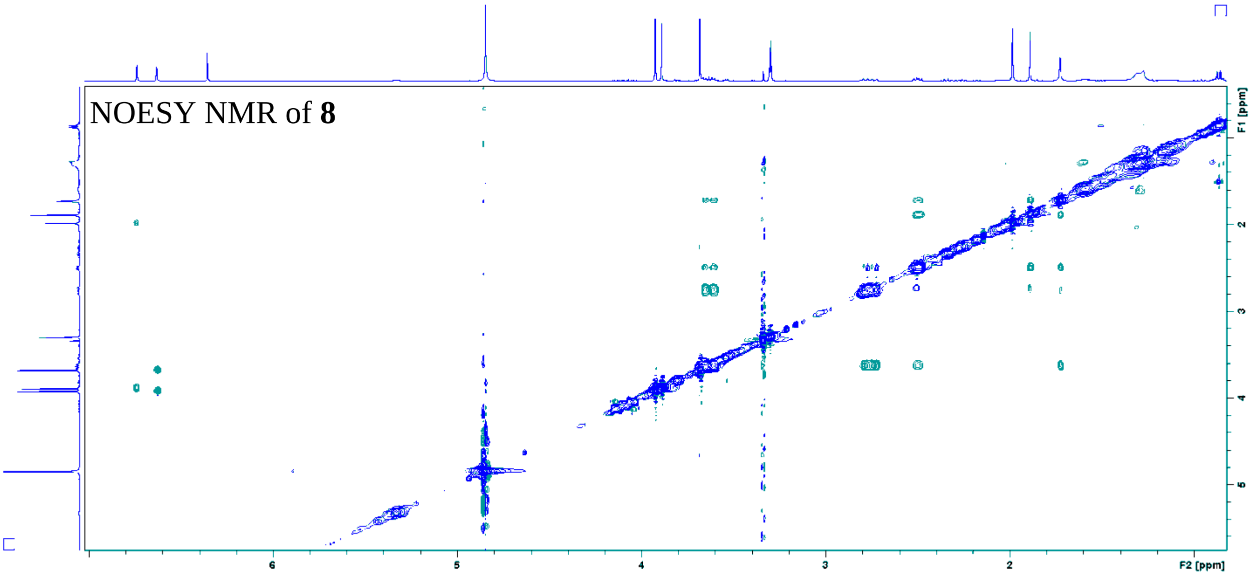

Formicamycin F (9)

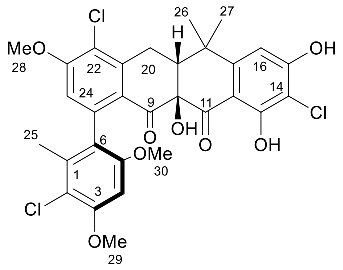

Molecular formula: $C_{30}H_{27}O_8Cl_3$

Isolated yield: 3 mg

UV (PDA): λ_{max} = 232 and 292 nm

Optical activity: $[\alpha]_D^{20}$ = +228.30

HRMS (ESI) *m/z*: calculated $[M + H]^+$ = 621.0844, observed $[M + H]^+$ = 621.0848, Δ = 0.64 ppm

NMR (CD_3OD; 400 MHz & 100 MHz): ^1H, ^{13}C, HSQC, HMBC, NOESY

Position	δ_C ppm	δ_H ppm (no. of protons, multiplicity, J in Hz)	HMBC	NOESY
1	136.2		25	
2	115.6		4, 25	
3	157.6		4, 29	
4	96.01	6.6 (1H, s)		29, 30
5	156.7		4, 30	
6	125.1		4, 24, 25	

7	141.7		24	
8	125.0		24	
9	195.1			
10	80.3		19, 20	
11	199.0			
12	109.5		16	
13	162.5			
14	107.0		16	
15	163.7		16	
16	108.2	6.6 (1H, s)		26, 27
17	153.0		19, 26, 27	
18	39.5		16, 19, 20, 26, 27	
19	49.2	2.6 (1H, dd, 8.92, 6.57)	20, 26, 27	20, 26
20	30.1	3.5 (1H, dd, 18.88, 6.57);<br>2.7 (1H, dd, 18.88, 8.92)	19	
21	143.2		20	
22	122.0		20, 24	
23	160.3		28	
24	115.7	6.7 (1H, s)		25, 28
25	18.2	2.0 (3H, s)		24
26	29.2	1.4 (3H, s)	19, 26	16, 26
27	34.2	1.6 (3H, s)	27	16, 19, 27
28	57.3	3.9 (3H, s)		24
29	56.6	3.7 (3H, s)		4
30	57.0	4.0 (3H, s)		4

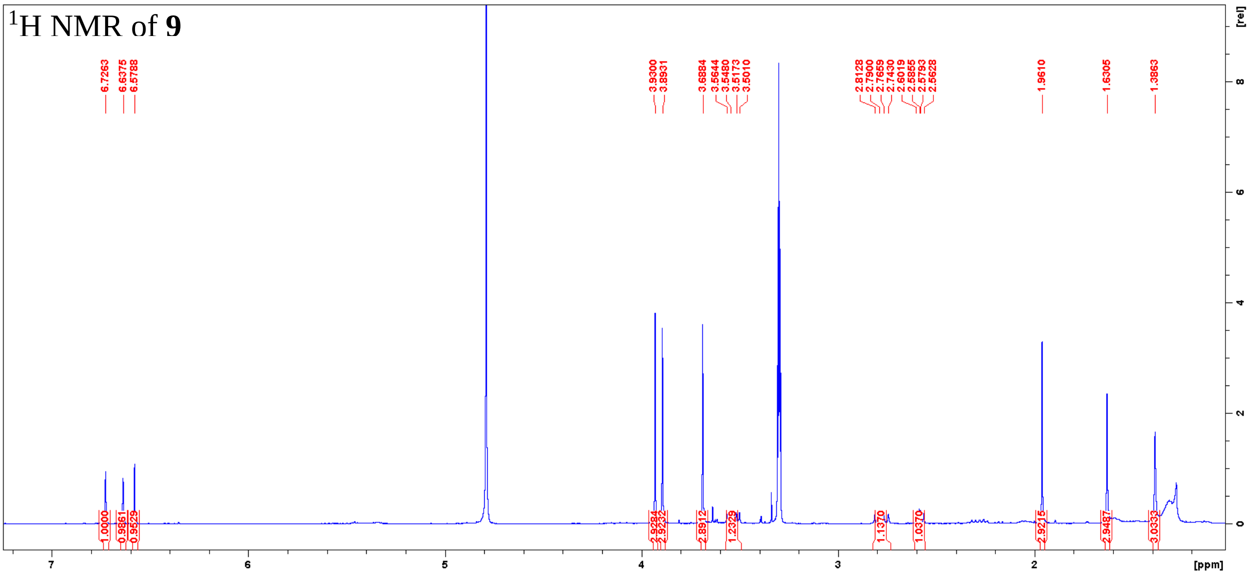
^{1}H NMR of **9**

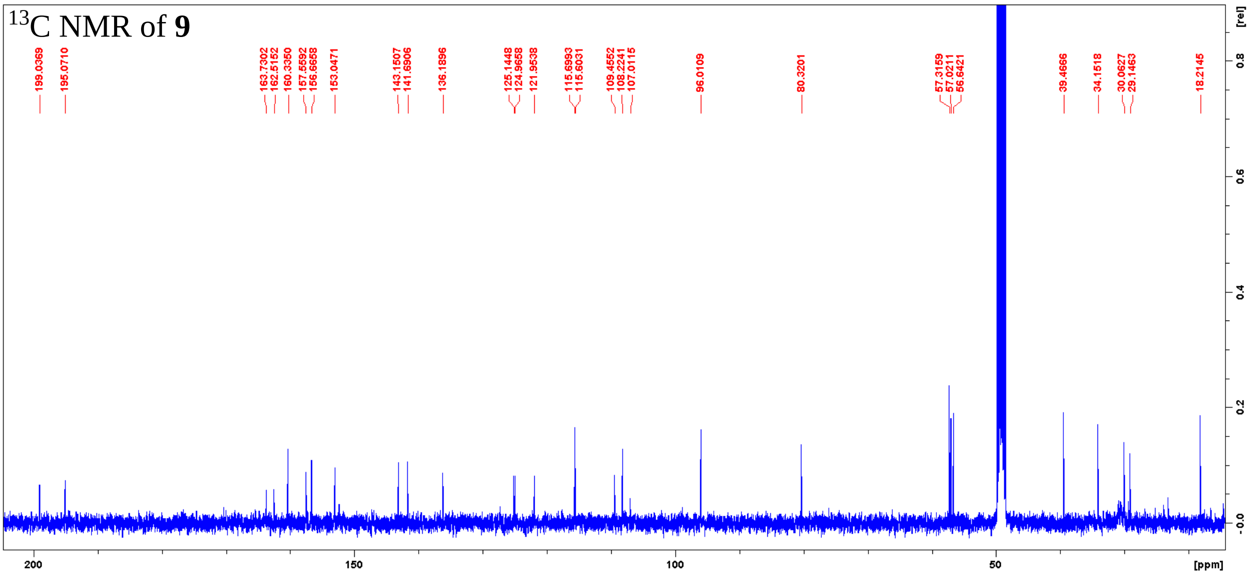
^{13}C NMR of **9**

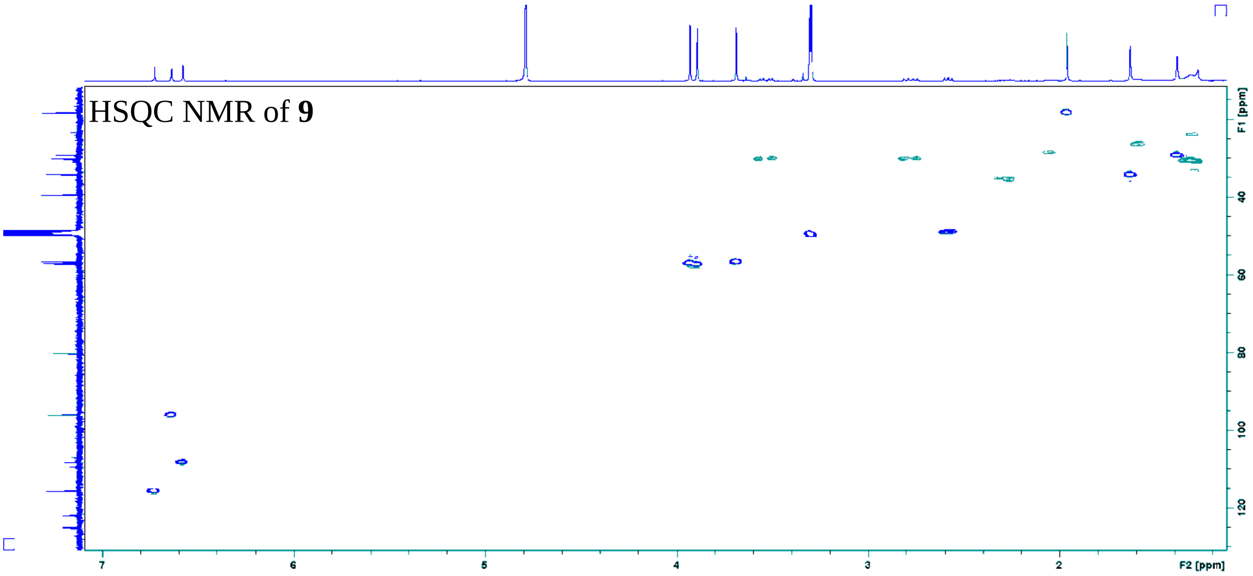
HSQC NMR of **9**
F1 [ppm]
F2 [ppm]
7
6
5
4
3
2
20
40
60
80
100
120

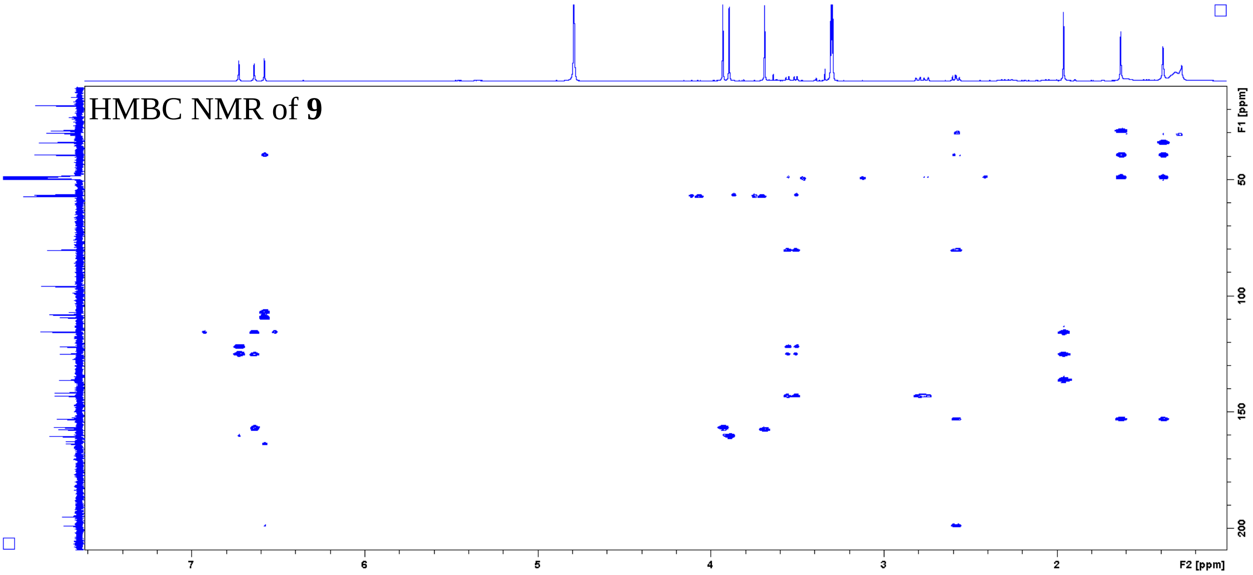
HMBC NMR of **9**
F1 [ppm]
F2 [ppm]
7
6
5
4
3
2
50
100
150
200

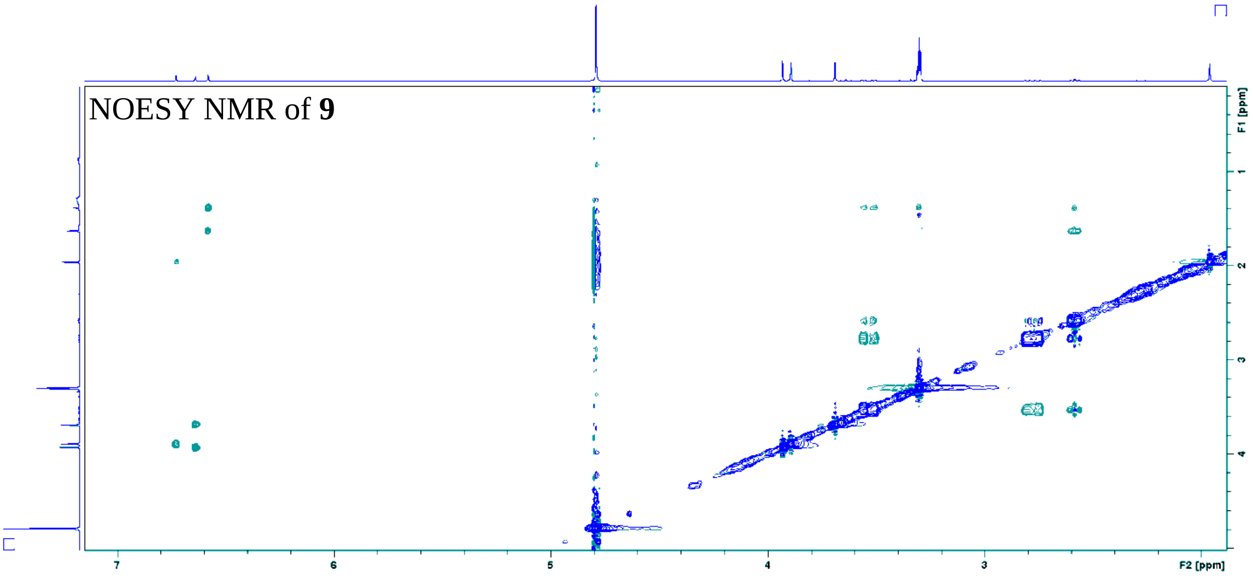
NOESY NMR of **9**
F1 [ppm]
F2 [ppm]
7
6
5
4
3
1
2
3
4

Formicamycin G (10)

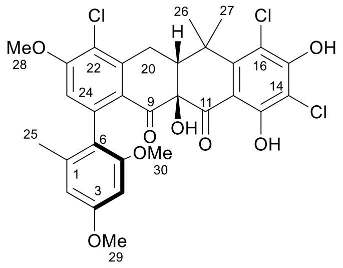

Molecular formula: $C_{30}H_{27}O_8C_{l3}$

Isolated yield: 1 mg

UV (PDA): λ_{max} = 236 and 289 nm

Optical activity: $[\alpha]_D^{20}$ = +469.33

HRMS (ESI) *m/z*: calculated $[M + H]^+$ = 621.0844, observed $[M + H]^+$ = 621.0859, Δ = 2.42 ppm

NMR (CD_3OD; 400 MHz & 100 MHz): ^1H and NOESY

Position	δ_H ppm (no. of protons, multiplicity, J in Hz)	NOESY
2	6.4 (1H, d, 2.23)	25, 29
4	6.4 (1H, d, 2.23)	30
19	2.4 (1H, dd, 9.75, 6.33)	20, 27
20	2.8 (1H, dd, 18.94, 6.33); 3.6 (1H, dd, 18.94, 9.75)	19, 26
24	6.7 (1H, s)	25, 28
25	1.9 (3H, s)	2, 24
26	1.7 (3H, s)	20, 27
27	1.8 (3H, s)	19, 26
28	3.9 (3H, s)	24
29	3.8 (3H, s)	2
30	3.6 (3H, s)	4

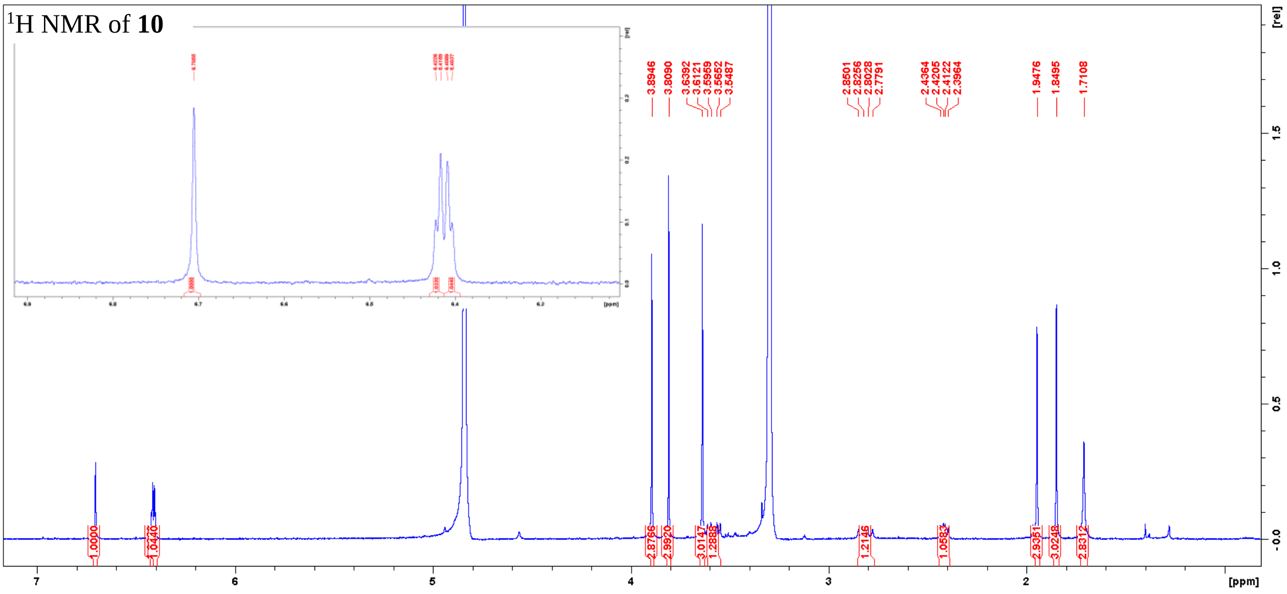

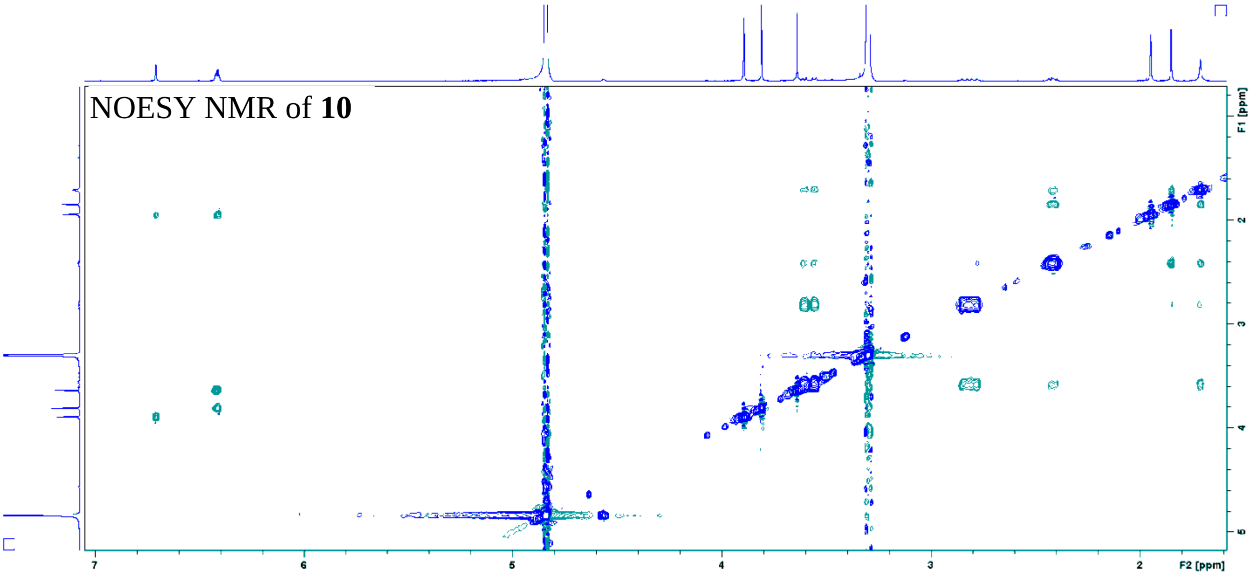

Formicamycin H (11)

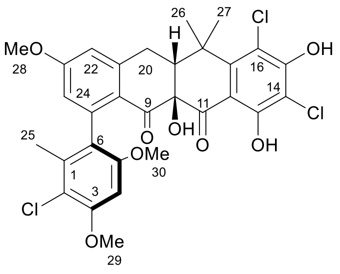

Molecular formulae: $C_{30}H_{27}O_8C_{l3}$

Isolated yield: 0.3 mg

UV (PDA): λ_{max} = 237 and 291 nm

Optical activity: $[\alpha]_D^{20}$ = +275.00

HRMS (ESI) *m/z*: calculated $[M + H]^+$ = 621.0844, observed $[M + H]^+$ = 621.0829, Δ = -2.42 ppm

NMR (CD_3OD; 400 MHz & 100 MHz): ^1H and NOESY

Position	δ_H ppm (no. of protons, multiplicity, J in Hz)	NOESY
4	6.6 (1H, s)	29, 30
19	2.4 (1H, dd, 11.62, 5.80)	20, 26, 27
20	3.0 (1H, dd, 18.55, 11.62); 3.5 (1H, dd, 18.55, 5.80)	19, 22, 26
22	6.8 (1H, d, 2.45)	20, 28
24	6.5 (1H, d, 2.45)	25, 28
25	2.0 (3H, s)	24
26	1.7 (3H, s)	19, 20, 27
27	1.9 (3H, s)	19, 26
28	3.8 (3H, s)	22, 24
29	3.9 (3H, s)	4
30	3.7 (3H, s)	4

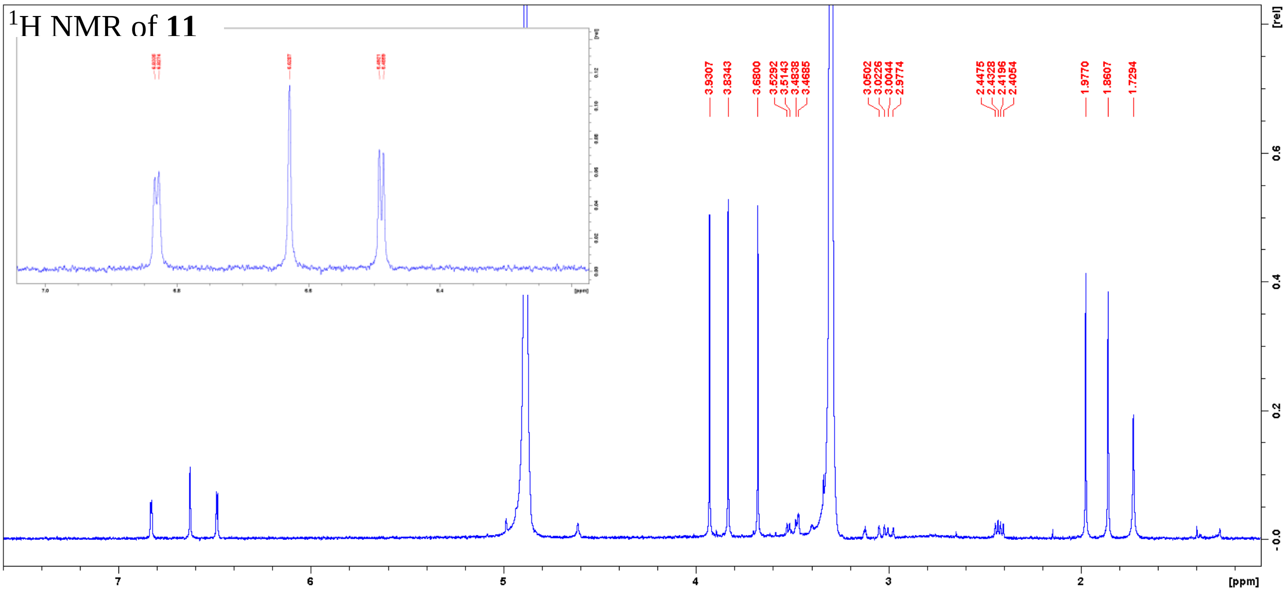

^1H NMR of **11**

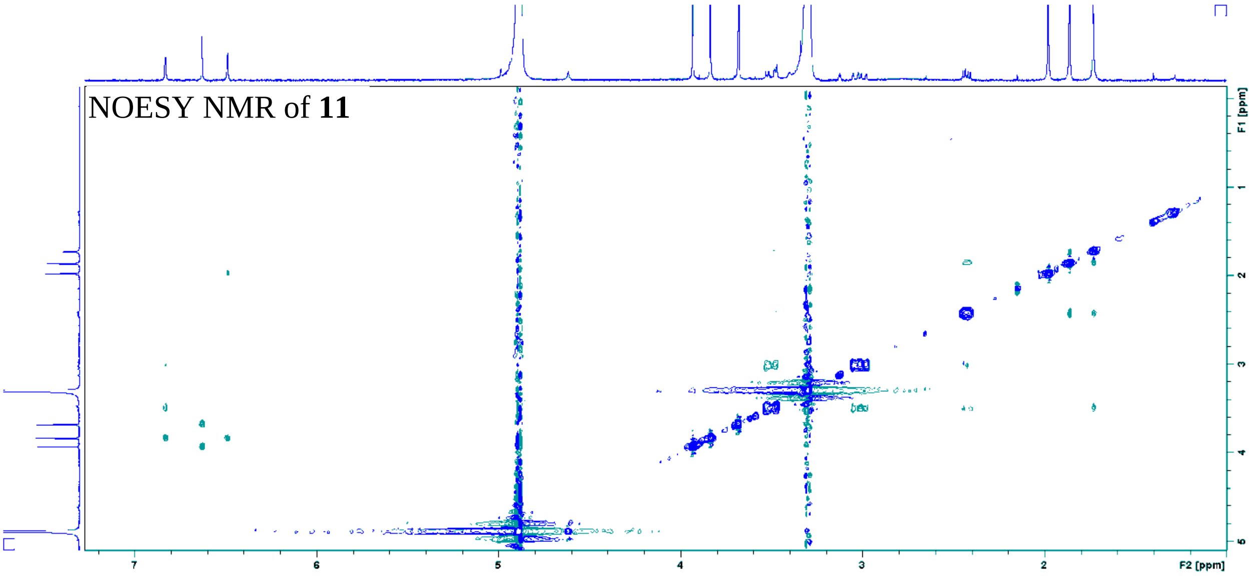

Formicamycin I (12)

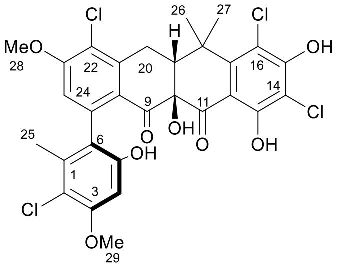

Molecular formula: $C_{29}H_{24}O_8Cl_4$

Isolated yield: 2 mg

UV (PDA): λ_{max} = 233 and 290 nm

Optical activity: $[\alpha]_D^{20}$ = +342.69

HRMS (ESI) *m/z*: calculated $[M + H]^+$ = 641.0298, observed $[M + H]^+$ = 641.0295, Δ = -0.47 ppm

NMR (CD_3OD; 400 MHz & 100 MHz): ^1H, ^{13}C, HSQC, HMBC, NOESY

Position	δ_C ppm	δ_H ppm (no. of protons, multiplicity, J in Hz)	HMBC	NOESY
1	135.6		25	
2	113.9		4, 25	
3	154.2		4, 29	
4	98.7	6.5 (1H, s)		29
5	147.0			
6	124.3		4, 25	

7	124.9		24	
8	not detected			
9	194.8		19	
10	80.4		19, 20	
11	199.9			
12	not detected			
13	161.5			
14	108.9			
15	160.3			
16	109.8			
17	142.9		19, 26, 27	
18	41.4		19, 26, 27	
19	51.3	2.5 (1H, dd, 10.26, 6.19)	26, 27	20, 26, 27
20	30.4	2.8 (1H, dd, 19.20, 10.26); 3.6 (1H, m)		19
21	141.9			
22	122.0		20, 24	
23	157.6		28	
24	115.8	6.7418 (1H, s)		25, 28
25	18.2	1.9786 (3H, s)		24
26	27.5	1.7385 (3H, s)	27	19, 20, 27
27	29.4	1.8997 (3H, s)	19, 26	19, 26
28	57.3	3.9011 (3H, s)		24
29	56.3	3.6158 (3H, s)		4

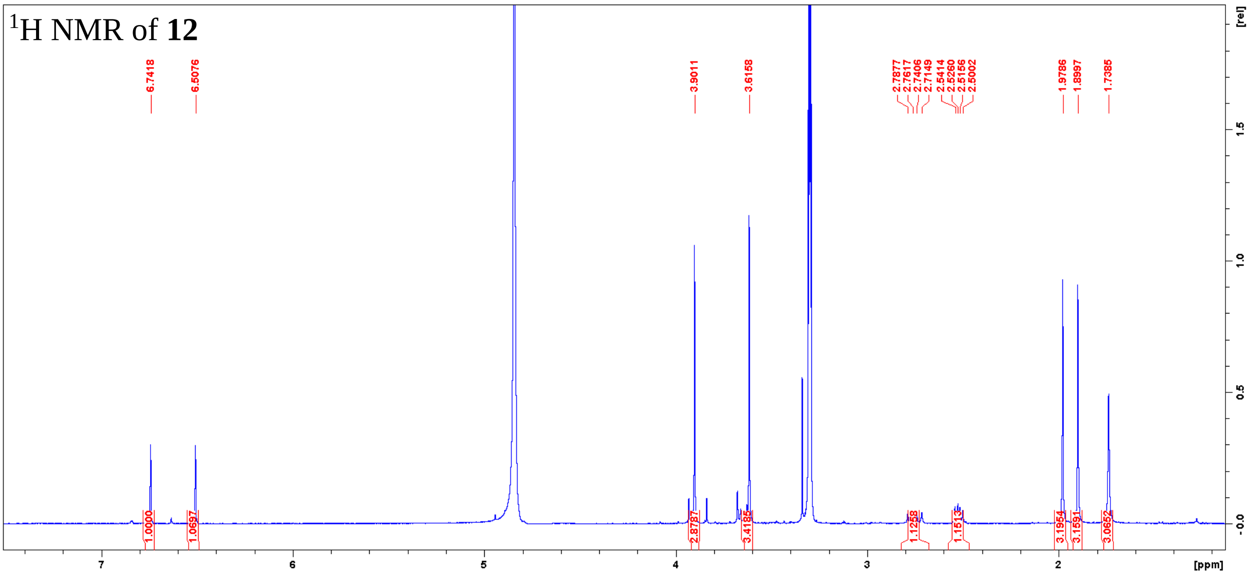
1H NMR of 12
6.7418
6.5076
3.9011
3.6158
2.7877
2.7617
2.7406
2.7149
2.5414
2.5260
2.5156
2.5002
1.9786
1.8997
1.7385
[ppm]
[rel]

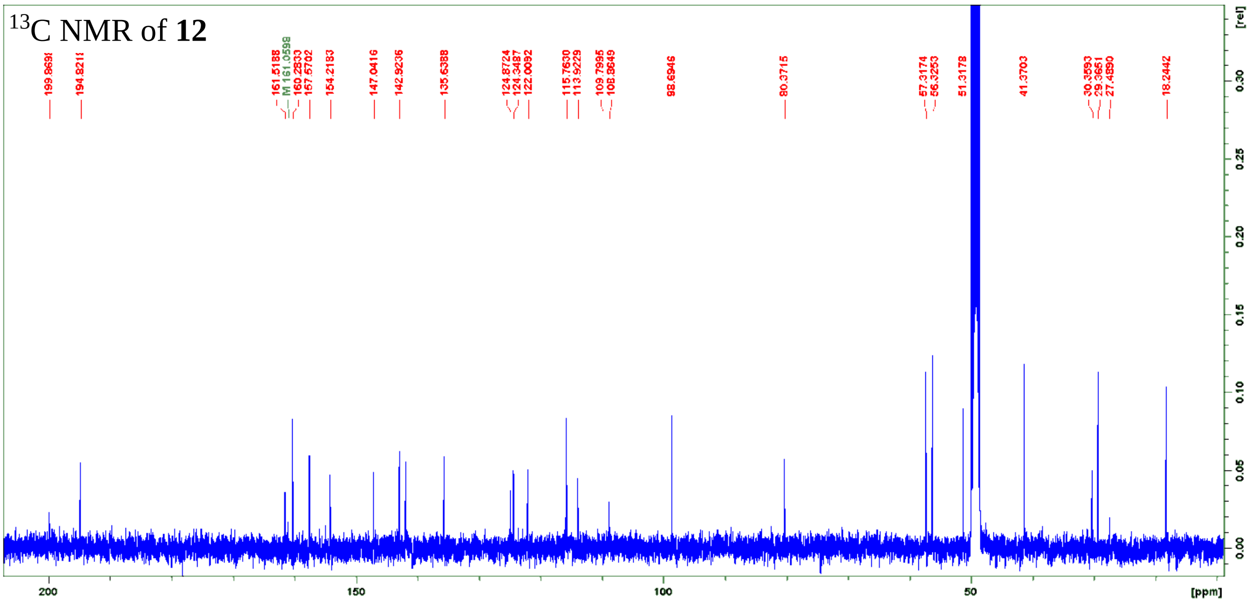
13C NMR of 12
199.9691
194.8211
161.5188
161.0598
160.2333
157.5702
154.2183
147.0416
142.9236
135.6388
124.8724
124.3487
122.0092
115.7630
113.9229
109.7995
108.8649
98.6946
80.3715
57.3174
56.3253
51.3178
41.3703
30.3593
29.3651
27.4890
18.2442
[ppm]
[rel]

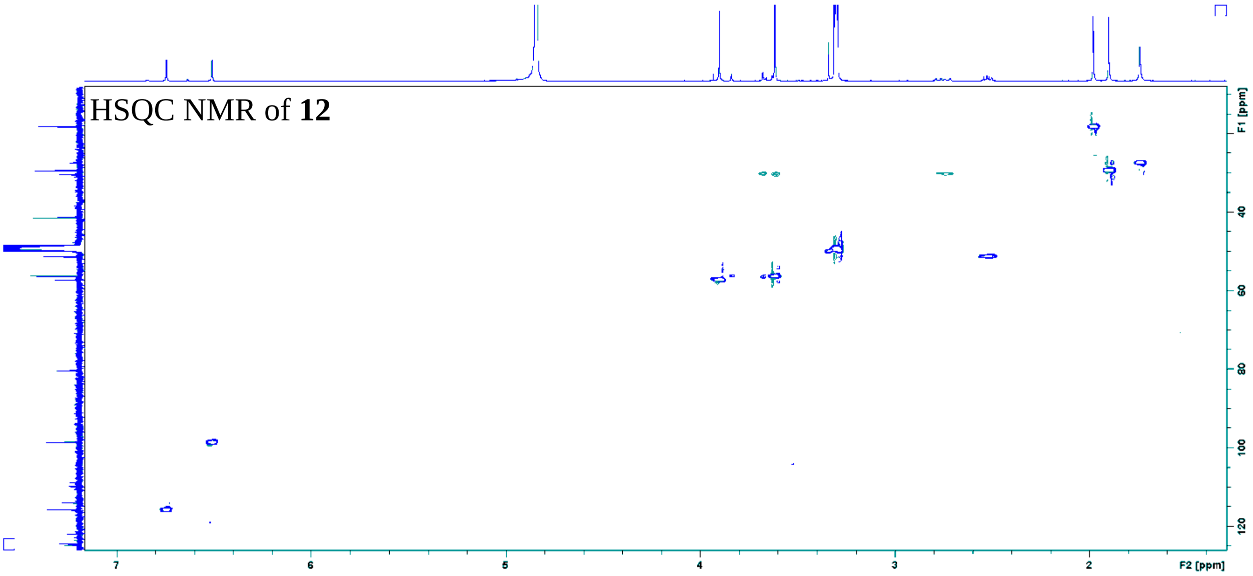
HSQC NMR of 12
F2 [ppm]
F1 [ppm]

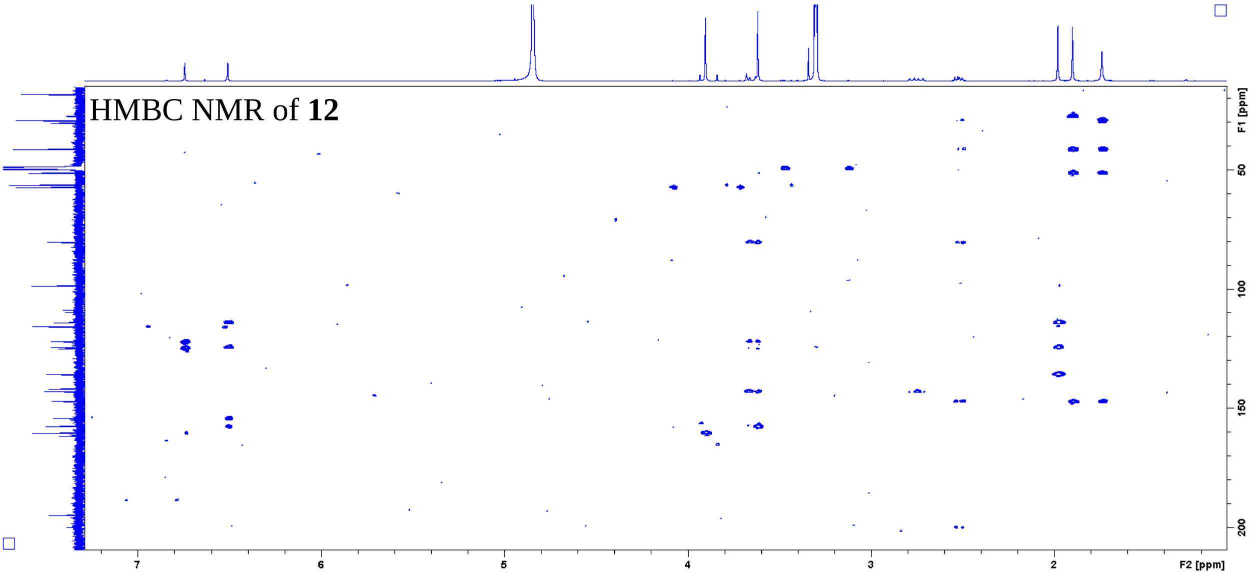

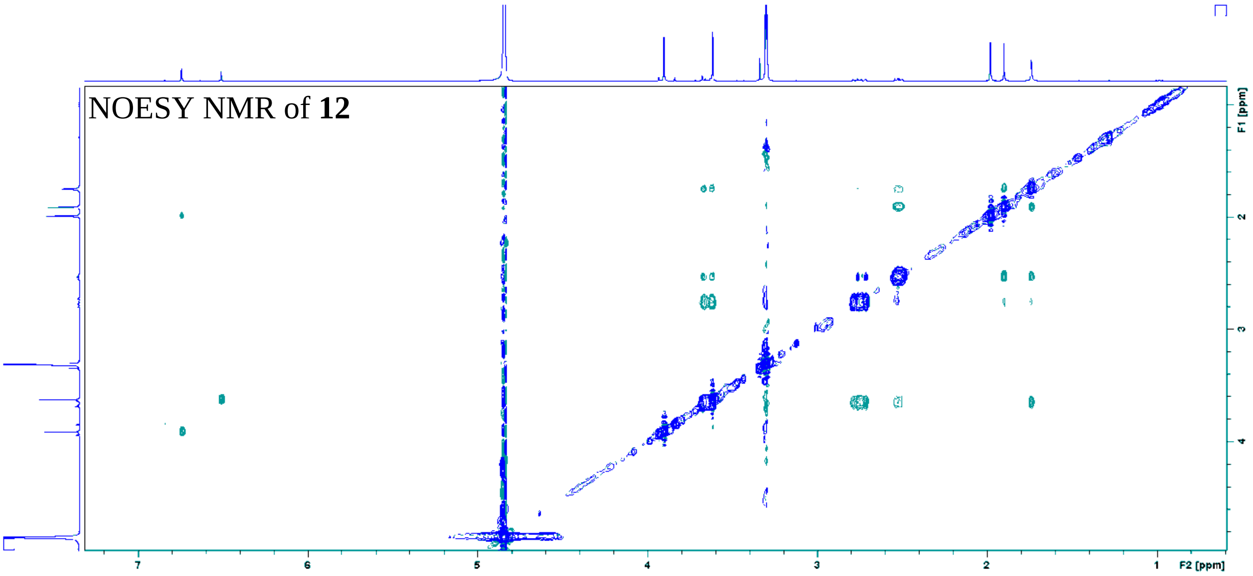

Formicamycin J (13)

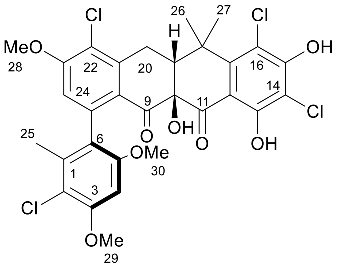

Molecular formula: $C_{30}H_{26}O_8Cl_4$

Isolated yield: 3 mg

UV (PDA): λ_{max} = 232 and 290 nm

Optical activity: $[\alpha]_D^{20}$ = +405.92

HRMS (ESI) *m/z*: calculated $[M + H]^+$ = 655.0455, observed $[M + H]^+$ = 655.0464, Δ = 1.37 ppm

NMR (CD_3OD; 400 MHz & 100 MHz): ^1H, ^{13}C, HSQC, HMBC, NOESY

Position	δ_C ppm	δ_H ppm (no. of protons, multiplicity, J in Hz)	HMBC	NOESY
1	136.1		25	
2	116.5		4, 25	
3	156.6		29	
4	95.9	6.6 (1H, s)		29, 30
5	157.6		4, 30	
6	125.1		4, 25	
7	141.6			
8	124.8		20, 24	
9	195.0		19	
10	80.3		19, 20	
11	199.3		19	
12	109.3			
13	161.6			
14	108.9			
15	162.1			
16	107.7			
17	146.8		19, 26, 27	
18	41.3		19, 20, 26, 27	
19	51.3	2.5 (1H, dd, 10.14, 6.12)	20, 26, 27	20, 26, 27
20	30.4	2.8 (1H, dd, 18.71, 10.14); 3.63 (1H, m)		19
21	143.1		20	
22	122.1		20, 24	
23	160.3		24, 28	
24	115.6	6.7 (1H, s)		25, 28
25	18.2	2.0 (3H, s)		25
26	27.5	1.7 (3H, s)	27	19, 20, 27

27	29.3	1.9 (3H, s)	19, 26	19, 26
28	57.3	3.9 (3H, s)		24
29	57.0	3.9 (3H, s)		4
30	56.6	3.7 (3H, s)		4

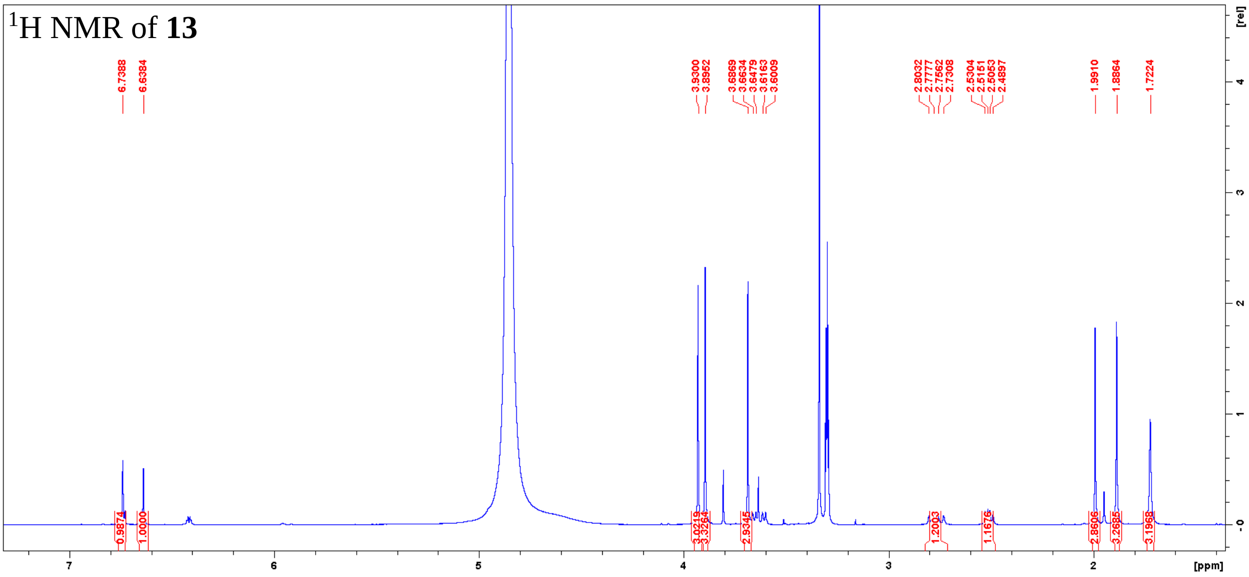

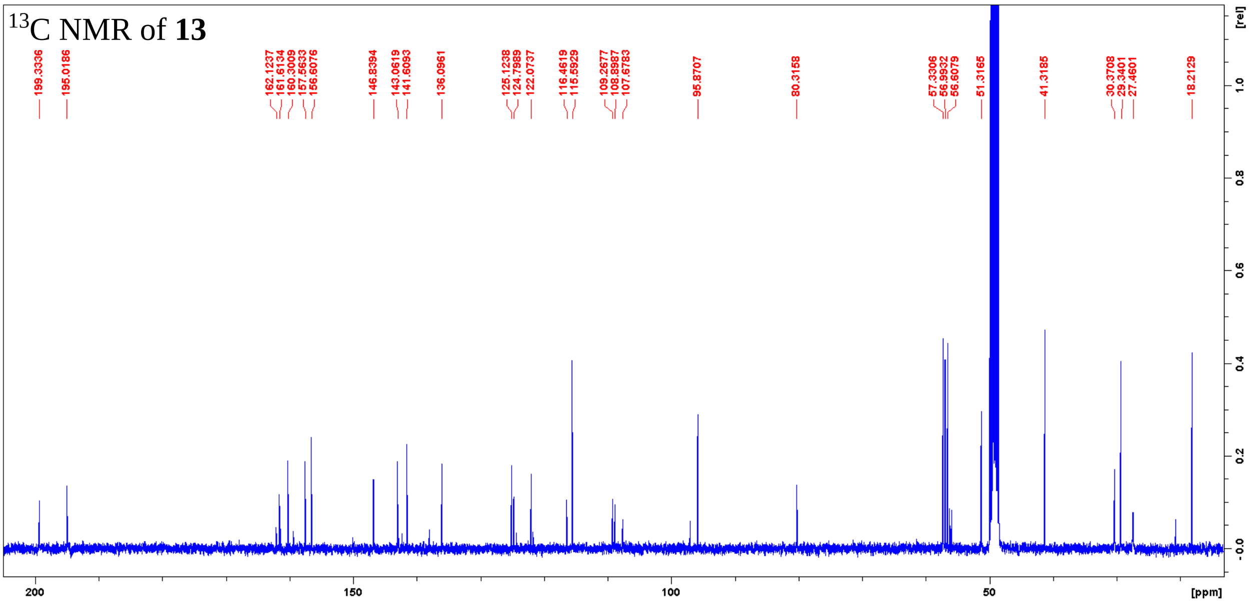

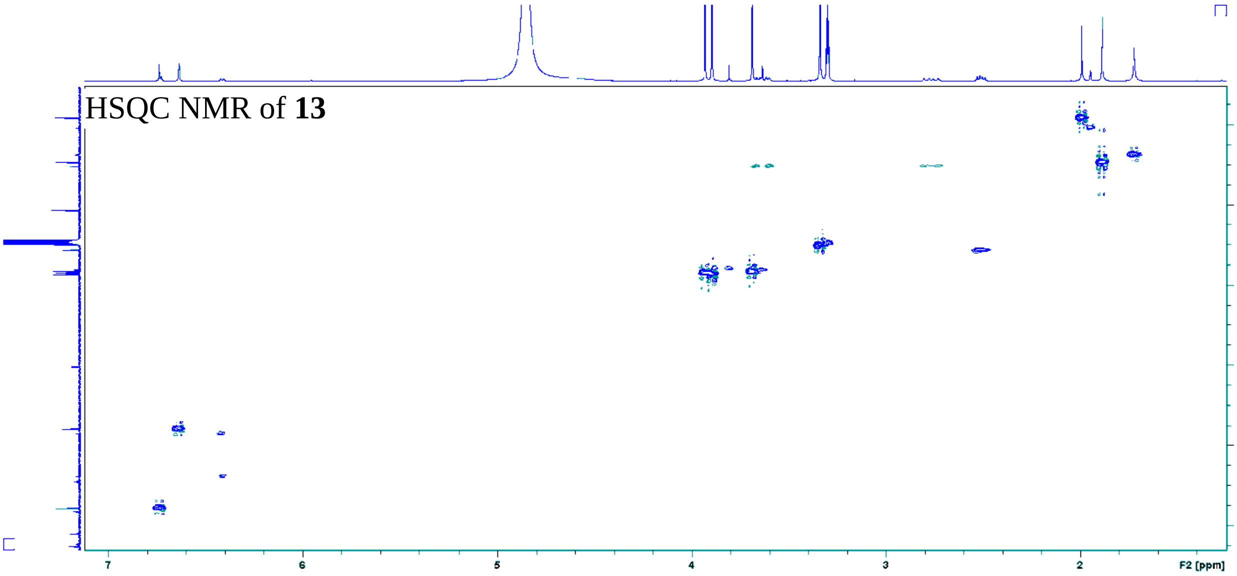
HSQC NMR of **13**
F1 [ppm]
F2 [ppm]

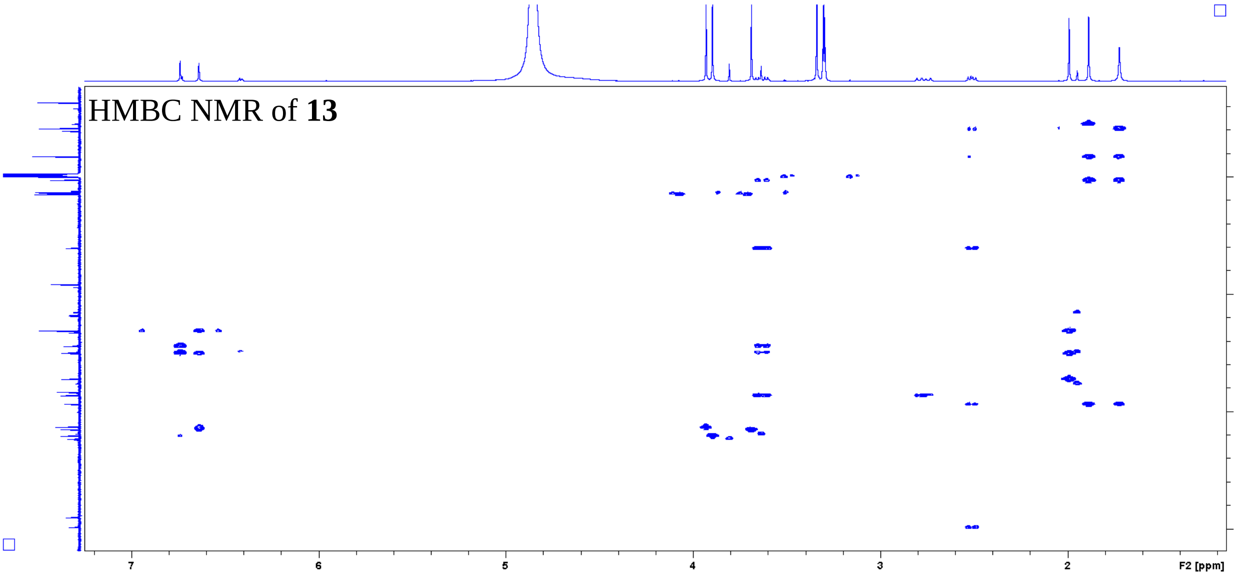
HMBC NMR of **13**
F1 [ppm]
F2 [ppm]

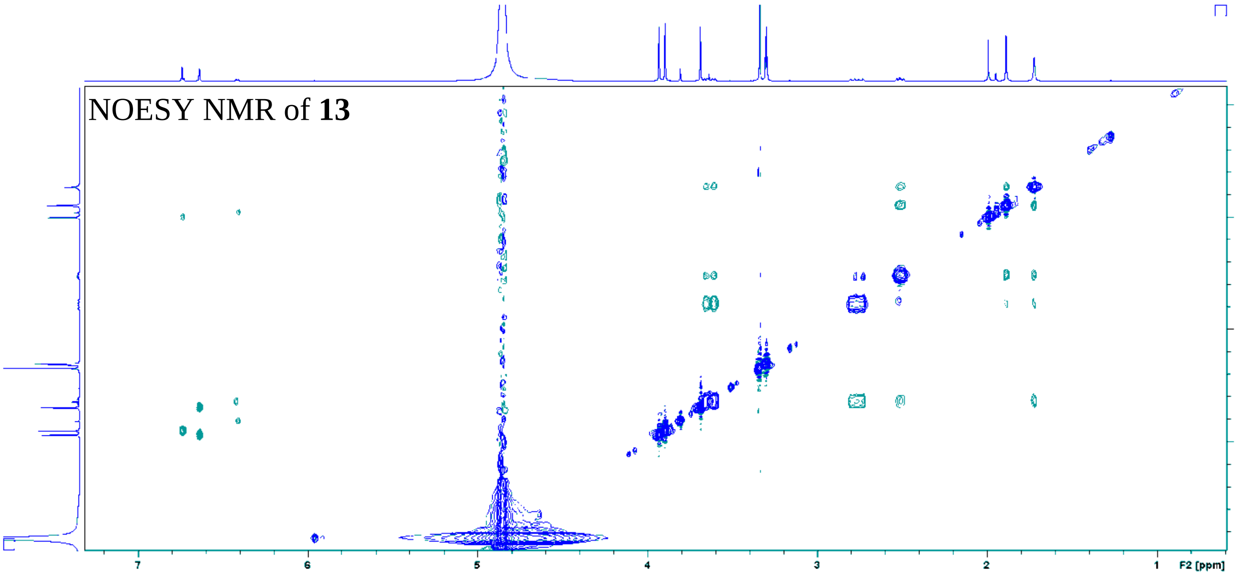
NOESY NMR of **13**
F1 [ppm]
F2 [ppm]

Formicamycin K (14)

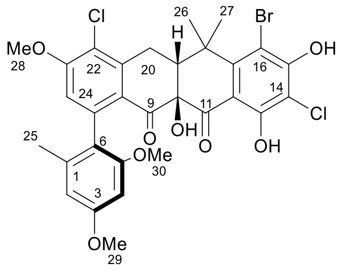

Molecular formula: $C_{30}H_{27}O_8Cl_2Br$

Isolated yield: 0.5 mg

UV (PDA): λ_{max} = 238 and 290 nm

Optical activity: $[\alpha]_D^{20}$ = +303.60

HRMS (ESI) *m/z*: calculated $[M + H]^+$ = 665.0339, observed $[M + H]^+$ = 665.0334, Δ = -0.75 ppm

NMR (CD_3OD; 400 MHz): ^1H

Position	δ_H ppm (no. of protons, multiplicity, J in Hz)
2	6.4 (1H, d, 2.35)
4	6.4 (1H, d, 2.35)
19	2.5 (1H, dd, 10.33, 6.21)
20	2.8 (1H, dd, 19.32, 10.33); 3.5793 (m)
24	6.7 (1H, s)
25	1.9 (3H, s)
26	1.7 (3H, s)
27	1.9 (3H, s)
28	3.9 (3H, s)
29	3.8 (3H, s)
30	3.6 (3H, s)

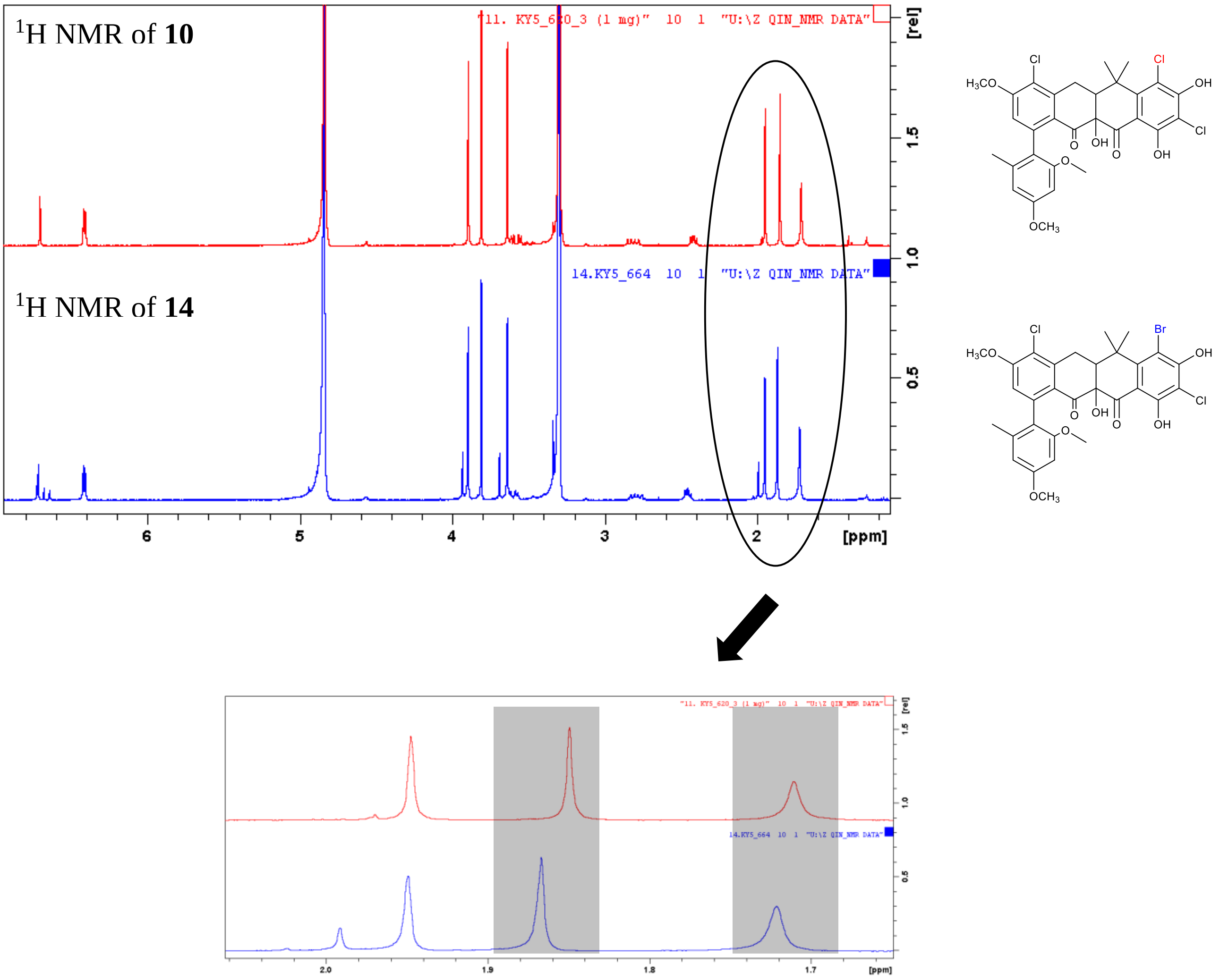

Formicamycin L (15)

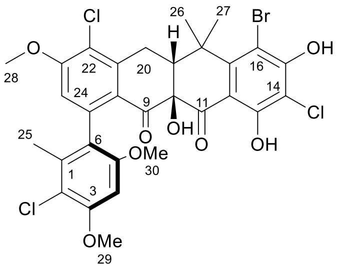

Molecular formula: $C_{30}H_{26}O_8Cl_3Br$

Isolated yield: 0.6 mg

UV (PDA): λ_{max} = 236 and 291 nm

Optical activity: $[\alpha]_D^{20}$ = +390.32

HRMS (ESI) *m/z*: calculated $[M + H]^+$ = 698.9949, observed $[M + H]^+$ = 698.9962, Δ = 1.86 ppm

NMR (CD_3OD; 400 MHz): ^1H

Position	δ_H ppm (no. of protons, multiplicity, J in Hz)
4	6.6 (1H, s)
19	2.5 (1H, dd, 10.07, 6.28)
20	2.8 (1H, dd18.69, 10.07); 3.6 (1H, m)
24	6.7 (1H, s)
25	2.0 (3H, s)
26	1.7 (3H, s)
27	1.9 (3H, s)
28	3.9 (3H, s)
29	3.9 (3H, s)
30	3.7 (3H, s)

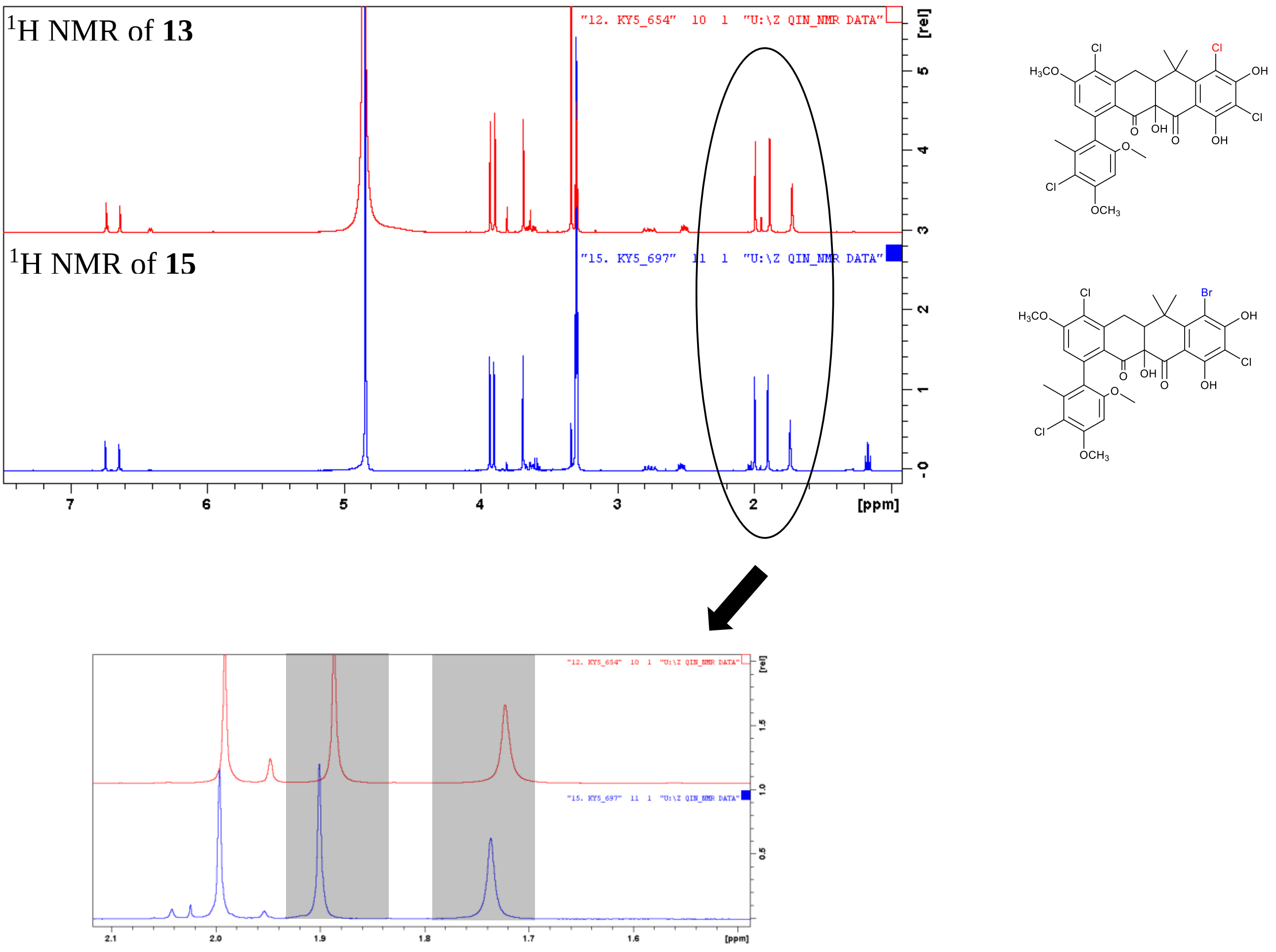

Formicamycin M (16)

Molecular formula: $C_{30}H_{29}O_8Br$

HRMS (ESI) *m/z*: calculated $[M + H]^+$ = 597.1119, observed $[M + H]^+$ = 597.1120, Δ = 0.17 ppm

1f. Stable isotope labelling experiment. *S. formicae* was cultivated on MS agar (2L) at 30°C and overlaid with [1,2-$^{13}C_2$] sodium acetate (1 mL of a 60 mM solution) after 24h, 48h, 72h and 96h. After a further 48 h the agar was extracted and purified using the methods described above to yield a sample of **4** (5 mg). This material was analyzed by ^{13}C NMR (100 MHz; 15000 scans; d_4-methanol). The coupling constants (J_{CC}) of the intact acetate units were recorded as the follows: C25-C1: 44.58 Hz; C2-C3: 68.52 Hz; C4-C5: 70.84Hz; C6-C7: 57.47 Hz; C8-C9: 58.01 Hz; C10-C11: 44.95 Hz; C12-C13: 60.42 Hz; C14-C15: 66.81 Hz; C16-C17: 63.35 Hz; C20-C21: 41.67 Hz; C22-C23: 76.58 Hz. C18 has a coupling constant of 34.08; however, the coupling constants for the adjacent C19 cannot be detected due to the overlapping solvent signal.

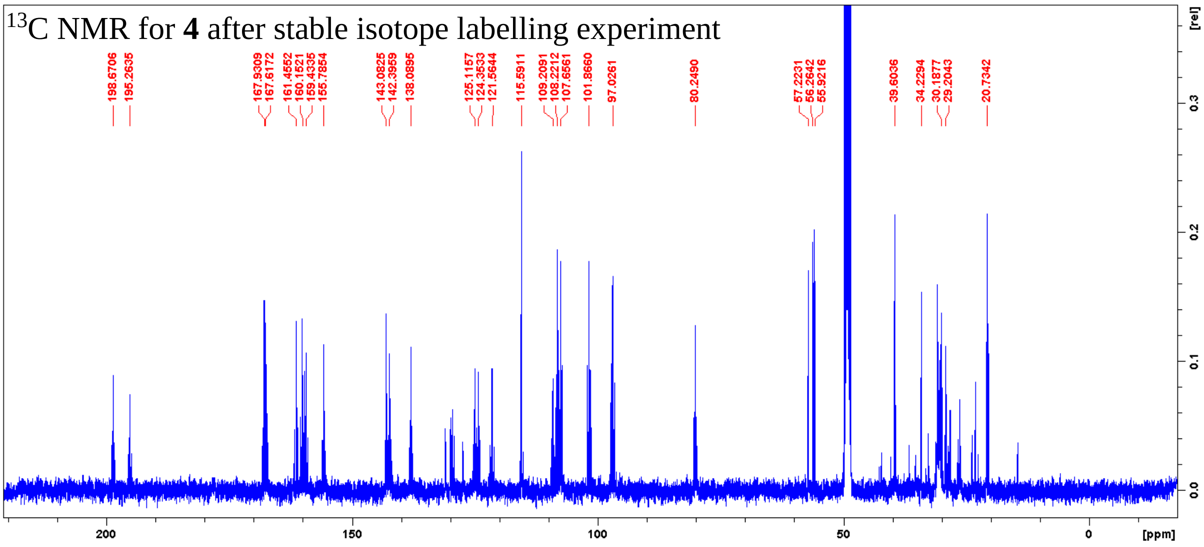

Expanded ^{13}C NMR for labelled **4**

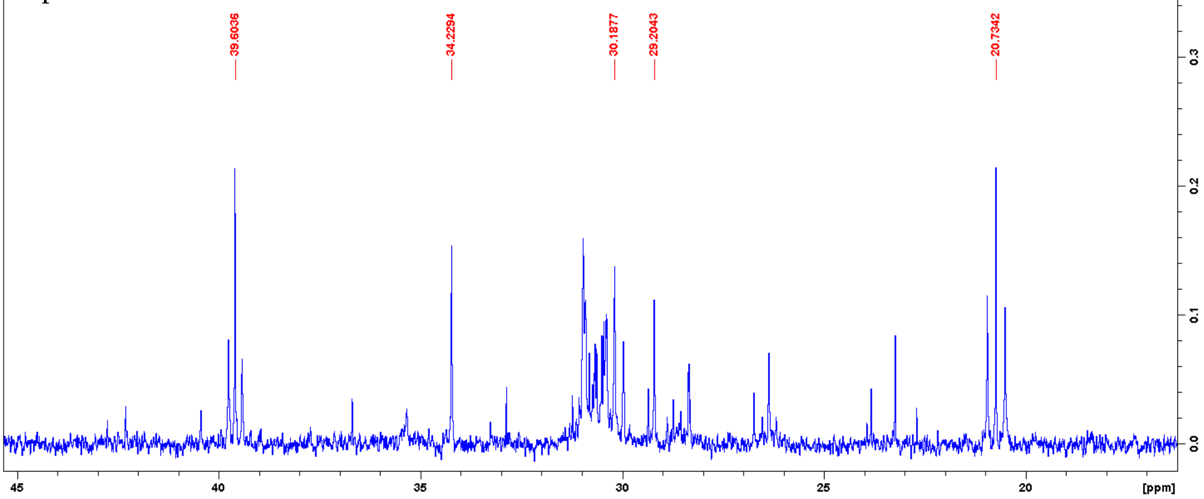

Expanded ^{13}C NMR for labelled **4**

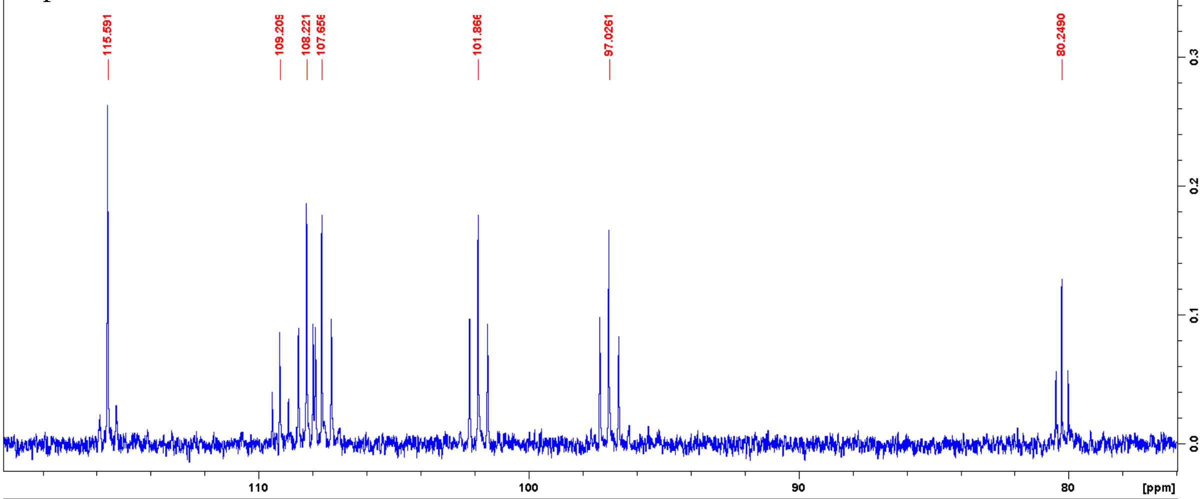

Expanded ^{13}C NMR for labelled **4**

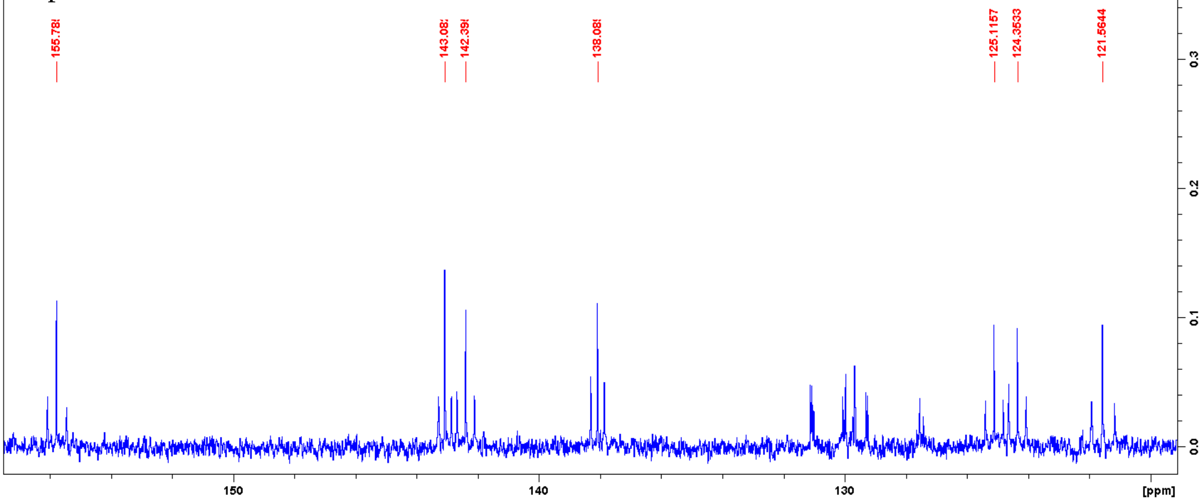

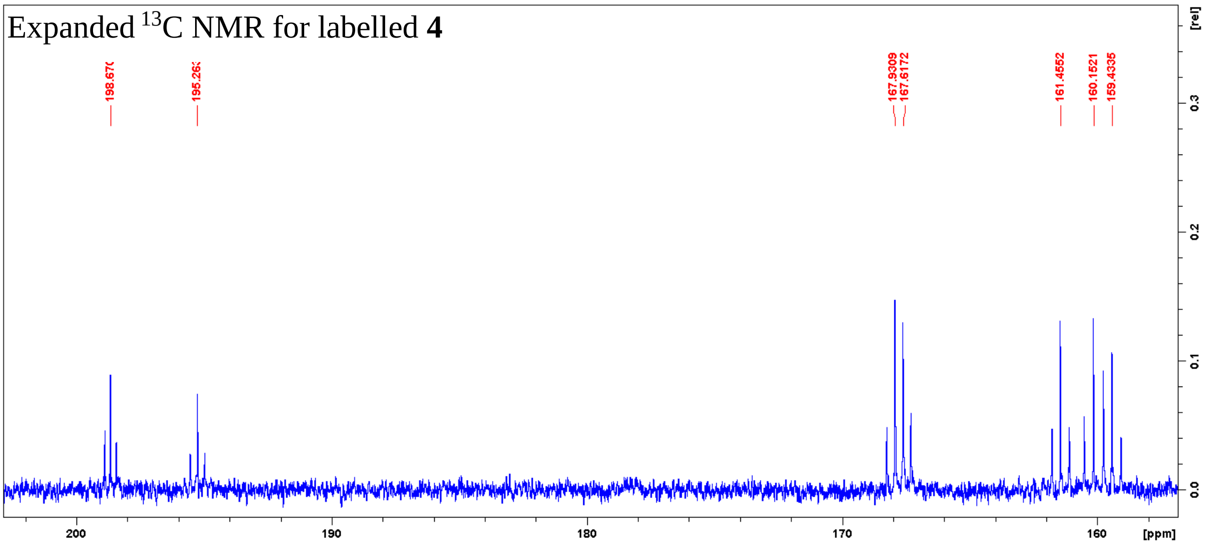

1g. MIC determination. Growth studies were carried out to determine the minimum inhibitory concentration (MIC) of each of the isolated compounds. The primary indicator strains included a laboratory strain of *Bacillus subtilis* and clinical isolates of MRSA and VRE. The clinical isolate of VRE (*Enterococcus faecium* B16.06226) was isolated from a blood culture bootle at the Norfolk and Norwich University Hospital (UK) and is resistant to vancomycin and amoxicillin but sensitive to linezolid and tigecycline; it was provided courtesy of Professor Andrew Hart, Dr Catherine Tremlett and Ashleigh Crane. The clinical isolate of MRSA was isolated from a patient sample at the Norfolk and Norwich University Hospital (UK) and validated using a PCP2a test; it was provided courtesy of Dr Justin O'Grady (UEA Medical School). Cells used were taken from an overnight culture of the indicator strains grown in LB with no NaCl (LB–NaCl). The study was carried out in 24 well plates (Nunclon® Δ Multidishes, 24-well, flat bottom) containing a final volume of 1 ml/well. Each 1 mL contained 40 µL of antibiotic stock (either water or methanol as solvent) with cells added to a final concentration corresponding to an OD_{600} of 0.08-0.15 with the remaining volume consisting of fresh LB-NaCl media. Multi-well plates were measured (OD_{600}) immediately to find an initial 0 h value for each well and incubated at 30°C static, briefly agitated to suspend cells and measured at the desired time points. The MIC was determined as the point where no observable growth could be observed after overnight incubation. For resistance assays, cultures of MRSA were grown in the presence of sub-inhibitory concentrations of compounds **6**, **13** or **15** (0.35 $\mu g.mL^{-1}$, 0.1 $\mu g.mL^{-1}$ and 0.4 $\mu g.mL^{-1}$, respectively) and then sub-cultured following overnight growth for a further 19 generations. The MRSA control culture contained no formicamycins. Each resulting strain was then used to repeat the MIC assay (as described above) to determine if resistance had developed.

1h. CRISPR/Cas9 genome editing. All gene deletions were carried out using the temperature sensitive CRISPR/Cas9 plasmid, pCRISPomyces-2 [8]. All CRISPR/Cas9 editing plasmids were constructed in two steps. First, a gRNA protospacer was cloned into the *Bbs*I site of pCRISPomyces-2 by Golden Gate Assembly following the published protocol [8]. Second, a repair template consisting of ~1 kb of DNA homologous and adjacent to that of the Cas9-induced double strand break was generated by overlap extension PCR with a synthetic linker template (ESI Table 1). The resulting PCR product was verified by sequencing and isolated after restriction digest with *Xba*I and cloned into pCRISPomyces-2 containing the desired protospacer using T4 DNA ligase. The resulting CRISPR/Cas9 editing plasmids were introduced into *E. coli* ET12567/pUZ8002 by electroporation and the resulting strains were used as conjugal donors to *S. formicae* as previously described [2] using either spores or mycelia. The resulting ex-conjugants were colony purified at 30°C on MS agar (20 g soya flour, 20 g mannitol, 20 g agar, 1 L tap water) containing apramycin (50 $\mu g.mL^{-1}$) and then restreaked onto MS agar with no antibiotic selection and grown at 37˚C for 1-3 generations to promote loss of pCRISPomyces2. Strains sensitive to apramycin (indicating plasmid loss) were subsequently amplified by PCR using primers in ESI Table 1 and sequenced to verify the integrity of mutant loci.

1i. Ectopic expression of BGC30. Triparental mating experiments were set up between three *E. coli* strains as described previously [9]. Briefly, *E. coli* DH10β (*neo*) containing ePAC clone pESAC13-215-G (supplied by Bio S&T Inc, *aph*II, *tsr*), *E. coli* Top10 containing the driver plasmid pR9604 (*bla*), and the non-methylating conjugation *E. coli* strain ET12567 (*neo*, *cat*) were grown overnight in LB + antibiotics (100 $\mu g.mL^{-1}$ carbenicllin or 50 $\mu g.mL^{-1}$ kanamycin or 25 $\mu g.mL^{-1}$ chloramphenicol) and sub-cultured the following day to exponential phase (OD_{600} ~0.6). The resulting cells were centrifuged and the medium removed, and then were washed in LB to remove antibiotics. 20 μL spots of each strain were plated in the centre of an LB plate without antibiotic selection. This was incubated at 37°C overnight and then re-streaked onto LB + carbenicillin + kanamycin + chloramphenicol the following day. Six resulting colonies were then picked and grown in liquid LB and three were confirmed by PCR (using primers in ESI Table 1) to contain the whole BGC. These strains were then used to set up conjugation reactions between heat-shocked *S. formicae* Δ*for* spores in 2YT liquid medium [2] and plated onto MS agar + 10 mM Mg^{2+}. After 18 h incubation at 30°C plates were overlaid with (final concentrations) thiostrepton (30 $\mu g.mL^{-1}$) and nalidixic acid (20 $\mu g.mL^{-1}$). After five days the resulting colonies were re-streaked onto MYM agar (4 g maltose, 4 g yeast extract, 10 g malt extract, 1 L 50:50 tap water and dH_2O, pH 7.4) + nalidixic acid (25 µg.mL-

^{1}) + thiostrepton (25 µg.mL^{-1}) and the presence of the BGC was again confirmed by PCR using primers described in ESI Table 1.

1j. Genetic complementation of ForV. *forV* was amplified by PCR along with the native KY5 promotor and cloned into the integrative vector pMS82 using Gibson assembly after digestion with *Hind*III. Once assembled, plasmids were confirmed by PCR and sequencing before being transformed into *E. coli* ET12567/pUZ8002 and conjugated into *S. formicae* Δ*forV*. Ex-conjugants were colony purified at 30°C on MYM agar and confirmed using PCR and sequencing. Extracts from successful complementation strains were then analysed by HPLC and LCMS to detect the presence of fasamycins/formicamycins.

2. ESI Figures

Due to its scales ESI Figure 1 is deposited as an independent PDF.

Figure S1. An approximate maximum likelihood phylogenetic tree of sequenced streptomycetes. A phylogeny was inferred for *Mycobacterium tuberculosis* and 573 sequenced streptomycetes based on concatenated partial sequences for *atpD-rpoB*. FastTree local support values are indicated at each node. The cutaway region shows *Streptomyces formica* (highlighted blue). A full scale phylogenetic tree is available from FigShare at: https://dx.doi.org/10.6084/m9.figshare.3507545.v1 where the scale bar shown indicates 6% estimated sequence divergence. The phylogenetic tree can also be downloaded in Nerwick format from FigShare at: https://dx.doi.org/10.6084/m9.figshare.3507536.v1.

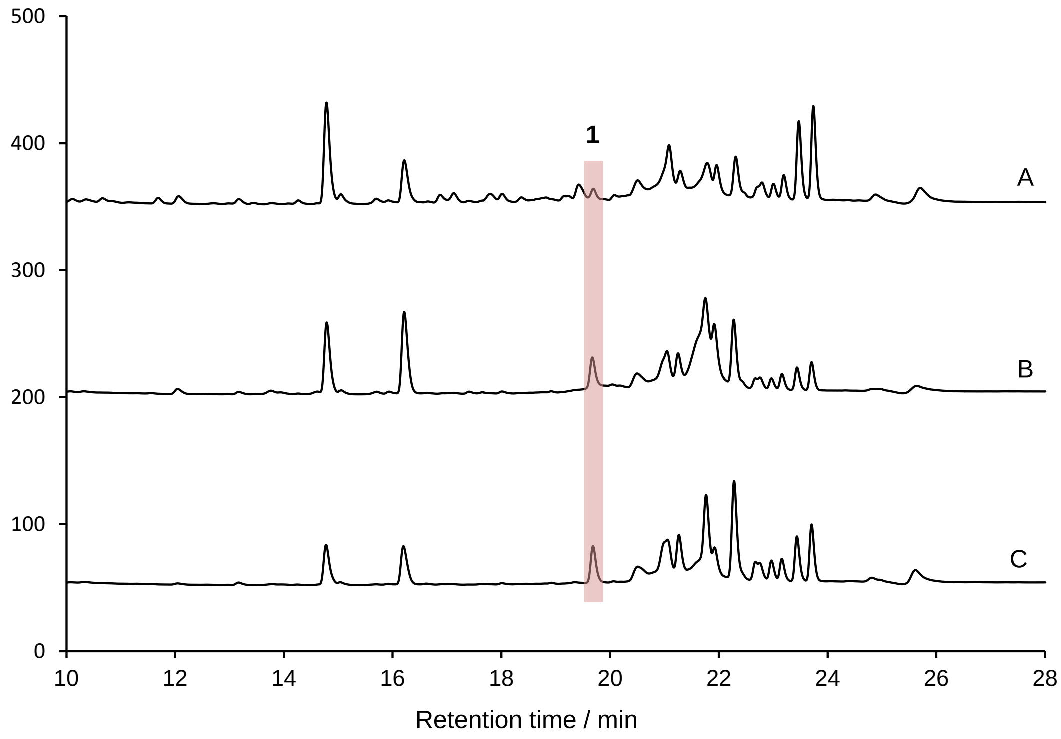

Figure S2. HPLC traces (250 nm) of methanol extracts from *S. formicae* growing on MS agar in the presence of the weak HDAC inhibitor sodium butyrate[10]. This leads to significantly enhanced production of the otherwise minor congener **1** (Y-axis scales showing absorbance have been normalized). (A) no sodium butyrate; (B) 10 mM sodium butyrate; (C) 50 mM sodium butyrate. (HDAC, histone deacetylase).

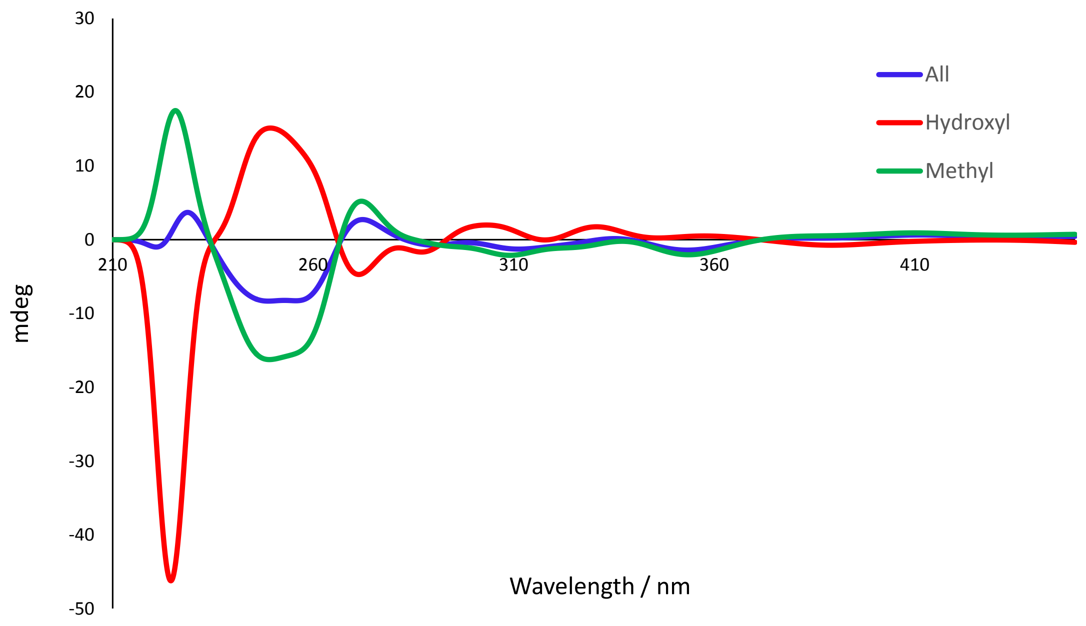

Figure S3. Un-shifted CD spectra for **3** calculated using the PBE1PBE functional. The "methyl" spectrum represents a weight averaged spectra for conformers with rotation about the C6-C7 axis, giving the *R*-isomer. The "hydroxyl" spectrum represents a weight averaged spectra for conformers with rotation about the C6-C7 axis, giving the *S*-isomer. The "All" spectrum combines all low energy conformers.

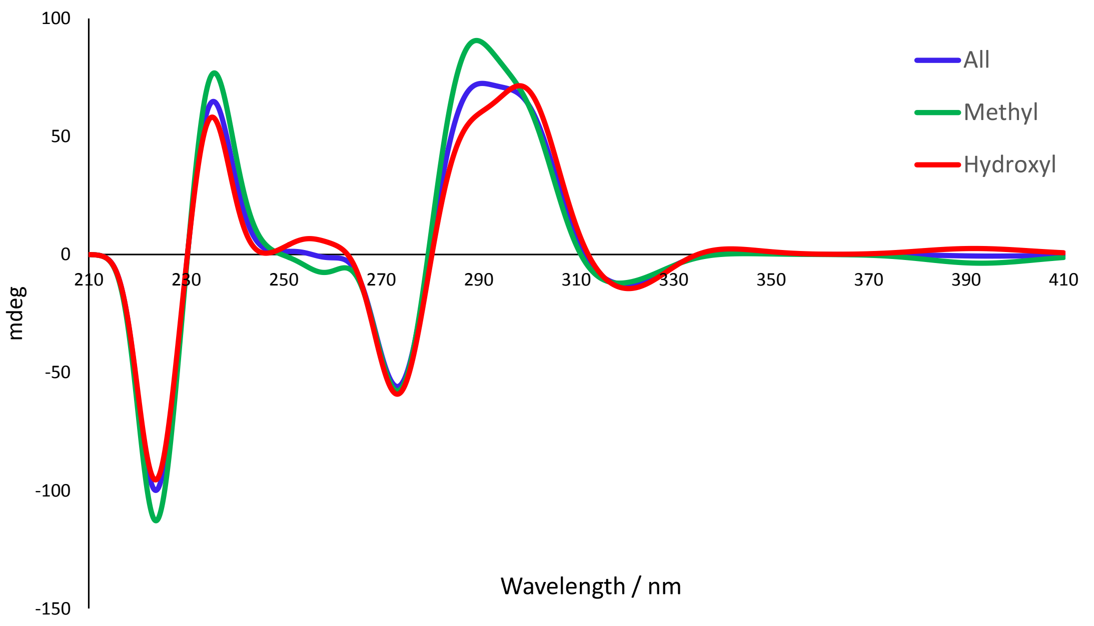

Figure S4. Un-shifted CD spectra for the (10*R*,19*R*) isomer of **5** calculated using the PBEPBE functional. The "methyl" spectrum represents a weight averaged spectra for conformers with rotation about the C6-C7 axis, giving the *R*-isomer. The "hydroxyl" spectrum represents a weight averaged spectra for conformers with rotation about the C6-C7 axis, giving the *S*-isomer. The "All" spectrum combines all low energy conformers.

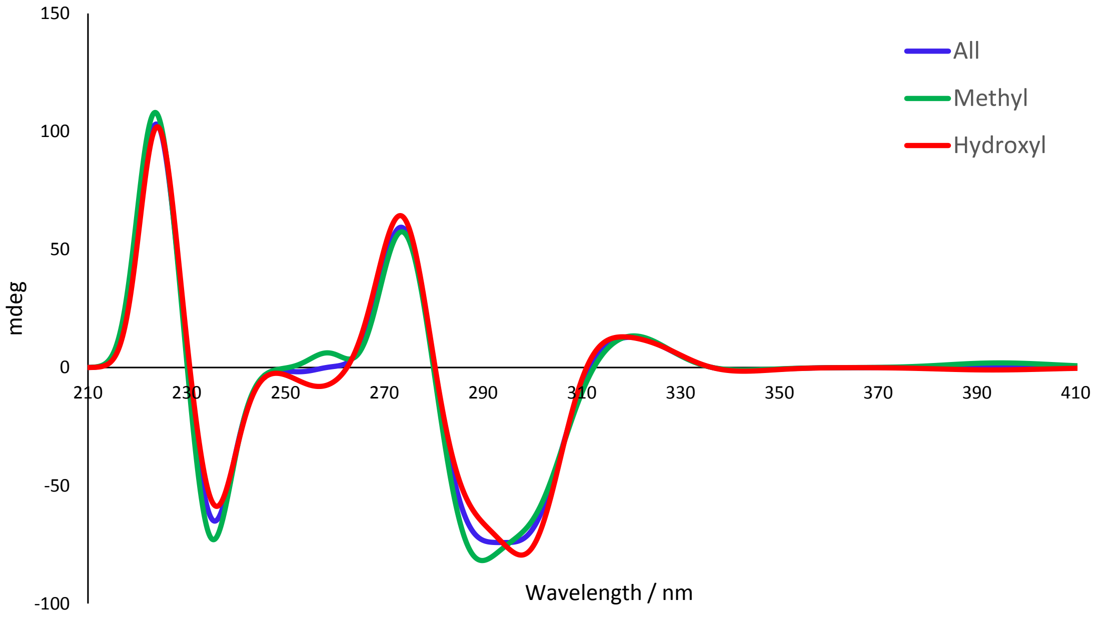

Figure S5. Un-shifted CD spectra for the (10*S*,19*S*) isomer of 5 calculated using the PBE1PBE functional. The "methyl" spectrum represents a weight averaged spectra for conformers with rotation about the C6-C7 axis, giving the *R*-isomer. The "hydroxyl" spectrum represents a weight averaged spectra for conformers with rotation about the C6-C7 axis, giving the *S*-isomer. The "All" spectrum combines all low energy conformers.

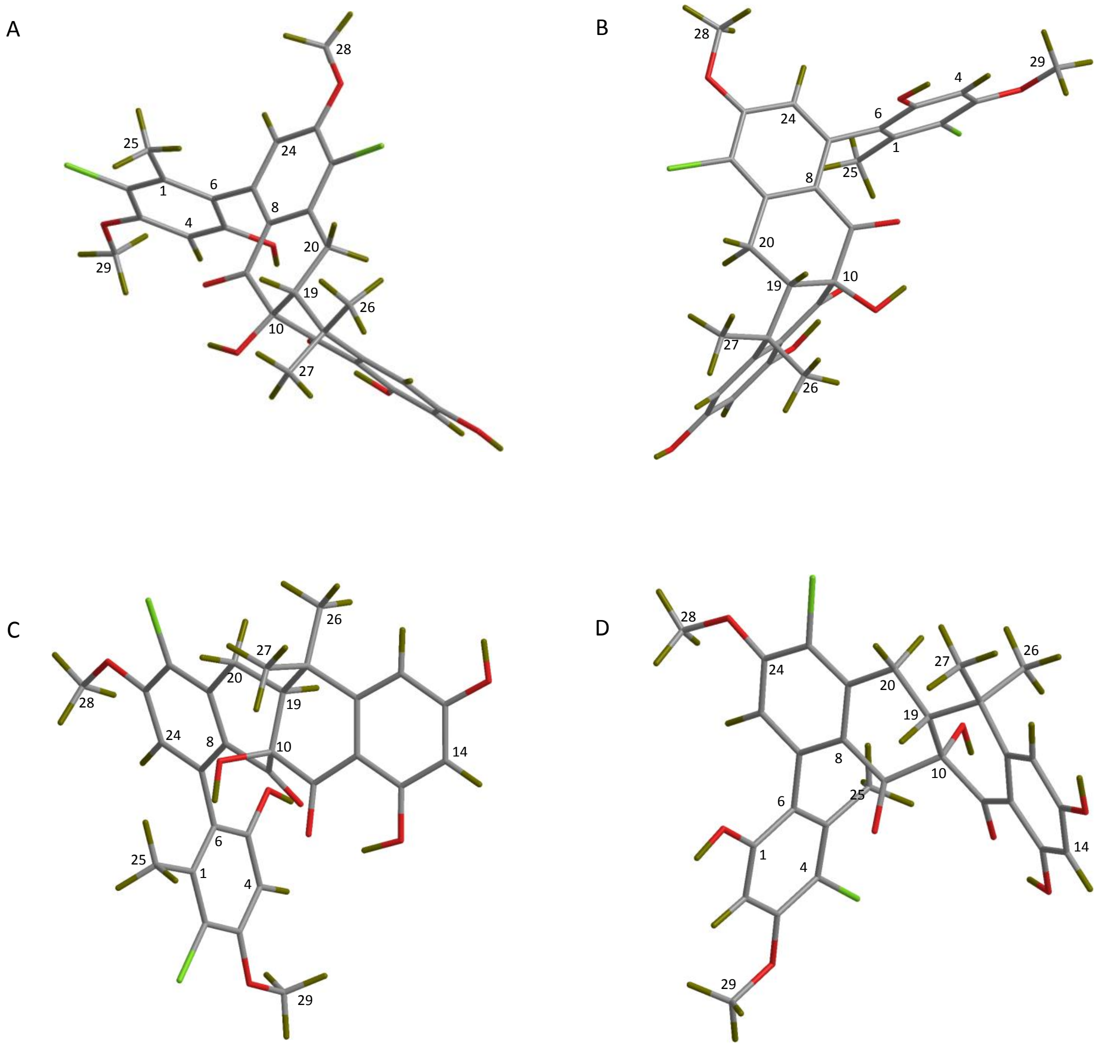

Figure S6. Modelled structures of (*10R*, *19R*)-**5** (A), (*10S*, *19S*)-**5** (B), (*10R*, *19S*)-**5** (C), and (*10S*, *19R*)-**5** (D).

3. ESI Tables

Table S1. Strains, plasmids and primers used in this work.

**Strain name**	**Description**	**Source or Ref.**
E. coli ET12567	*dam dcm-*	Reference 11
E. coli DH10ß	F– *mcr*A Δ(*mrr-hsd*RMS-*mcr*BC) Φ80*lac*ZΔM15 Δ*lac*X74 *rec*A1 *end*A1 *ara*D139 Δ(*ara leu*) 7697 *gal*U *gal*K *rps*L *nup*G λ–	Invitrogen, USA
E. coli Top10	F– *mcr*A Δ(*mrr-hsd*RMS-*mcr*BC) Φ80*lac*ZΔM15 Δ*lac*X74 *rec*A1 *end*A1 *ara*D139 Δ(*ara leu*) 7697 *gal*U *gal*K *rps*L *nup*G	Invitrogen, USA
Streptomyces formicae	Wild-type strain	Reference 12
Streptomyces formicae Δ*for*	Formicamycin (*for*; BGC30) deleted	This work
Streptomyces formicae Δ*for* + pESAC13-215-G	Formicamycin (BGC30) deleted and complemented with the *for* BGC	This work
**Plasmids and ePACs**	**Description**	**Source or Ref.**
pUZ8002	Non-transmissible RK2 derivative with a mutation in *oriT*	Reference 2
pR9604	pUB307 derivative	Reference 13
pESAC13-215-G	ePAC clone harbouring the *for* BGC	This work (generated by BioS&T, Montreal Canada

**Primer name**	**Sequence**	**Description**
KY5001 35KOFor1	ATCATCTAGAAAGGACATTCGC CTCGTCAGCCGCAAG	pCRISPomyces-2 template 1 left flank BGC
KY5002 35KORev1	GCTGCTGCGACCAGGCGAGCTC GCGTCGAGACGCAACTCAGTG AAACCTTG	pCRISPomyces-2 template 1 right flank BGC

KY5003 35KORev2	ACGTTCTAGAGAGGAACTCCTC ATAGGTGATCAGATAACC	pCRISPomyces-2 template 2 left flank BGC
KY5004 35KOFor2	GCGAGCTCGCCTGGTCGCAGCA GCGTACTGACAGACAATTTCTC CACGTTCGGC	pCRISPomyces-2 template 2 right flank BGC
KY5005 gRNA35For	ACGCGCGCCATGAAGCTAAGG AGG	BGC gRNA for
KY5006 gRNA35Rev	AAACCCTCCTTAGCTTCATGGC GC	BGC gRNA rev
KY5007 BGC_Left_Fwd	ACAGGTACGACGGGTCC	pESAC13 5' edge forward test primer for BGC
KY5008 BGC_Left_Rev	CCCAACCAGTACGCGAAG	pESAC13 5' edge reverse test primer for BGC
KY5009 BGC_Right_Fwd	GCATGGGATGTGAGCACC	pESAC13 3' edge forward test primer for BGC
KY5010 BGC_Right_Rev	AAGAGGCGATGAGCGAGG	pESAC13 3' edge reverse test primer for BGC
KY5011 BGC_Mid_Fwd	TACCACATCGGCGAGTCC	pESAC13 forward test primer for BGC centre
KY5012 BGC_Mid_Rev	CGCTCCAGGTTGTACGAC	pESAC13 reverse test primer for BGC centre
KY5013_6548	ATCGGTGAGATCACCATGACTA CGG	Confirmation of cluster deletion
KY5014_6507	GTTCGACGGTGCCGATGAAGC	Confirmation of cluster deletion
KY5015_6505	CTGTACGCTGACAGCCGGAAC	Confirmation of cluster deletion
KY5016_6514	GGCGAAGAGGCGGGCGATCTC G	Detection of cluster edge
KY5017_6512	CACGACAGACCCCTCCCGCGT	Detection of cluster edge
KY5018_6538	CATTCCCGGGGCCCGGGTGT	Detection of cluster edge
KY5019_6537	AGCCGACGGCGTATCGGCTGA CG	Detection of cluster edge
RD125 Halogenase Flank 1.1	gctcggttgccgccgggcgtttttaTCTAG Agacgagcacggatacgtgatcgg	pCRISPomyces-2 template 1 left flank ForV

RD126 Halogenase Flank 1.2	GCGAGCTCGCCTGGTCGCAGCAGC gtgacggcacaggagcagagc	pCRISPomyces-2 template 1 right flank ForV
RD127 Halogenase Flank 2.1	GCTGCTGCGACCAGGCGAGCTCGC tcactcatcgccgttcccttcc	pCRISPomyces-2 template 2 left flank ForV
RD128 Halogenase Flank 2.2	gcaacgcggcctttttacggttcctggccTCTAGAcgacgtgatgcggctcgactg	pCRISPomyces-2 template 2 right flank ForV
RD129 Halogenase gRNA F	acgcacgaccagtccttgatggta	ForV gRNA for
RD130 Halogenase gRNA R	aaactaccatcaaggactggtcgt	ForV gRNA rev
RD131 Halogenase genome KO F 1.1	ggacgagagccctcccagtg	Confirmation of ForV deletion
RD059 Halogenase Insert F1	gtcaaggcgcacgaggagctg	Confirmation of ForV deletion
RD132 Halogenase genome KO R 2.2	ccgatgtcgacgacggaggc	Confirmation of ForV deletion
RD060 Halognenase Insert R1	gactcgttcagggcgaagtgcc	Confirmation of ForV deletion
RD187 pMS82 KY5p For	gccgagaaccTAGGATCCAAGCTTcatggtgaggtgctcctcctg	Generation of ForV complementation plasmid
RD188 KY5P Halogenase compl Rev	ggcgctgctggtcactcatgtggagctgccctcactc	Generation of ForV complementation plasmid
RD189 KY5p Halogenase compl For	gagtgagggcagctccacatgagtgaccagcagcgcc	Generation of ForV complementation plasmid
RD190 Halognenase pMS82 compl Rev	CTGGTACCATGCATAGATCTAAGCTTtcaccggccccgctccacgg	Generation of ForV complementation plasmid
pMS82 Rev HindIII	gccagtggtatttatgtcaacaccgcc	Confirmation of ForV complementation strain
pMS82 For HindIII	gcaacagtgccgttgatcgtgctatg	Confirmation of ForV complementation strain

Table S2. BGCs identified in the *S. formicae* genome using antiSMASH3.0

BGC	Type	Similar cluster*	Comments
1	Nucleoside antibiotic	A-503083	-
2	Lantipeptide	None	Putative class 1; single A gene
3	NRPS-T1PKS	Unknown BGC in deposited genome sequence	Tripeptide + one ketide unit
4	Lantipeptide	None	Putative class 1; two A genes
5	NRPS	None	-
6	T3PKS	None	Mixed cluster
7	NRPS	None	Pentapeptide
8	NRPS	Calcium dependent antibiotic (CDA)	Dodecapeptide
9	terpene	2-methylisoborneol	2-methylisoborneol
10	Aminoglycoside	None	Phosphonate genes
11	NRPS	None	Decapeptide
12	NRPS	None	Pentapeptide
13	T1PKS-NRPS	None	-
14	T1PKS-lantipeptide	Abyssomicin	Putative class 1; single A gene; cluster directly adjacent to T1PKS
15	Terpene	None	-
16	Ectoine	Ectoine	Ectoine
17	NRPS-T1PKS-saccharide	None	-
18	Lantipeptide	SapB	SapB
19	Siderophore	Desferrioxamine B	Desferrioxamine B
20	Melanin	Unknown BGC in deposited genome sequence	-
21	NRPS	Griseobactin plus second NRPS	Griseobactin BGC; adjacent tridecapeptide NRPS
22	Ladderane-NRPS	Unknown BGC in deposited genome sequence	Mixed cluster
23	Terpene	None	-
24	Terpene	Unknown BGC in deposited genome sequence	Albflavenone BGC homologues present
25	Siderophore	Unknown BGC in deposited genome sequence	-
26	Butyrolactone	Methylenomycin like	-
27	Bacteriocin	Unknown BGC in deposited genome sequence	-
28	NRPS-T3PKS	None	Lipopeptide/glycopeptide similarity; DHPG subcluster; adjacent type 2 PKS like genes
29	Terpene	Unknown BGC in deposited genome sequence	-
30	T2PKS	Unknown BGC in *S. kanamyceticus*	Formicamycin BGC
31	NRPS-ladderane-T1PKS	None	-
32	Terpene	Unknown BGC in deposited	Hopene-like subcluster

		genome sequence	present
33	Lantipeptide-T3PKS-lassopeptide	None	Putative class 1; two A genes; mixed cluster/possibly two adjacent clusters
34	T1PKS	None	Novel polyene like structure
35	NRPS-T3PKS	None	-
36	NRPS	None	Novel glycopeptide like; DHPG subcluster
37	T1PKS	Lasaloic acid	Polyether like
38 & 39	Clavam	Clavam	Overlapping clusters comprising clavam cluster and T1PKS

* As judged after manual analysis of automated output

4. ESI References

1. E. S. Lennox, *Virology*, 1955, **1,** 190.
2. T. Kiese, M. J. Bibb, M. J. Buttner, K. F. Chater, and D. A. Hopwood, *Practical Streptomyces Genetics*, 2000.
3. T. Weber, K. Blin, S. Duddela, D. Krug, H. U. Kim, R. Bruccoleri, S. Y. Lee, M. A. Fischbach, R. Muller, W. Wohlleben, R. Breitling, E. Takano and M. H. Medema, *Nucleic. Acids. Res.*, 2015, **43**, 237.
4. R. F. Seipke, 2015, *PLoS ONE,* **10,** e0116457.
5. R. C. Edgar, *Nucleic Acids Res*, 2004, **32,** 1792.
6. M. N. Price, P. S. Dehal and A. P. Arkin, *PLoS ONE*, 2010, **5,** e9490.
7. Y. Guo, W. Zheng, X. Rong and Y. Huang, *Int. J. Syst, Evol, Microbiol.*, 2008, **58,** 149–159.
8. R. E. Cobb, Y. Wang and H. Zhao, *ACS. Synth.Biol.*, 2015, **4**, 723.
9. A. C. Jones, B. Gust, A. Kulik, L. Heide, M. J. Buttner and M. J. Bibb, *PLoS ONE*, 2013, **8**, e69319.
10. J. M. Moore, E. Bradshaw, R. F. Seipke, M. I. Hutchings and M. McArthur, *Methods Enzymol.,* 2012, **517**, 367.
11. D. J. MacNeil, K. M. Gewain, C. L. Ruby, G. Dezeny, P. H. Gibbons and T. MacNeil, *Gene*, 1992, **111**, 61.
12. R. F. Seipke, J. Barke, D. Heavens, D. W. Yu and M. I. Hutchings, *Microbiology Open*, 2013, **2**, 276.
13. J. C. Piffaretti, A. Arini and J. Frey, *Mol. Gen. Genet.,* 1998, **212**, 215.
